# Reference genome bias in light of species-specific chromosomal reorganization and translocations

**DOI:** 10.1186/s13059-025-03761-w

**Published:** 2025-10-15

**Authors:** Marius F. Maurstad, Siv Nam Khang Hoff, José Cerca, Mark Ravinet, Ian Bradbury, Kjetill S. Jakobsen, Kim Præbel, Sissel Jentoft

**Affiliations:** 1 Centre for Ecological and Evolutionary Synthesis, Department of Biosciences, University of Oslo, Oslo, Norway; 2 Department of Bioinformatics and Genetics, Swedish Museum of Natural History, Stockholm, Sweden; 3 SciLifeLab, Karolinska Institutet Science Park, Solna, Sweden; 4 Fisheries and Oceans Canada, Newfoundland, St John’s, Canada; 5 Norwegian College of Fishery Science, The Arctic University of Norway, Tromsø, Norway

**Keywords:** Genome assemblies, Reference bias, Comparative genomics, Population genetic measurements, Inversion scoring

## Abstract

**Background:**

Whole-genome sequencing efforts, have during the past decade, unveiled the central role of genomic rearrangements—such as chromosomal inversions—in evolutionary processes, including local adaptation in a wide range of taxa. However, employment of reference genomes from distantly or even closely related species for mapping and the subsequent variant calling can lead to errors and/or biases in the datasets generated for downstream analyses.

**Results:**

Here, we capitalize on the recently generated chromosome-anchored genome assemblies for Arctic cod (*Arctogadus glacialis*), polar cod (*Boreogadus saida*), and Atlantic cod (*Gadus morhua*) to evaluate the extent and consequences of reference bias on population sequencing datasets (approx. 15–20 × coverage) for both Arctic cod and polar cod. Our findings demonstrate that the choice of reference genome impacts the mapping statistics, including mapping depth and mapping quality, as well as core population genetic estimates, such as heterozygosity levels, nucleotide diversity (π), and cross-species genetic divergence (D_XY_). Furthermore, using a more distantly related reference genome can lead to inaccurate detection and characterization of chromosomal inversions, i.e., in terms of size (length) and location (position), due to inter-chromosomal reorganizations between species. Additionally, we observe that some of the verified species-specific inversions are split across multiple genomic regions when mapped against a heterospecific reference.

**Conclusions:**

Inaccurate identification of chromosomal rearrangements as well as biased population genetic measures could potentially lead to erroneous interpretation of species-specific genomic diversity, impede the resolution of local adaptation, and thus, impact predictions of their genomic potential to respond to climatic and other environmental perturbations.

**Supplementary Information:**

The online version contains supplementary material available at 10.1186/s13059-025-03761-w.

## Background

Recent advancements within sequencing technologies and bioinformatic tools have revolutionized the field of biology. Pioneering studies have been conducted within human genomics, which have improved our understanding of biological processes tremendously. The number of studies on wildlife and marine species is also increasing [1–4], and over the past years, several larger international initiatives have been established to characterize all of life’s genomic diversity [5]. Within these efforts, the overall goal is to generate highly contiguous reference genomes (i.e., chromosome level) that can be used in a i) comparative setting to describe the genomic diversity between species, and/or ii) conduct within-species genome-wide characterization of cryptic ecotypes and sub-population differentiations [4–7].

While the number of high-quality reference genomes is steadily increasing, the distribution of available references differs among taxonomic groups, and thus, there is still a shortage in the number of references for various taxa [8]. In cases where a reference genome for the focal species is missing, the standard method is to select a close relative for mapping and subsequent variant calling [9]. Even if this practice, i.e., using a reference genome from a closely (or more distantly) related species and/or a divergent population, by large, facilitates valuable insight into the genetic makeup and structure of the focal species; there is a growing body of literature that lists several shortcomings [9–14]. Genomic divergence between the reference and the target species (or population) can hamper the mapping efficiency, as well as the subsequent variant calling and downstream population genetic inferences [9–14]. For instance, inaccurate measures of genetic variability, i.e., overestimation of nucleotide diversity and heterozygosity—both important parameters for conservation genomics—have been documented when more divergent and/or non-conspecific references are employed [10–13]. However, few studies have examined how discrepancies in genomic architecture between the reference and target species would impact the identification of e.g., larger structural variants, such as chromosomal inversions. Since the beginning of the genomics era, chromosomal inversions have been recognized as part of the standing genomic variation of a species and/or sub-populations/ecotypes that are likely to play important roles in evolutionary processes, including local adaptation [15–20]. For instance, in Atlantic cod (*Gadus morhua*; L., 1758), four larger chromosomal inversions are found to discriminate between populations throughout its geographical distribution, i.e., dominating the observed genomic divergence by large allele frequency shifts [15, 21–24]. It is suggested that these are of high importance for maintaining the genomic divergence between locally adapted populations, including the iconic migratory Northeast Arctic cod (NEAC) and the more stationary Norwegian coastal cod (NCC) [15, 21–24]. However, such and other structural variants could be overlooked or inadequately characterized due to larger or smaller inter-chromosomal reorganizations between the reference used and the actual genome of the focal species. For instance, a previous study identified discrepancy in the number of putative chromosomal inversions detected for the European plaice (*Pleuronectes platessa*) when using the species-specific reference vs. using the closely related Japanese flounder (*Paralichthys olivaceus*) as the reference [25, 26]. This inconsistency could potentially be due to species-specific differences in i) number of inversions and/or ii) other types of inter-chromosomal reorganizations.

**Fig. 1:**
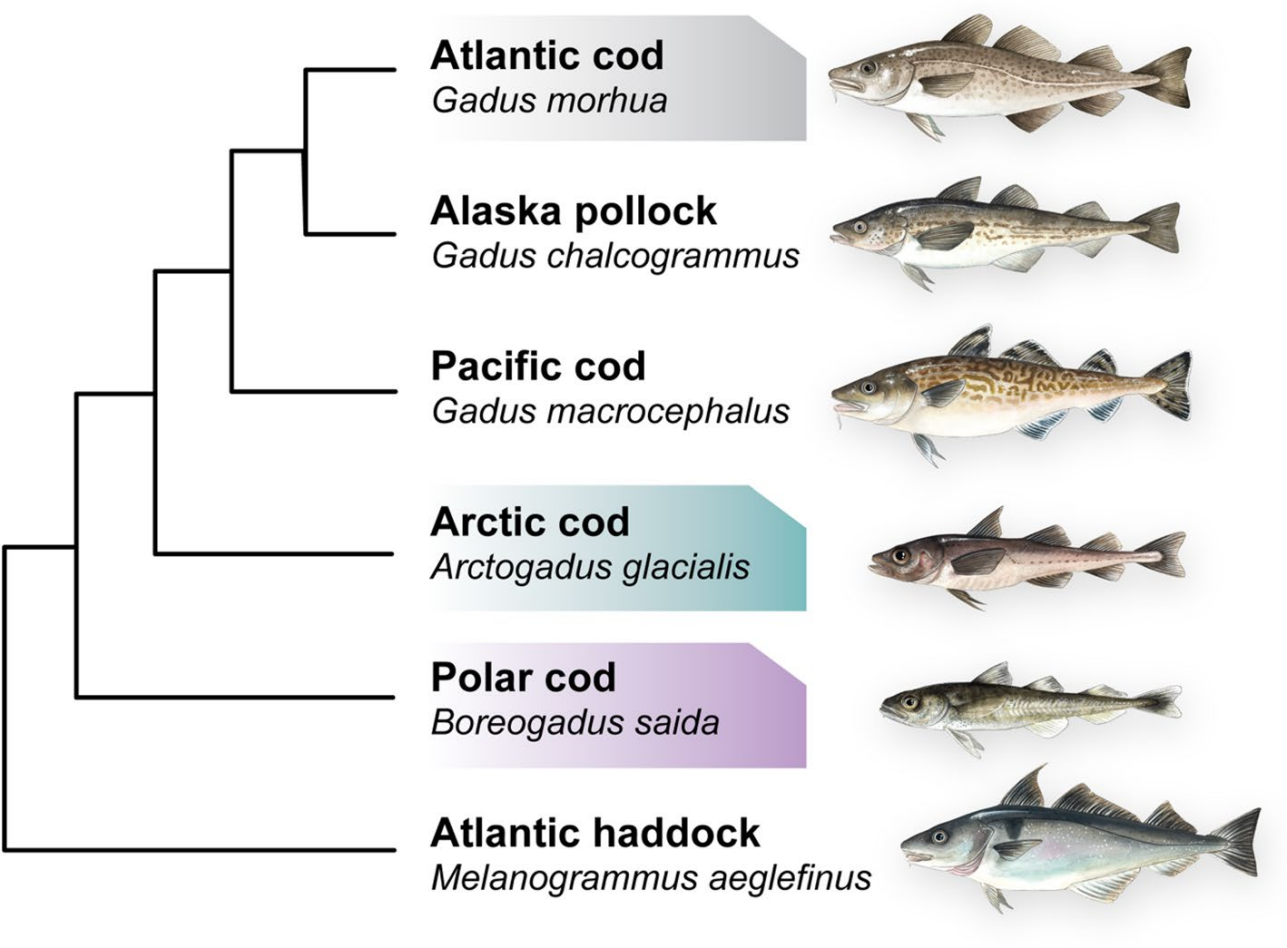
A cladogram of the phylogenetic relationship of selected species within the gadidae lineage, including the focal species of this study (Arctic cod and polar cod), redrawn from Matschiner et al. [24] and Hoff et al. [27]. The phylogenetic placement of Arctic cod is not fully resolved, as it may be either a sister lineage to Gadus or a sister species to polar cod [24, 27]. The three species used as reference genomes are highlighted: Atlantic cod in grey, Arctic cod in green, and polar cod in purple. The fish illustrations are made by Alexandra Viertler

**Fig. 2:**
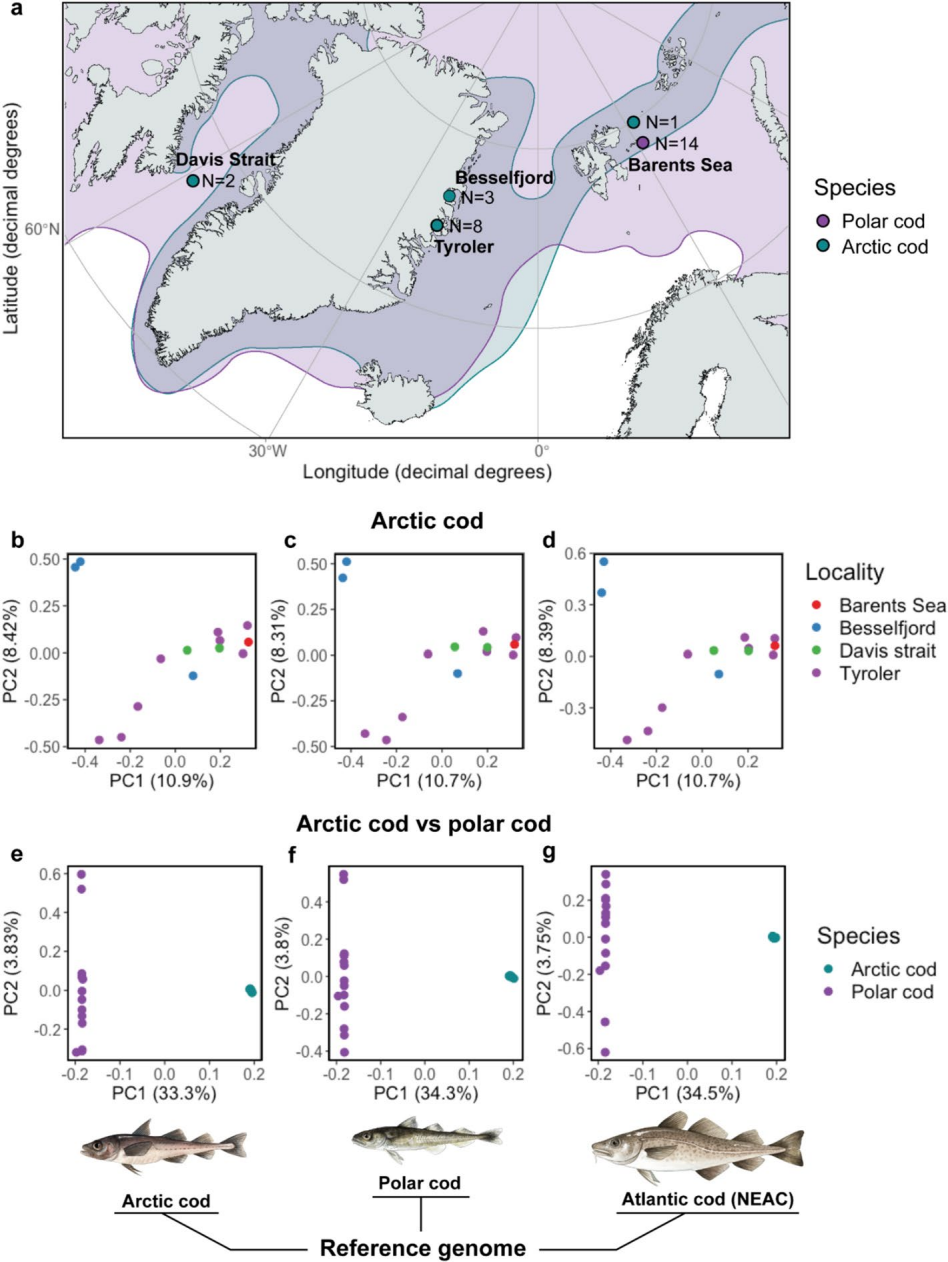
Geographical distribution, intraspecific and cross-species genetic structuring for Arctic cod and Arctic cod vs. polar cod using three different reference genomes. **a** Map displaying the geographical distribution of Arctic cod and polar cod in the sampling region (redrawn from Mecklenburg et al. [40]) where the sample locations and sizes are denoted. **b**, **c**, **d** PCA displaying the two first principal components for the Arctic cod individuals (*N* = 14) using the three references, i.e., Arctic cod, polar cod, and Atlantic cod (NEAC) and **e**, **f**, **g** PCA displaying the two first principal components for the cross-species datasets, i.e., Arctic cod (*N* = 14) vs. polar cod (*N* = 14), using the three references. The fish illustrations are made by Alexandra Viertler

Within the gadidae lineage, major genomic reorganizations and reshufflings have been documented, especially within the two cold-water specialists: the Arctic cod (*Arctogadus glacialis;* Peters, 1872) and the polar cod (*Boreogadus saida;* Lepechin, 1774) [27]. Additionally, for polar cod, a large number of polymorphic chromosomal inversions (with a potential impact on sub-population structuring) have been detected [28]. Such major genomic reorganizations and reshufflings could potentially lead to downstream bioinformatic errors in mapping, variant calling, and data interpretation, depending on the reference used. In this study, we aimed at taking full advantage of the newly generated chromosome-anchored genome assemblies of the closely related Arctic cod [27], polar cod [27], and Atlantic cod (NEAC) [29] (Fig. 1) to assess how a species-specific vs. a heterospecific reference genome would impact the mapping statistics, as well as the core population genetic estimates, including heterozygosity level and measures of population differentiation and divergence between Arctic cod and polar cod when exploring population-level data of the two species collected from the northern Barents Sea and adjacent regions (Fig. 2a). Additionally, we investigated how the different reference genomes influence the detection of chromosomal inversions, focusing exclusively on the Arctic cod. Both Arctic cod and polar cod represent important sympatric species inhabiting the Arctic, one of the world’s most rapidly changing environments that is undergoing warming at a pace almost four times faster than the global average [30]. Until recently, the studies that have looked into the population genetic structuring of Arctic cod and polar cod have only used a handful of genetic markers [31–36], however, there are now emerging attempts using whole genome sequencing approaches [28, 37–39], and by such, this study will advance our insight into the interpretation of the genomic composition and potential within these species in light of the ongoing climatic changes.

## Results and discussions

### Genetic structure of Arctic cod and Arctic cod vs. polar cod

The Principal Component Analysis (PCA) conducted on the *intraspecific* genomic dataset revealed a separation among the Arctic cod specimens along the first principal component (PC1) axis, explaining 10.7–10.9% of the variation in the datasets depending on the reference used (Fig. 2b–d). Additionally, a separation along the PC2 axis was demonstrated, explaining 8.31–8.42% of the variation in the datasets (Fig. 2b–d). When inspecting this separation against the various variant calling statistics (Additional file 1, Figure S1–S3), we found that neither mean depth nor presence of missing sites appeared to have a notable influence on the positioning of the samples within the PCA. Mean depth was generally consistent across most samples (averaging 15–20 × coverage), except for a single individual from Davis Strait. This individual stems from the same collection as the other sample from Davis Strait, but was sourced from a publicly available dataset (Additional file 2, Table S1), which was sequenced to a greater depth (approx. 30 × coverage) compared to the rest of the samples included in this study. Furthermore, among the samples, one individual from Besselfjord displayed a higher degree of missing data compared to the rest. The positioning of these two samples was neither impacted by the increased sequencing depth nor missingness. The proportion of heterozygous sites, however, tended to overlap to some degree with the positioning of the samples along the PC1 axis. But this was not the case for all samples, for instance; the Davis Strait sample (with the highest coverage and highest proportion of heterozygotic sites present) was placed in the middle of the gradient (see Additional file 1, Figure S1–S3). It should also be mentioned that the proportion of heterozygous sites was generally higher using either polar cod or NEAC vs. Arctic cod as reference. This difference in levels of heterozygosity did not impact the placement of the samples within the PCA (Fig. 2b–d; Additional file 1, Figure S1–S3), which had more or less the same positioning irrespective of the reference used. It is therefore tempting to speculate that the positioning of the samples is associated with some sort of sub-population structuring within Arctic cod. However, to fully assess this and define the different sub-populations, a larger dataset with more individuals from a larger geographical range is needed.

The PCAs on the *cross-species* datasets uncovered, as expected, a distinct clustering pattern irrespective of the reference used, where the samples were separated along the first principal component (PC1 axis) into two clusters (Fig. 2e–g), i.e., one cluster for Arctic cod and one cluster for polar cod, respectively. Notably, a difference in how the two species clustered along the second principal component (PC2 axis) was also identified, with Arctic cod exhibiting minimal intraspecific variation, whereas polar cod displayed intraspecific variability along the PC2 axis (Fig. 2e–g), explained by 3.75–3.83% of the variation in the datasets, depending on the reference used.

Taken together, our findings demonstrate that the genetic variation uncovered by the PCA analyses conducted both on the *intraspecific* and the *cross-species* datasets was not to any larger extent impacted by the reference used. Similar results—where the variance explained by the first and second principal components was comparable irrespective of the choice of reference—have been documented in a study where they looked into the potential reference bias when employing a dog vs. wolf reference genome for variant calling for Canis spp. population genomics [11]. These observations are likely coupled to the fact that a larger portion of the common variants between the focal species is the majority of variants detected, and used by standard population genetic analyses, including PCA and admixture analyses [11]. For instance, the main clustering patterns in the *cross-species* dataset were associated with the genetic divergence between the species as well as the larger standing genetic variation detected in polar cod vs. Arctic cod. The latter observation could be linked to the difference in female N_e_ observed between the species (see Additional file 3, Figure S4 and Hoff et al. [28] as well as documented by others [37, 41, 42]). For *intraspecific* datasets targeting within-species differentiation, however, reference bias could potentially play a larger role, especially in terms of accurate calling of rare and/or more private alleles. The lack of detection of such a reference bias in our dataset for Arctic cod could be coupled to an overriding sub-population structure within the species, as mentioned above.

### Impact of reference genome on mapping and variant calling statistics

**Fig. 3:**
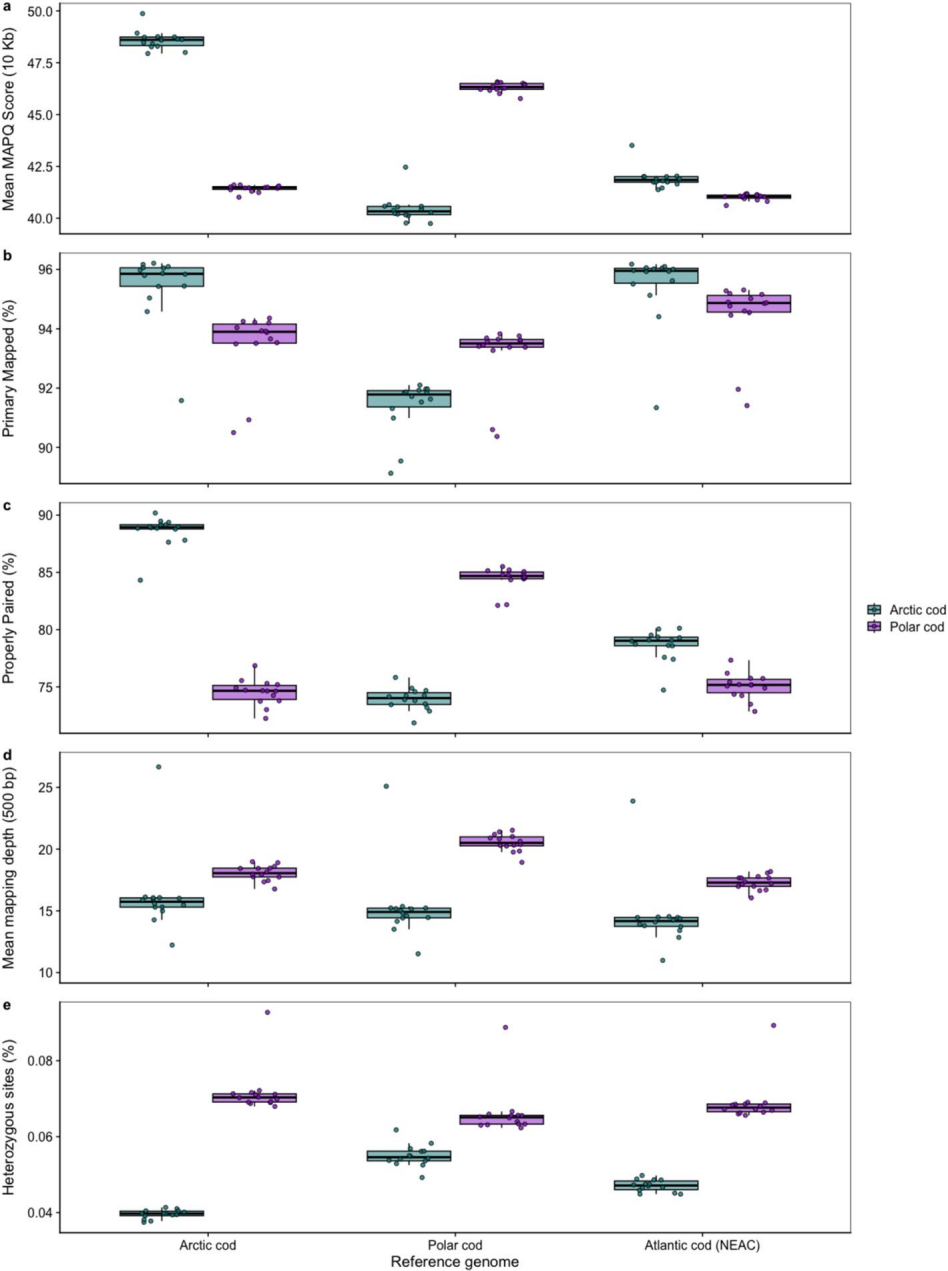
Variability in mapping statistics and heterozygosity measurements for the cross-species comparisons using the three different references for Arctic cod, polar cod, and Atlantic cod (NEAC). **a** Mean MAPQ scores (average mapping quality across genomic windows) for Arctic cod and polar cod samples based on the reference used, **b** primary mapped percentage (proportion of reads mapping as primary alignments), and **c** properly paired percentage (fraction of paired-end reads mapping in proper orientation and insert-size). MAPQ scores were computed using bedtools to calculate mean mapping quality within genomic windows, then averaged per sample across primary chromosomes for each sample mapped against each reference. **d** Differentiation in the mean mapping depth for Arctic cod and polar cod samples based on the reference used. The highest mean depth is seen in samples when they are mapped against their respective intraspecific references. **e** The proportion of heterozygous sites per sample for the two species and how it changes with the reference used. The lowest values are found in Arctic cod and polar cod when using the intraspecific reference

For the *cross-species* datasets, the estimation of mean mapping depth as well as mapping quality uncovered a species-specific variability, which was dependent on the reference genome used. We detected highest mean depth and MAPQ scores (average mapping quality across genomic windows for primary chromosomes) when individual sequencing data were mapped against the intraspecific reference, whereas employment of a heterospecific reference, i.e., one of the two other codfishes, resulted in lower measurements (Fig. 3a, b, c, and d). For the Arctic cod dataset, the lowest mapping depth was observed using NEAC as the reference while the lowest MAPQ values were observed when using polar cod as the reference. For the polar cod dataset, the mapping statistics, i.e., mapping depth as well as MAPQ values, were similarly low when using either of the heterospecific references (Fig. 3a, b, c, and d). Our findings—if aligned to the sequence similarity measurements (i.e., Mash [43] distance estimations, see Additional file 4, Figure S5a)— indicate that the mappability is strongly coupled to the genome distance between the focal species and the reference used. The highest Mash distance was observed between Atlantic cod and polar cod, followed by the distance measured between Arctic cod and polar cod (Additional file 4, Figure S5a). These findings indicate that the assumed closely related cold-water adapted species [24] have likely undergone larger species-specific genomic reorganizations since they branched off from their common codfish ancestor. Arctic cod and Atlantic cod displayed, on the other hand, a higher degree of sequence similarity compared to polar cod (Additional file 4, Figure S5a). Notably, the overall higher depth recorded for the polar cod datasets irrespective of reference used was due to the fact that the polar cod samples were sequenced in a separate batch with slightly higher coverage (see Supplementary Materials and Methods in Hoff et al. [28]).

Moreover, the proportion of heterozygous sites per sample (Fig. 3e) aligned well with the patterns of mapping quality (and to some extent the mean depth) observed. The lowest number of heterozygous sites was detected when the intraspecific reference was employed (Fig. 3e), while a higher proportion of heterozygous sites was detected when one of the two heterospecific codfishes was used as the reference (Fig. 3e). These findings indicate that more accurate and higher mappability—when using an intraspecific reference—are likely resulting in a lower proportion of erroneously called heterozygous sites vs. when using a heterospecific reference. Intriguingly, the degree of heterozygosity seemed to be less impacted when using NEAC as a reference. These more intermediate measurements (Fig. 3d) are likely coupled to the intermediate genomic distance from NEAC to both of the more cold-water adapted species (Additional file 4, Figure S5a) and thus, the intermediate MAPQ scoring observed when using NEAC as the reference (Fig. 3a).

Additionally, our mapping statistics and heterozygosity level measurements could potentially be impacted by the quality of the reference used. In our study, the NEAC reference (i.e., the gadMor3.0 genome assembly) is of higher quality and more contiguous compared to the Arctic cod and the polar cod genome assemblies [27, 29]. Thus, mapping towards these lower quality genomes could potentially result in a higher degree of erroneous mapping of reads, i.e., misalignments, especially due to the high genomic content of short tandem repeats which is detected within the codfishes [44–46]. Accordingly, the lower quality of the Arctic cod and the polar cod genomes, i.e., with a lower resolution of the repetitive regions, combined with lower sequence identity between these two species, could result in lower quality of the mapping, especially within repetitive regions, and thus, easily result in a higher degree of wrongly called heterozygous sites [10–14, 47].

By taking advantage of the newly generated Oxford Nanopore draft genome assembly of polar cod (v2; fBorSai1.1.draft.fa.gz) and the newly released genome assembly of the coastal cod ecotype (Hoff et al. [27]), both identified to be of higher quality than the other three references used in this study (see Additional file 4, Figure S5b), we were able to assess the impact of the quality of a genome in respect to the mapping metrics and/or coupled to poorer mapping in high repetitive regions. Between the Atlantic cod references, the NCC and NEAC, the higher quality NCC assembly had the best MAPQ scores (Additional file 4, Figure S5c). Similar measurements were observed for the new and better polar cod assembly (v2) when compared to the older polar cod assembly (v1). Interestingly, Arctic cod samples showed higher primary mapping rates to the new polar cod genome than to their conspecific reference (Additional file 4, Figure S5d). This finding is likely due to the higher completeness of this genome assembly, which could be coupled to the Oxford Nanopore reads spanning longer complex genomic regions than the PacBio reads. However, properly paired read mapping (Fig. 3c; Additional file 4, Figure S5e) revealed that each species mapped best to their respective genome, indicating that read pairs had difficulty mapping with proper orientation and insert size when using more divergent references. Arctic cod samples mapped better to the Atlantic cod genomes compared to both of the polar cod genomes, which is consistent with the clustering observed in our Mash analysis (Additional file 4, Figure S5a). Moreover, we found that conspecific references maintained good mapping coverage even in high-repeat regions. In contrast, when using heterospecific references, a substantially reduced mapping coverage was observed in windows with increasing repeat content (Additional file 4, Figure S6), indicating a higher sequence divergence between the species for such complex/repetitive regions. The reduced/fragmented mapping coverage is likely coupled to a higher degree of collapsed reads for some parts of these regions and/or a lower number of reads for other parts, which again results in poorer resolution, and thus, higher proportions of erroneously called heterozygous sites when using a heterospecific reference (as shown in Fig. 3e). Taken together, both assembly quality and sequence divergence/distance of the reference used from the focal species impact mapping outcomes and downstream variant calling accuracy.

**Fig. 4:**
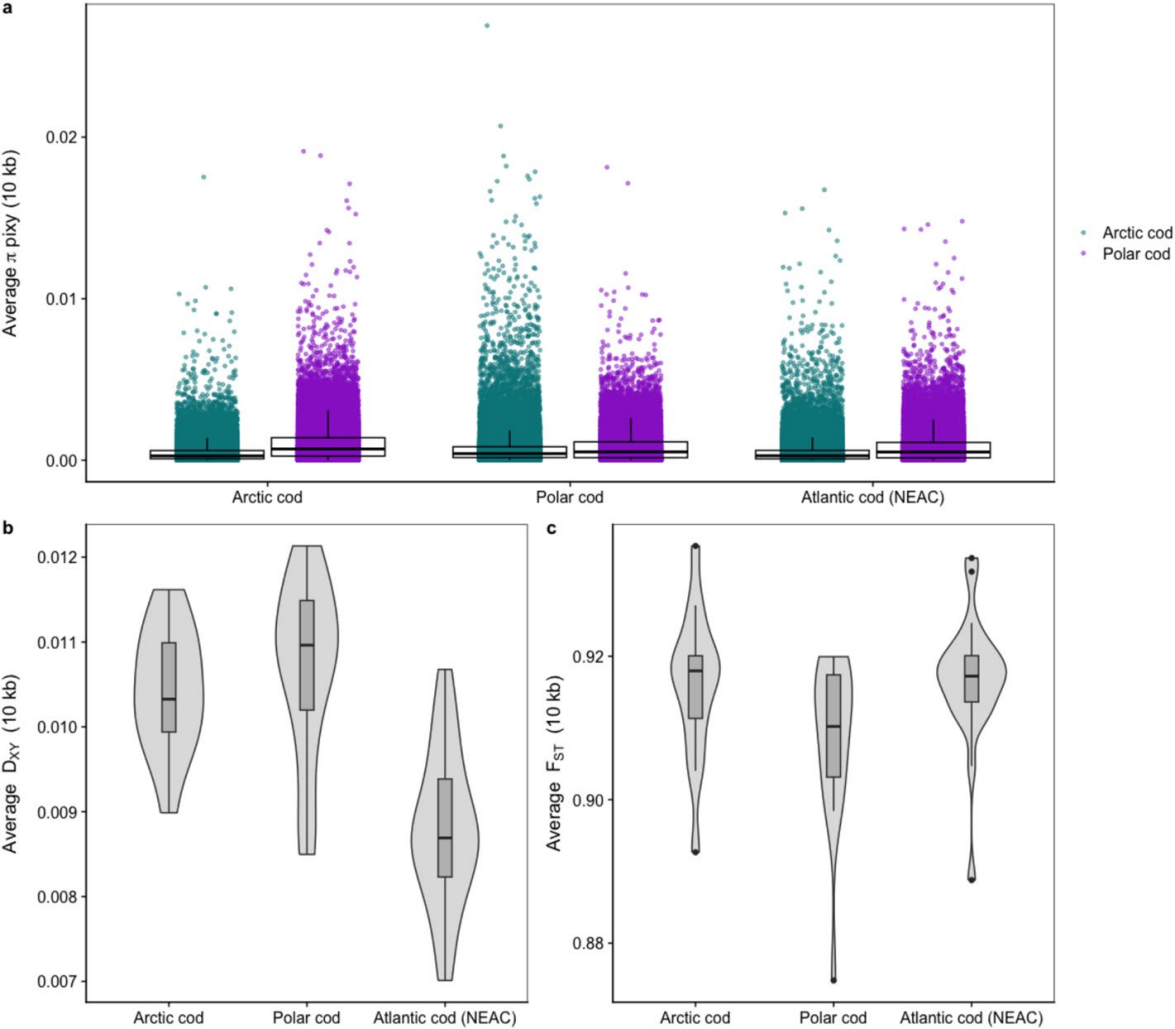
Variability in sample statistics and population estimates for the cross-species comparisons using the three different references Arctic cod, polar cod, and Atlantic cod (NEAC). **a** Average π values estimated in windows when using the three different reference genomes, demonstrate variation in calculated π values. **b**, **c** Average D_XY_ and F_ST_ for each chromosome in the cross-species comparison of Arctic cod and polar cod, using the three different references, also show variability depending on the reference chosen

For the additional population genetic statistics calculated for both of the species, we discovered varying results depending on the reference used (Fig. 4a–c). The window-based average π estimates unraveled more or less similar overall trends regardless of the reference genome used (Fig. 4a; box plots). However, when using the log-scale, subtle differences could be detected, e.g., that polar cod has an overall higher nucleotide diversity irrespective of the reference used compared to Arctic cod (see Additional file 4, Figure S7). Notably, we also observe that the nucleotide diversity was lowest when using the species-specific reference (see Additional file 4, Figure S7). Moreover, when either Arctic cod or polar cod was used as a reference, the non-conspecific reference exhibited a tailing of the average π values (Fig. 4a; points). In contrast, using NEAC as a reference, the tailing appeared less pronounced and more similar to the estimates seen for the intraspecific comparisons (Fig. 4a; points). Similarly, the average background D_XY_ divergence (Fig. 4b) between the species was higher when Arctic cod and polar cod were used as references, while a notable decrease in genetic divergence was observed when employing NEAC as the reference. These observations combined could probably also be linked to the difference in the quality of the genome assemblies as well as coupled to the genomic divergence, with the NEAC having the highest quality and lower degree of misalignments and, thus most likely less noisy/wrongly called variants in the datasets. It should also be mentioned that the employment of an equally distant relative as a reference for both species could here be an asset, i.e., not introducing any reference bias towards one of the species when performing the variant calling. Such a bias could most likely influence the genetic diversity detected between the two species, seemingly resulting in an overestimation of the genetic divergence between Arctic cod and polar cod when compared to the results achieved when using NEAC as the reference. On the other hand, when using NEAC as the reference, there might be a higher chance that the polymorphic sites detected between the two species are located within conserved regions (where the mappability is better), which could lead to an underestimation of the genetic divergence, as observed in our comparisons (Fig. 4b). Contrary to heterozygosity levels, π and D_XY_, the calculation of average background F_ST_ differentiation between Arctic cod and polar cod uncovered a similarly high degree of fixation between the species, irrespective of which of the three references that was used (Fig. 4c). The rather large interspecific differentiation at the genome-wide level corroborates the findings from the PCA analyses (Fig. 2e, f, and g), indicating that the reference used does not impact the variant calling to any degree for the detection of the global differentiation between the species when using F_ST_ and/or PCA analyses. In contrast, genetic diversity and genetic divergence, measured by heterozygosity levels, π and D_XY_, are seemingly more sensitive to the choice of reference used.

### Detection of multiple chromosomal inversions in Arctic cod

**Fig. 5:**
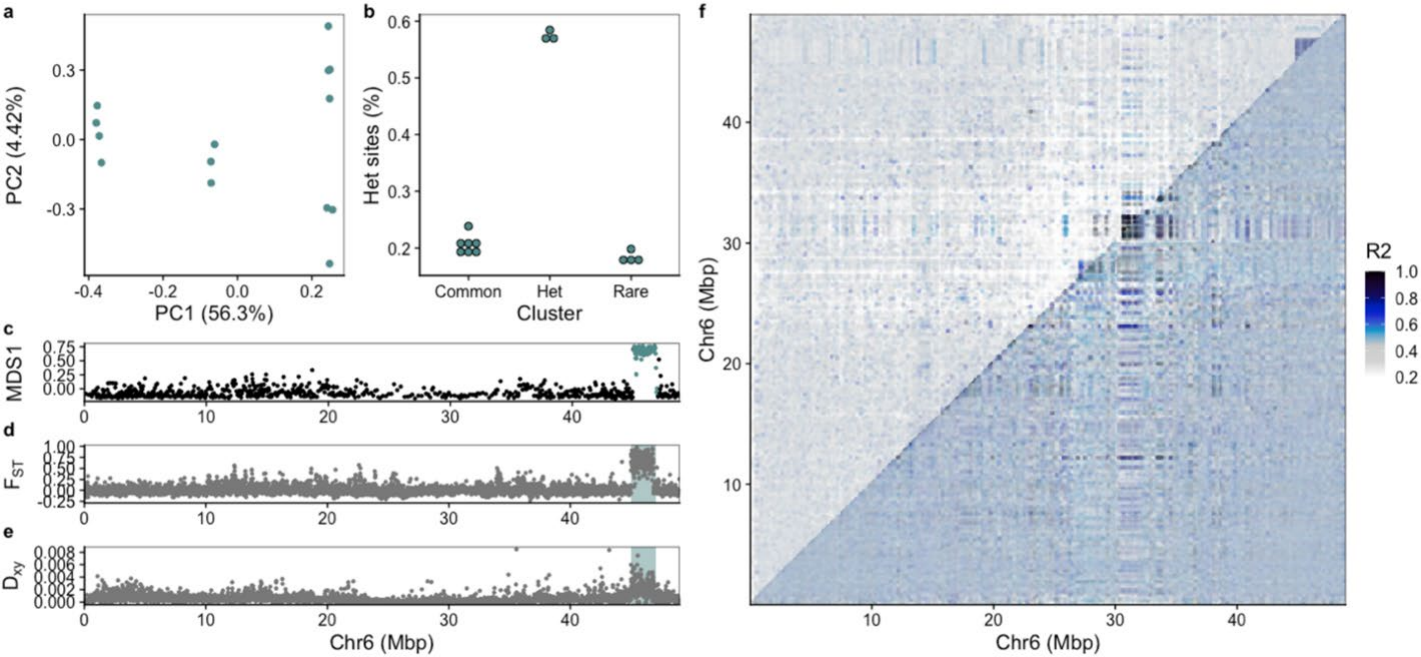
The inversion validation process of how a chromosomal inversion was detected, exemplified with the inversion on chromosome 6 when using Arctic cod as reference. A more detailed outline is illustrated in Figure S8. **a** PCA of the inversion region (identified in c) using lostruct. **b** Manual assignment of the hom-het-hom clusters where the heterozygous sites are given in bins for the respective clusters. **c** Multidimensional scaling (MDS) analysis produced by lostruct where the inversion region is highlighted. **d** F_ST_ and **e** D_XY_ between the rare and common clusters along the chromosome to assess patterns of genetic differentiation and divergence outside and inside the inversion regions, i.e., showing elevated values within the highlighted inversion region. The inversion region used for the identification steps (PCA and MDS plots) is marked in blue/green. **f** Plot of pairwise LD measured as R2. The top triangle includes all samples, and the lower triangle includes only the individuals with the common type. The upper right corner of the top triangle shows elevated R2 values within the defined inversion region; however, the bottom triangle, containing only individuals with homokaryotypes of the common type, does not display elevated R2 values. This pattern is in line with what is expected for a chromosomal inversion

For the *intraspecific* dataset when using Arctic cod as reference genome we detected six chromosomal inversions that fulfilled the criteria defined by our inversion detection protocol (Additional file 5, Figure S8). The six inversions were located on chromosome 1, 6, 10, 11, 13, and 14, spanning from 2 to 14 Mb in size (Table 1; Fig. 5; Additional file 5, Figure S9–S13). Furthermore, we identified five additional putative inversions, i.e., regions displaying more or less the same patterns as the other inversions, but with either weaker linkage disequilibrium (LD) signals, less clear heterozygosity levels defined for the heterokaryotypes, and/or only two or fewer individuals in the rare cluster (Table 1; Additional file 5, Figure S14–S20). Two of the smaller putative inversions represented a special case with their partial linkage and co-localization at the center of chromosome 7. Both of the regions exhibited inversion signals but did not share the same individuals between the three hom-het-hom clusters, and only two or one individual(s) were detected for the rare clusters (Additional file 5, Figure S14 and S15). Based on these findings, combined with the absence of any inversion signal in the intermediate region, the identified regions were denoted as two independent/separate putative inversions (Additional file 5, Figure S16). For the putative inversion identified on chromosome 9, the three clusters representing the hom-het-hom karyotypes respectively were detected; however, the region did not display any distinct, i.e. elevated, R2 values along the LD heatmap (Additional file 5, Figure S17). Lastly, the putative inversions detected on chromosomes 3 and 15 both fulfilled all the steps of the inversion detection protocol (see Additional file 5, Figure S8 for more details), except in terms of numbers of individuals in the rare cluster. Both inversions had only a single individual identified in the rare cluster (Additional file 5, Figure S18 and S19). Additionally, for chromosome 10, we identified a region upstream of the putative inversion that also displayed a high degree of differentiation (Additional file 5, Figure S20). However, this upstream region lacked the distinct PCA clusters and typical heterozygosity distribution expected for inversions (Additional file 5, Figure S20) and therefore was not classified as part of this inversion nor as a separate putative inversion.

**Table 1:** The identified chromosomal inversions for Arctic cod using the three reference genomes; Arctic cod, polar cod, and Atlantic cod (NEAC). Chr denotes the chromosome number where an inversion (or multiple inversions) is detected in Arctic cod as well as the homologous chromosomes for the other two species. RC denotes the count of individuals in the rare cluster. Additionally, PC1 gives the explained variation for the first principal component, and lastly, the primary vs. adjusted region gives the location on the chromosome and validated size of the inversion region (in Mb). The regions/sizes are rounded to the nearest 100 Kb

Arctic cod				Polar cod				Atlantic cod (NEAC)			
Chr	RC	PC1	Primary/ Adjusted region (size)	Chr	RC	PC1	Primary/ Adjusted region (size)	Chr	RC	PC1	Primary/ Adjusted region (size)
1	2	43.7%	42–50/ 43.5–49.5 (6 Mb)	15^‡^	2	44.4%	13–18/ 13.7–17.5 (3.8 Mb)	18	2	53.4%	8–14/ 8–13.5 (5.5 Mb)
1	–	–	–	15^‡^	2	40.8%	6–8/ 6.6–7.6 (1 Mb)	–	–	–	–
3*	1	30.5%	3–8 (5 Mb)	18*	1	45.2%	4–6 (2 Mb)	19*	1	38.3%	7–10 (3 Mb)
6	4	56.3%	45–47 (2 Mb)	5	4	44.6%	8–12/ 9.1–10.9 (1.8 Mb)	17*	1	42.5%	1–5/ 1.9–3.8 (1.9 Mb)
7*	1	41.4%	20–23/ 20.9–22.2 (1.3 Mb)	3*	1	31.3%	51–54/ 52.7–53.8 (1.1 Mb)	15*	1	45.1%	18–20/ 18.6–19.8 (1.2 Mb)
7*	2	44.5%	30–32/ 30.2–31 (0.8 Mb)	16^§^	–	–	–	21*	2	37.9%	17–19/ 17.9–18.6 (0.7 Mb)
9*	2	23.15%	20–25 (5 Mb)	6	2	15%	20–23 (3 Mb)	4*	2	15.3%	22–27 (5 Mb)
10	2	42%	14–23 (9 Mb)	7	2	29.8%	8–15/ 8–16.3 (8.3 Mb)	12	2	38.7%	1–6 (5 Mb)
11	3	26.5%	10–24 (14 Mb)	8	3	26.4%	4–15 (11 Mb)	7	3	25.4%	14–29/ 14.8–28.2 (13.4 Mb)
13	3	29.5%	1–7/ start–6.7 (6.7 Mb)	14^‡^	3	17.7%	start–1 (1 Mb)	5	3	25.7%	1–4 (3 Mb)
13	–	–	–	14^‡^	3	13.9%	9–13/ 10.2–11.4 (1.2 Mb)	–	–	–	–
14	3	42%	23–end/ 23.6–end (3.2 Mb)	10	3	48.2%	21–end (2.9 Mb)	10	3	43.2%	23–27/ 23.7–27.2 (3.5 Mb)
15*	1	42.4%	7–12 (5 Mb)	12*	1	27.2%	start–3 (3 Mb)	2*	1	31.7%	16–20 (4 Mb)

^*^Putative inversions

^‡^Inversions split in two

^§^Inversion(s) not detected

The large number of inversions detected in Arctic cod is comparable with the higher number of inversions detected in polar cod, where in total 20 inversions are detected [28]. Both species reside in freezing water temperatures, and thus it is speculated that this high number is linked to cold water adaptations [27].

### Reference bias in inversion detection coupled to interspecific chromosomal reshufflings and translocations

**Fig. 6:**
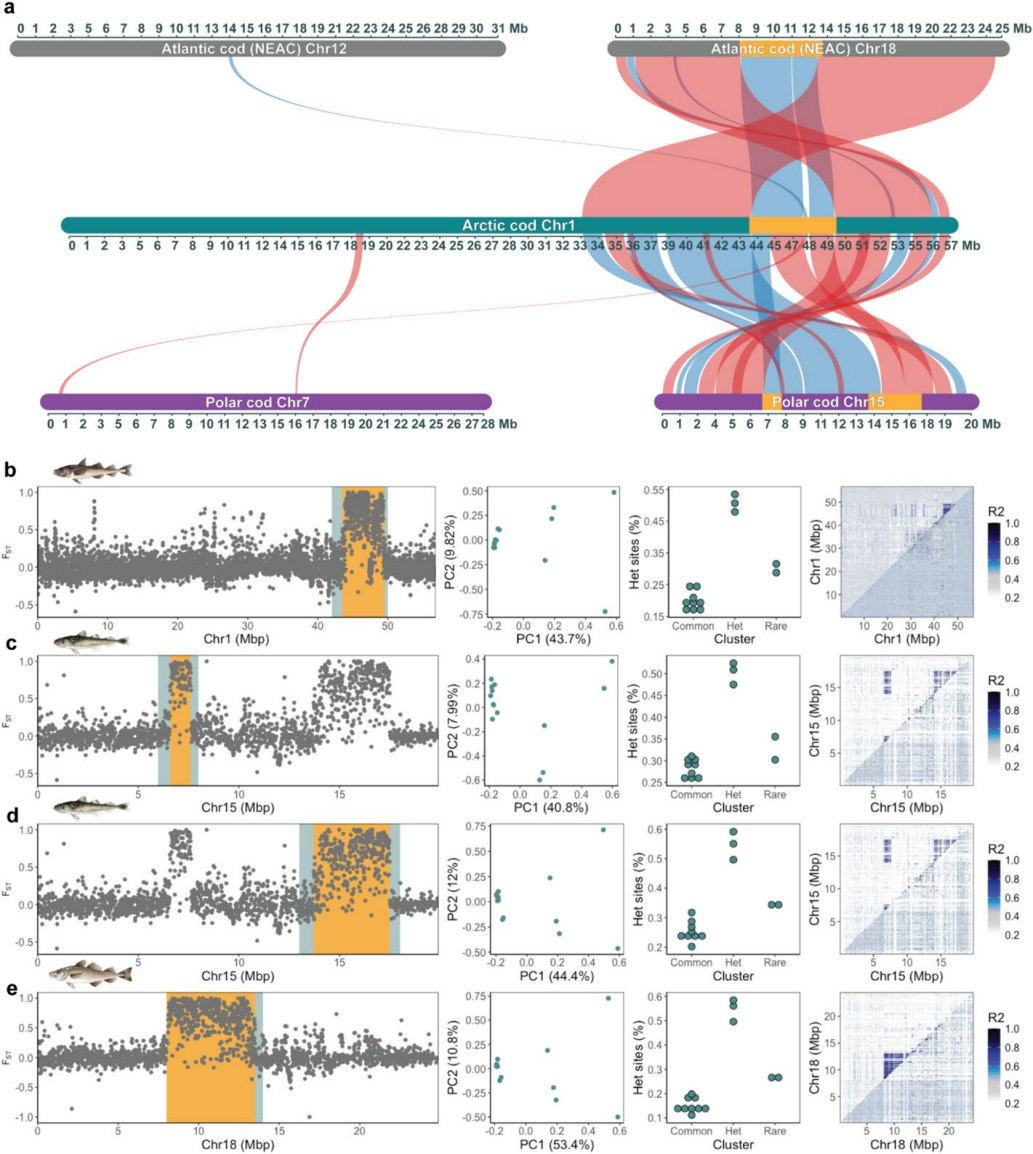
The bias detected for the inversion on the fused chromosome 1 in Arctic cod using Arctic cod, polar cod, and NEAC as reference genomes, respectively. **a** Synvisio plot illustrating the structural rearrangements occurring between the three species’ homologous chromosomes (identified by McScanX); where blue indicates same orientation, while red indicates reverse orientation. The panels, in **b**, **c**, **d** and **e** for the respective references used, display the pairwise F_ST_ between the rare and common clusters along chromosomes, PCA plots of the inversion region where the second PCA displays heterozygous sites (%) on the y-axis, and lastly the pairwise LD plots where the top triangle includes all samples, and the lower triangle includes only the individuals within the common type. The inversion region used for the first identification steps (PCA and MDS plots) is marked in blue/green, while the orange denotes the inversion region defined after the final validation steps, and the one used for subsequent analyses, as outlined in Figure S8. More specifically, **b** denotes the inversion detected when using Arctic cod as a reference, while **c** and **d** denote the inversion(s) detected when using polar cod as a reference, i.e., the inversion is split into two parts, but displays patterns of high LD. **e** The inversion detected when using NEAC as a reference. Here, the inversion was successfully captured, however, a smaller part was translocated to chromosome 12 in NEAC. Likewise, a smaller part of the detected inversion region was also translocated to Chromosome 7 in polar cod

**Fig. 7:**
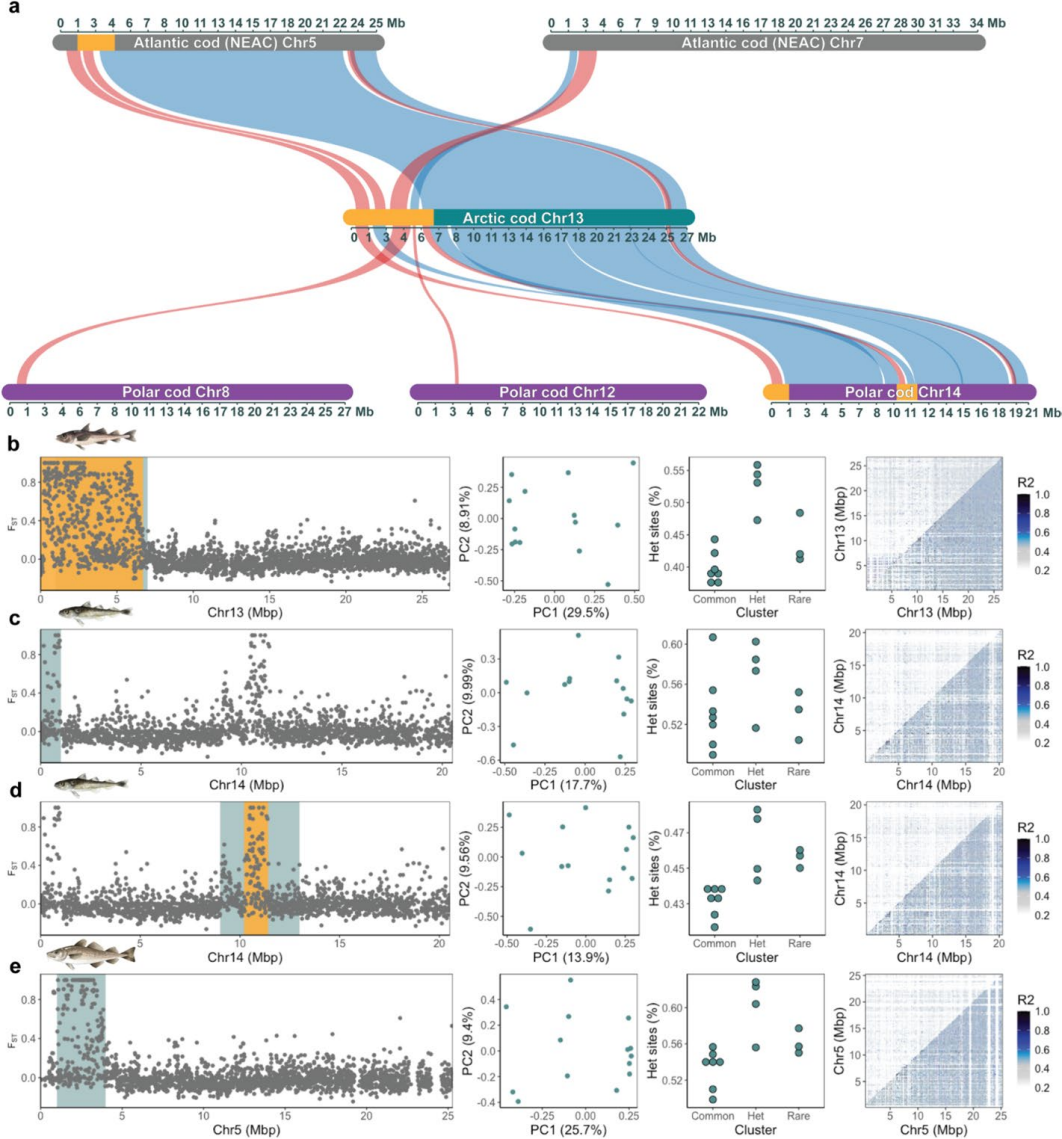
The bias detected for the inversion on chromosome 13 in Arctic cod using Arctic cod, polar cod, and NEAC as reference genomes, respectively. **a** Synvisio plot illustrating the structural rearrangements between the three species’ homologous chromosomes (identified by McScanX); where blue indicates the same orientation, while red indicates the reverse orientation. The panels, in **b**, **c**, **d** and **e** for the respective references used, display the pairwise F_ST_ between the rare and common clusters along chromosomes, PCA plots of the inversion region where the second PCA displays heterozygous sites (%) on the y-axis, and lastly the pairwise LD plots where the top triangle includes all samples, and the lower triangle includes only the individuals within the common type. The inversion region used for the first identification steps (PCA and MDS plots) is marked in blue/green, while the orange denotes the inversion region defined after the final validation steps, and the region used for subsequent analyses, as outlined in Figure S8. Here, multiple structural rearrangements/translocations between the species obscure the inversion signal. **b** The inversion detected when using Arctic cod as a reference, while **c** and **d** the inversion detected when using polar cod as a reference. Here the inversion(s) detected display low or no LD patterns, and thus, are seemingly called as two separate/independent inversion regions. Moreover, the heterozygosity signal is weaker in **c**, and none of the LD plots capture the inversion when using polar cod as reference. **e** The inversion detected when using Atlantic cod (NEAC) as a reference exhibits the expected heterozygosity distribution while the LD signal is weak

By taking advantage of the *intraspecific* datasets, we uncovered that the precision, in terms of size and location, of the inversion scoring became problematic when using a heterospecific reference (Table 1). In detail, when employing NEAC as the reference, all of the six fully validated inversions were confirmed as well as the five putative inversions (Additional file 5, Figure S21–S31). Even though all the inversions were detected, the majority of the inversions identified were found to be of a smaller size and with an incorrect chromosomal positioning compared with the corresponding inversions detected using Arctic cod as the reference. This discrepancy in the inversion scoring is likely coupled to larger species-specific genomic rearrangements and translocations between the focal species used in this study (as observed in Figs. 6 and 7) and described in detail in Hoff et al. [27]. For some of the inversions, however, a partly overlapping positioning was detected when inspecting the homologous chromosomes that enharbour the inversions, i.e., the inversions on chromosome 4, 7, 10, and 19 in NEAC vs. chromosome 9, 11, 14, and 3 in Arctic cod (Table 1).

When applying the assumed more closely related polar cod as the reference, all of the inversions were detected except one of the putative inversions (Table 1; Additional file 5, Figure S32–S44), i.e., the second inversion on chromosome 7 in Arctic cod (corresponding to chromosome 16 in polar cod; Additional file 5, Figure S32). Also here, the majority of the inversions identified were found to be of a smaller size and with incorrect chromosomal positioning compared with the corresponding inversions detected using Arctic cod (or the NEAC) as reference. Moreover, for the inversion detected on chromosome 1 in Arctic cod, the inversion appeared as two separate but linked inversions when using polar cod as the reference. This incorrect definition of the inversion is a result of species-specific chromosomal rearrangements between polar cod and Arctic cod (Fig. 6c and d; Figures S33 and S34). Similarly, inaccurate identification due to intraspecific chromosomal translocations (between all three species) is seen for the region harboring the inversion on chromosome 13 in Arctic cod (Fig. 7). When employing the polar cod genome as the reference, we find that the inversion is split into two separate inversions, with no clear LD signals, as well as a less clear heterozygosity distribution (Fig. 7c and d; Additional file 5, Figure S41 and S42). For the same region using NEAC as reference (Fig. 7e; Additional file 5, Figure S29), we capture the expected heterozygosity distribution; however, only weak LD signals were detected. Adding to the complexity of inversion detection when utilizing a more distantly related reference, the putative inversion on chromosome 3 (Additional File 5, Figure S18) in Arctic cod showed a much clearer LD signal when either of the heterospecific references was used, i.e., NEAC (Additional file 5, Figure S22) or polar cod (Additional file 5, Figure S35).

It could also be noted that a majority of the inversions, both when using the heterospecific as well as the species-specific reference, were defined with a larger size using lostruct/MDS plots alone (see Table 1; and Figs. 6 and 7; Additional file 5, Figure S9–S44), while all got generally smaller when adding the signals of F_ST_ and LD. Additionally, as mentioned above, all (except one) of the detected inversions when using the heterospecific reference were considerably smaller in size than the validated size using Arctic cod as a reference. The inconsistency in size detection could potentially be associated with the highly variable genomic regions often found in the breakpoint regions of an inversion [48, 49], and the sequence divergence of those regions between the species—as indicated with the reduced/fragmented mapping coverage in repetitive regions when using a heterospecific reference (Additional file 4, Figure S6)—combined with the fact that several of the inversions detected in Arctic cod have been found to partially overlap with inversions detected in both polar cod and Atlantic cod [27]. In addition to multiple species-specific chromosomal fusions and translocations, Hoff et al. [27] documented a high number of partly overlapping chromosomal inversions when comparing the two cold-water adapted species (i.e., Arctic cod and polar cod) and the more temperate Atlantic cod ecotypes. These partly overlapping genomic signatures are likely to interfere with the detection of the true size/signal of an inversion. For example, in addition to divergence in the repeat landscape of these regions, there are numerous reports on how inversions impact the recombination landscape both inside and outside of such high LD regions [50, 51]. Thus, caution should be taken when using a heterospecific reference for detections of inversions, especially if the inversion landscape is not yet fully evaluated in the focal species of the study systems.

### Gene content within the detected inversion regions using different reference genomes

**Fig. 8:**
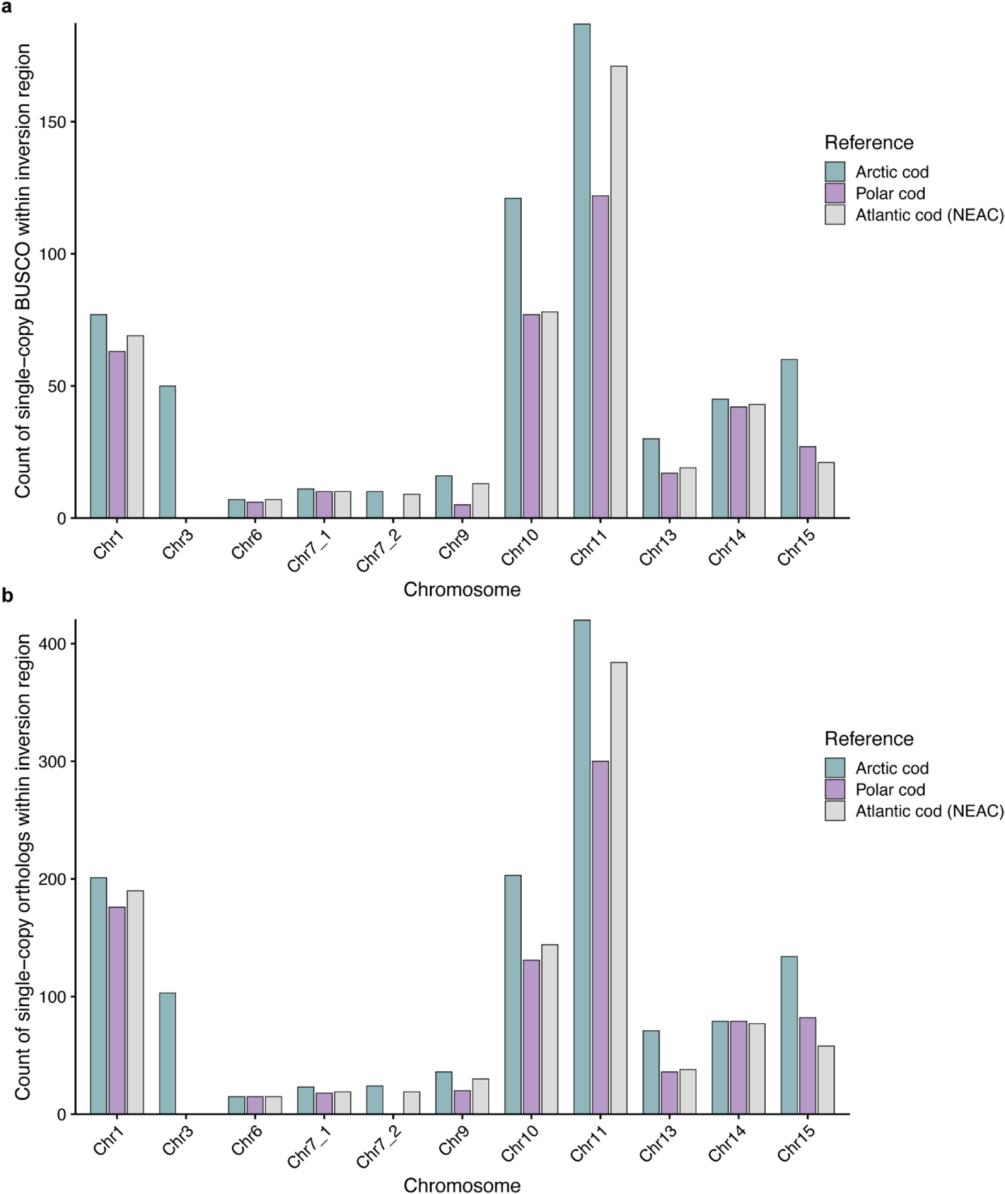
Gene content within inversion regions when using different references. **a** Single-copy BUSCOs and **b** single-copy genes identified using OrthoFinder retained within inversion regions when using the three different reference genomes

When conducting a conservative inspection of the gene content within the detected inversions—based on presence/absence of single-copy genes across all three reference genomes located within the inversion regions in the Arctic cod reference—we detected some degree of gene loss (a smaller number of genes) when using the heterospecific references (Fig. 8) vs. when using the species-specific reference. Since we restricted our analysis to single-copy orthologs, this problem could become more apparent if we were to compare all genes within the inversion regions across the three references. Taken together, our results clearly demonstrate that using a heterospecific reference for inversion detection—as well as the functional interpretation of these regions based on gene content analysis—could potentially affect our understanding of the functional implications of genomic rearrangements.

## Conclusions

Combined, our findings strongly indicate that caution must be exercised when using a heterospecific reference genome for mapping, variant calling, and subsequent population genetic estimates, and maybe more importantly, detection and characterization of larger structural inversions can be problematic. For population genetic measurements, our findings are concordant with previous studies, where both the quality of the reference used as well as degree of genomic divergence between the focal species and the reference have been shown to impact the number and quality of single nucleotide polymorphisms (SNPs) called due to i) a lower degree of mappability and thus, losing informative genetic variation, ii) potential misalignments which could lead to e.g., a bias towards higher degree of heterozygosity and more noisy datasets, where in-depth analyses on e.g., demographic history and detection of signals of selection are highly likely to be erroneous/inflated by this type of reference bias [10–14, 47]. Specifically, the general population genetic statistics in terms of heterozygosity, runs of homozygosity, and genetic diversity, are all metrics that are often used within conservation genomics as measurements for the health situation of a species and/or populations, by estimating the standing genetic variation and thus, their adaptive capacity [4, 10, 12, 52]. Our study shows that some of these metrics are seemingly more sensitive, such as heterozygosity levels, D_XY,_ and π. While the F_ST_ estimates are more robust, at least for detecting differentiation between species. However, it could be that F_ST_ estimates may be impacted if looking into differentiation within a species, i.e., between populations and/or ecotypes. Thus, based on this, caution should be taken when using a heterospecific reference for estimation of population specific measurements for conservation assessments.

Most importantly, we discovered that the use of reference impacted the detection and characterization of chromosomal inversions. Important information on size, position, and linkage between regions can easily be lost due to species-specific genomic rearrangements and smaller translocations [27]. For instance, using the assumed more closely related species—the polar cod—as the reference for the detection of inversions in Arctic cod resulted in the detection of several inversions that were defined as two inversions instead of one continuous larger inversion, as well as one inversion that was not discovered at all. This mismatch in detection of inversions is highly linked to the larger genomic reshufflings that have occurred after Arctic cod and polar cod branched off from their common codfish ancestor ~ 4 million years ago [24]. Moreover, a majority of the inversions detected were smaller than when using the focal species as the reference, suggesting that the breakpoint regions are not fully characterized when using a non-conspecific reference. The mismatch in the precision of the inversion detection is likely impacted by the fact that the focal species in our study harbor species-specific but overlapping polymorphic inversions with Arctic cod [21, 22, 27]. We speculate that highly variable breakpoint regions [48, 53] could lead to a higher degree of misalignments in these regions. Taken together, detection and functional inference of chromosomal inversions when using a non-conspecific reference should be handled with care. Especially since the breakpoint regions—where important genes under selection tend to be positioned [27, 54–57]—as well as other regions due to species-specific translocations seem to be lost in the scoring of the inversions. Furthermore, the number and interlinking of inversions may also be incomplete.

## Methods

### Sample acquisition and sequencing

The collection of Arctic cod (*N* = 14, Additional file 2, Table S1) used in this study was obtained via the TUNU-cruises (UiT, The Arctic University of Norway) and from other international collaborators, including *N* = 11 individuals from Northeast Greenland (Tyroler and Besselfjord) and *N* = 2 individuals from Canada (Davis Strait), as well as one specimen collected in the Barents Sea (Fig. 1b). The collection of polar cod (*N* = 14, Additional file 2, Table S1) is a subset from a larger dataset [28] from the northern Barents Sea (Fig. 1b).

All samples used in this study were collected in a responsible manner in connection with research surveys (as part of larger hauls for stock assessments). The fish were humanely sacrificed before sampling in accordance with the guidelines set by national and international animal welfare laws (e.g., https://norecopa.no), and thus no specific legislation was needed.

DNA isolation for Arctic cod was done by following the QIAGEN DNeasy Blood & Tissue kit protocol. DNA concentration measurement, library preparation, and sequencing were performed by the Norwegian Sequencing Centre. See Additional file 6, Sequencing Report for more information.

### Study design

**Fig. 9:**
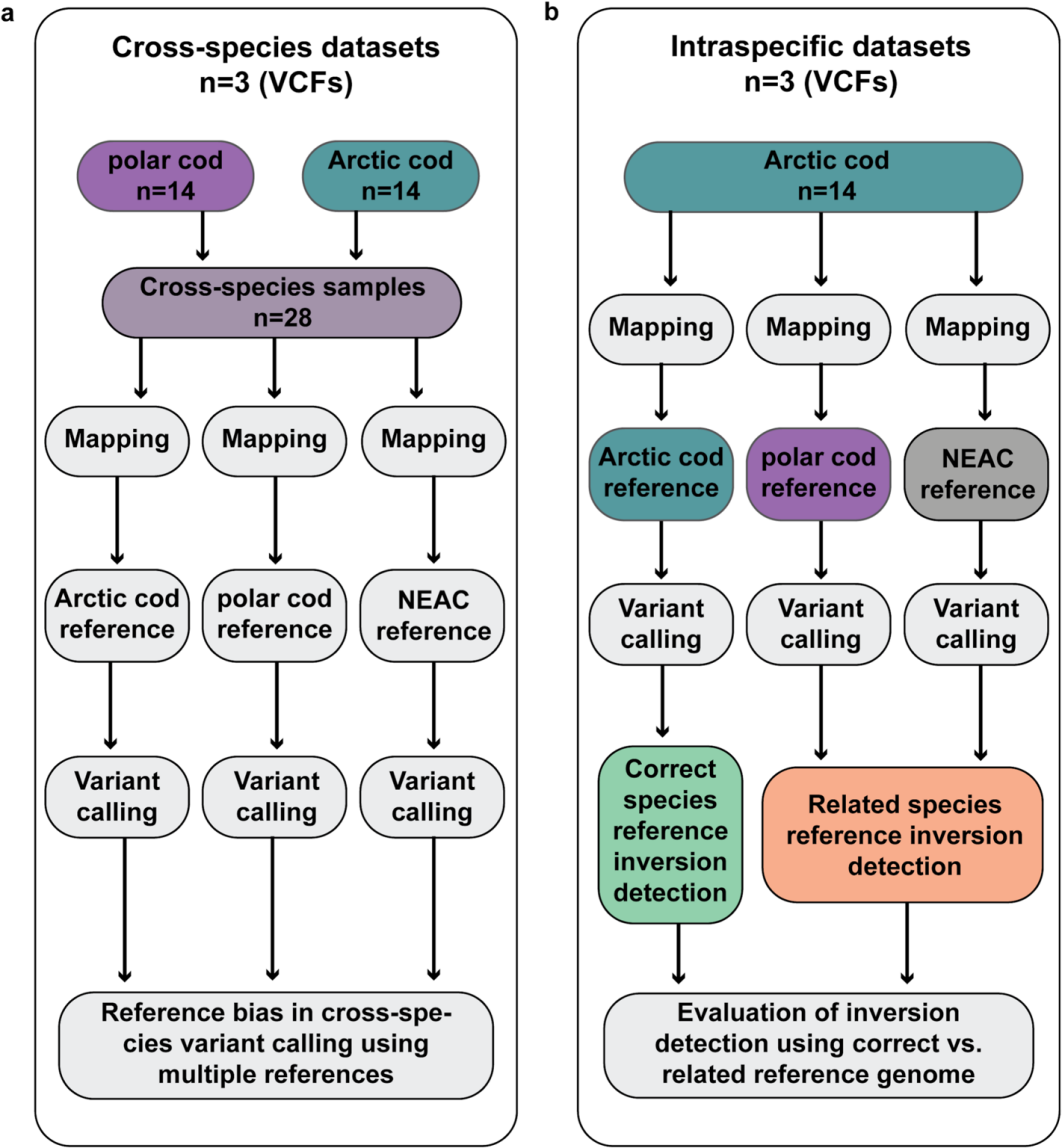
Flowchart of the study design and generation of the cross-species and intraspecific datasets. **a** For the generation of the three cross-species VCFs, we used samples of Arctic cod (*N* = 14) and polar cod (*N* = 14). Each sample was individually mapped against three different reference genomes: Arctic cod, polar cod, and Atlantic cod (NEAC). After mapping, the samples were grouped based on the reference genome they were mapped against. This approach was employed to assess the extent of reference bias in cross-species variant calling when using different reference genomes. **b** To investigate the impact of reference bias on inversion detection, we generated three intraspecific datasets focusing on Arctic cod samples (*N* = 14). These samples were mapped against the same three reference genomes used for the cross-species VCFs. In this analysis, the Arctic cod reference was considered the accurate benchmark for detecting inversions. To evaluate the influence of reference choice on inversion detection, the detected inversions were then compared to those identified when using a related species’ reference genome

The whole genome sequencing data were used to generate three *cross-species* datasets where data from both Arctic cod (*N* = 14) and polar cod (*N* = 14) were included (Fig. 9a), as well as three *intraspecific* datasets where we focused on the Arctic cod samples (Fig. 9b). Both sample collections (i.e., *cross-species* and *intraspecific*) were mapped against the reference genomes of either i) Arctic cod [27], ii) polar cod [27], or iii) the genome of the migratory ecotype of Atlantic cod, i.e., the Northeast Arctic cod (NEAC) [29], with the main purpose to assess the choice of reference on mapping statistics and thus, the subsequent variant calling. Additionally, population genetic measures, such as heterozygosity, nucleotide diversity (π), as well as genetic differentiation (F_ST_) and genetic divergence (D_XY_), were estimated to assess the influence of reference genome choice in a *cross-species* context. Moreover, we utilized the *intraspecific* datasets to assess the precision in the detection of chromosomal inversions within Arctic cod. This was conducted by comparing the degree of overlap between the inversions detected when using either the Arctic cod (i.e., the benchmark) vs. the polar cod or the NEAC genome as a reference.

To address whether the assembly quality could affect mapping statistics as well as the subsequent analyses, i.e., coupled to poorer mapping in high repetitive regions, we included two additional reference genomes for some of the analyses. For more information see Additional file 4. The two additional genomes included were an Oxford Nanopore draft genome assembly of polar cod (v2; fBorSai1.1.draft.fa.gz) as part of the EBP-Nor initiative (https://www.ebpnor.org/english/), as well as the newly released genome assembly of the coastal cod ecotype assembled using a combination of PacBio (CLR), Hi-C and 10X sequencing data (Hoff et al. [27]).

### Mapping and variant calling

For all the subsequent analyses, we started by trimming Illumina paired-end (PE) reads using Trimmomatic v0.39 [58] with default settings. Mapping to the different references was done using the Burrows-Wheeler Alignment Tool v0.7.17 [59] (BWA-MEM algorithm) with default settings. Alignment files for each sample were merged and sorted using SAMtools v1.9 [60]. Duplicated reads were marked using MarkDuplicates v2.22.1 [61]. See Additional file 4 for more information on the mapping analyses when using the different quality genome assemblies.

Furthermore, to obtain the six primary datasets, i.e., the three VCFs for the *cross-species* analysis and the three VCFs focusing on the *intraspecific* analysis, variant calling was performed using the Genome Analysis Toolkit (GATK) v4.2.0.0 [62]. For this, each mapped sample was individually called into GVCFs using HaplotypeCaller. GVCFs for individual samples were then combined into the six different VCFs, as described above in the experimental design, and imported into a GenomicsDataBase using GenomicsDBImport. Joint genotyping was performed using the GenotypeGVCFs tool to produce final VCFs. Single nucleotide polymorphisms (SNPs) were extracted and downsampled to 100,000 SNPs using SelectVariants to make diagnostic plots for filter parameter evaluation. Filtering was done by following the GATK hard-filtering recommendations and manually inspecting the diagnostic plots as suggested in https://speciationgenomics.github.io/. After the initial round of filtering, we used VCFtools v0.1.16 [63] to retain only biallelic sites (see Additional file 2, Table S2 and S3 for filtering parameters and Table S4 for the number of SNPs after filtering). Lastly, in-depth inspection of the datasets generated was conducted using PLINK v1.9 [64] and VCFtools v0.1.16 for detection of potential data biases (for more information see Additional file 1). A summary of the workflow is shown in Fig. 9.

### Evaluation of population structure, mapping, and variant calling based on reference used

We analyzed read depth distributions of mapped reads for Arctic cod and polar cod samples against the three references using mosdepth v0.2.4 [65] in fast mode, with a window size of 500 bp, while the proportion of heterozygous sites per sample was evaluated using VCFtools v0.1.16. The population genetic structure between and within the two species, i.e., both for the *cross-species* and the *intraspecific* datasets, was investigated by performing PCA using PLINK v1.9 and visualized using R v4.0.3.

For an evaluation of the genetic diversity within the *intraspecific* datasets, we carried out demographic inference and estimated female effective population size (N_e_) for Arctic cod using BEAST v2.6.7 [66] under the Bayesian skyline model [67]. The analysis was conducted on two separate datasets. The first was conducted by solely using the Arctic cod samples included in the present study (*N* = 14) and the second by including an addition of *N* = 20 samples of Arctic cod sourced from NCBI, resulting in a total of *N* = 33 samples (see Additional file 2, Table S5; Additional file 3 for more details). Additionally, for the *cross-species* datasets, π, F_ST_ [68], and D_XY_ between Arctic cod and polar cod were estimated using pixy v1.2.6 [69], applying a window size of 10,000 bp. The window-based estimates of average π were plotted using both regular scale and log scale.

### Detection of chromosomal inversions in Arctic cod

For the *intraspecific datasets* (i.e., the three Arctic cod VCFs mapped to the three different reference genomes), detection and validation of potential chromosomal inversions followed by identification of the inversion boundaries were performed using a complementary set of approaches. The workflow is illustrated in Additional file 5, Figure S8. For the detection and validation of inversions, we first used a PCA-based approach following Huang et al. [17]. This approach involved quantifying genetic variation within each of the chromosomes using the R package lostruct in windows of 50 SNPs [70]. In short, when conducting PCAs of an inversion region, three clusters will appear, i.e., representing the two homokaryotypes (of each arrangement) and the heterokaryotypes, where the heterokaryotypes are expected to cluster between the two homokaryotype clusters [71]. Thus, lostruct plots were manually checked for regions along chromosomes where PCAs for the MDS corners displayed three distinct clusters. Second, after detecting potential inversion regions, VCFtools v0.1.16 was used to extract the regions harboring the inversion signal and calculate the heterozygosity for each sample, which was then used to calculate new PCAs using PLINK v1.9 within the identified regions. In the cases where the PCA displayed an inversion signal, clusters were assigned to either homokaryotypes, i.e., those with most individuals (hom common) or those with the fewest individuals (hom rare), or as heterokaryotypes, i.e., those belonging to the group clustering in the center with the highest heterozygosity measurements (het). Due to the low sample size, the heterozygosity distribution could not be plotted using conventional boxplots; instead, we used a binning strategy implemented in the ggplot2 function geom_dotplot.

The third step in the validation process was the inspection of the F_ST_ and D_XY_ estimates calculated using pixy v1.2.6 in windows of 10,000 bp between the rare and common clusters along chromosomes to assess patterns of genetic differentiation and divergence outside and inside potential inversion regions. As a fourth step of the validation process, patterns of LD were investigated for the chromosomes that displayed potential signals of inversions. The expectation for chromosomes harboring inversions is that regions within the inversion will show high LD among all samples (when both homokaryotypes are present) but not among samples with the same inversion orientation [17]. As the calculation of LD in a pairwise fashion for whole chromosomes produces millions of data points, the number of SNPs had to be down-sampled. PLINK v1.9 was used to remove sites with more than 0.01% missing data, and SNPs were randomly thinned down to 10% of the original count. After thinning, PLINK v1.9 was used to calculate LD in a pairwise fashion within the chromosome of interest. Due to the high number of data points still left, the R package scattermore was used to produce the LD plots [72].

Lastly, for the detected inversions—both those that fulfilled the criteria defined by our inversion detection protocol (see above) and those that we defined as putative inversions, i.e., regions displaying more or less the same patterns as the other inversions, but with either weaker LD signals, less clear heterozygosity levels defined for the heterokaryotypes, and/or only 2 or fewer individuals in the rare cluster—the boundaries were readjusted and more precisely defined if needed. This was mainly for those inversions where discrepancies in the first definition were observed vs. what was indicated when inspecting the signals of F_ST_, LD, and MSD plots combined. The readjusted inversion regions were denoted on the Figures with an orange rectangle (also exemplified in our workflow illustrated in Additional file 5, Figure S8).

### Synteny between the three references

Syntenic block analyses were performed for the identification of the homologous chromosomes as well as chromosomal translocations and rearrangements between the focal species used in this study, i.e., Arctic cod, polar cod, and Atlantic cod (NEAC) using McScanX [73]. The McScanX analyses were performed using the developers’ recommendations, first doing reciprocal all-by-all BLAST search between all proteins of the annotated gene for all six species. The hits were afterwards anchored to a bed-like file made from the annotation GFF file, with information about gene names and the genomic locations of each gene. Synteny blocks were then built using McScanX with default settings, i.e., gene gap size set to 10. The results of the synteny analyses were subsequently visualized using the SynVisio interactive webpage [74], with the displacement of the syntenic maps between the genome assemblies.

### Gene content within the detected inversion regions using different reference genomes

To examine the impact of using a heterospecific reference on the functional interpretation of inversions, which might be obscured by misidentification of the breakpoint regions, larger genomic rearrangements/translocations, and/or indels of genes within the inversion regions, we analyzed gene content within the defined inversion regions using two different gene sets. First, we utilized results from a BUSCO genome completeness analysis using COMPLEASM v0.2.7 [75] with the Actinopterygii odb12 database [76]. Second, we ran OrthoFinder v2.5.5 [77] using the three annotations for the three reference genomes. To be conservative and ensure we were comparing orthologs rather than paralogs, we filtered each dataset for genes that were single-copy across all three reference genomes located within the inversion regions in the Arctic cod reference and then assessed these genes for presence/absence in the corresponding Arctic cod inversion regions identified in the polar cod and Atlantic cod reference genomes.

## Supplementary Information

Additional file 1: Sample statistics vs PCA plots of Arctic cod using three references [63, 64, 81].

Additional file 2: Supplementary Tables [28, 82, 83].

Additional file 3: Mitochondrial demographic inference [27, 60, 66, 67, 82–91].

Additional file 4: Mappability [27, 43–46, 75, 76, 84, 92].

Additional file 5: Workflow for detection of chromosomal inversions and inversion detection using the different reference genomes.

Additional file 6: Supplementary Sequencing Report.

## Data Availability

Raw sequences from the Arctic cod dataset have been deposited in the European Nucleotide Archive (ENA) at EMBL-EBI with the following accession nr: PRJEB88972 [78]. Moreover, the genome assemblies used as references in this study can be found under the following ENA accession nr: GCA_964260595.1 for *Arctogadus glacialis* (arcglaasm) [79], GCA_964260565.1 for *Boreogadus saida* (borsaiasm) [79], GCA_902167405.1 for *Gadus morhua* ‘NEAC’ (gadMor3.0) [29] and GCA_964260575.1 for *Gadus morhua* ‘NCC’ (gadmorasm) [79] The newly generated Oxford Nanopore draft genome of *Boreogadus saida* (fBorSai1.1.draft.fa.gz) is available on zenodo 10.5281/zenodo.16882388 [80].
