# Supplementary material for: Reference genome bias in light of species-specific chromosomal reorganization and translocations
Source: Genome Biol. 2025 Oct 15;26:355. doi: 10.1186/s13059-025-03761-w (PMC12523119; doi:10.1186/s13059-025-03761-w)
Supplement: Supplementary file 1 — Additional file 1: Sample statistics vs PCA plots of Arctic cod using three references [63, 64, 81]. [file 13059_2025_3761_MOESM1_ESM.docx]

### **Additional file 1**

### **Sample statistics vs PCA plots of Arctic cod using three references**

We used PLINK v1.9 [[64]](https://www.zotero.org/google-docs/?Y0ys7n) to perform a Principal Component Analysis (PCA) on the Arctic cod samples for all three *intraspecific* VCFs. To evaluate potential data biases, we also calculated mean depth, missing sites, and heterozygosity among these samples using VCFtools v0.1.16 [[63]](https://www.zotero.org/google-docs/?tRhJvH). These statistics were then plotted alongside the first two principal components to assess statistical bias of samples [[81]](https://www.zotero.org/google-docs/?NWMu9o) that might affect the distribution of the samples used.

**
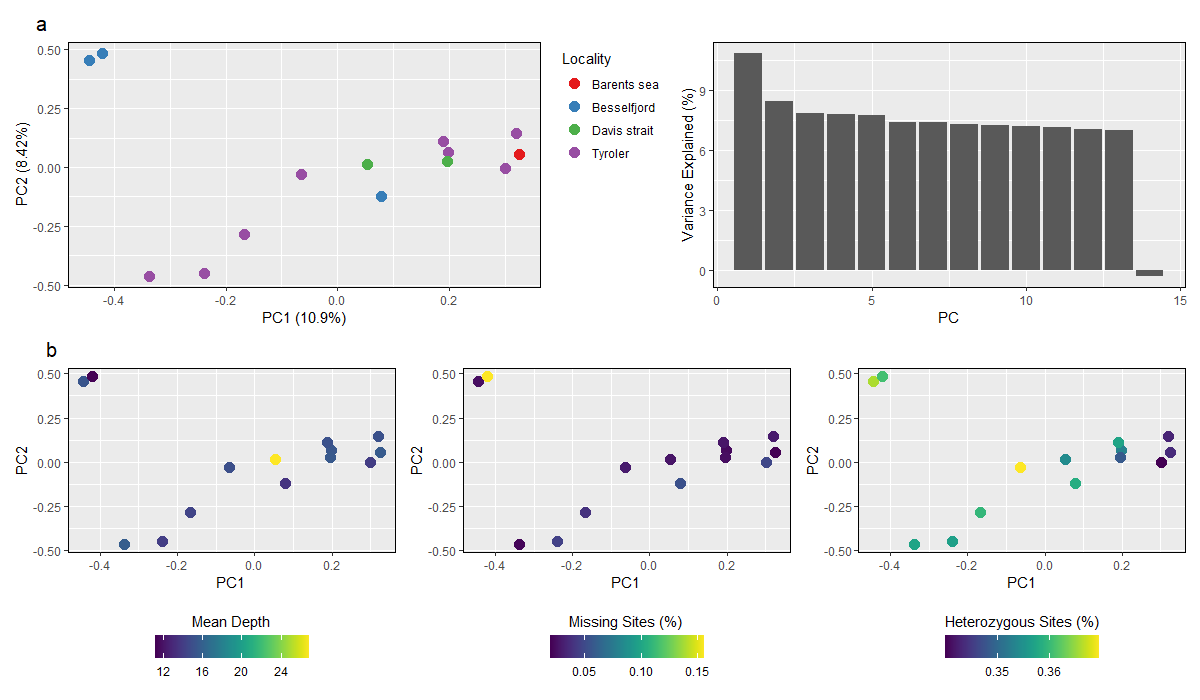
**

**Figure S1.** PCA (PC1 – PC2) versus sample statistics of Arctic cod samples using Arctic cod as reference. a) PCA and corresponding eigenvalues of Arctic cod samples colored by locality. b) PCA against mean depth (left), proportion of missing sites (middle) and proportion of heterozygous sites (right).

**
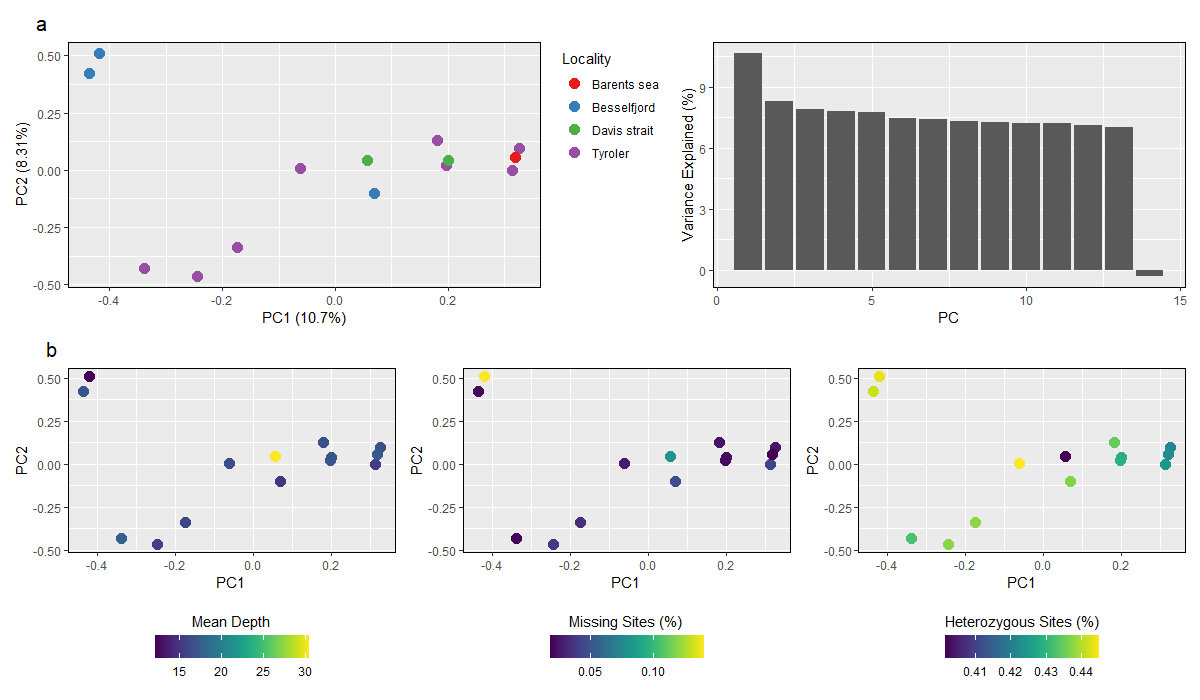
**

**Figure S2.** PCA (PC1 – PC2) versus sample statistics of Arctic cod samples using polar cod as reference. a) PCA and corresponding eigenvalues of Arctic cod samples colored by locality. b) PCA against mean depth (left), proportion of missing sites (middle), and proportion of heterozygous sites (right).


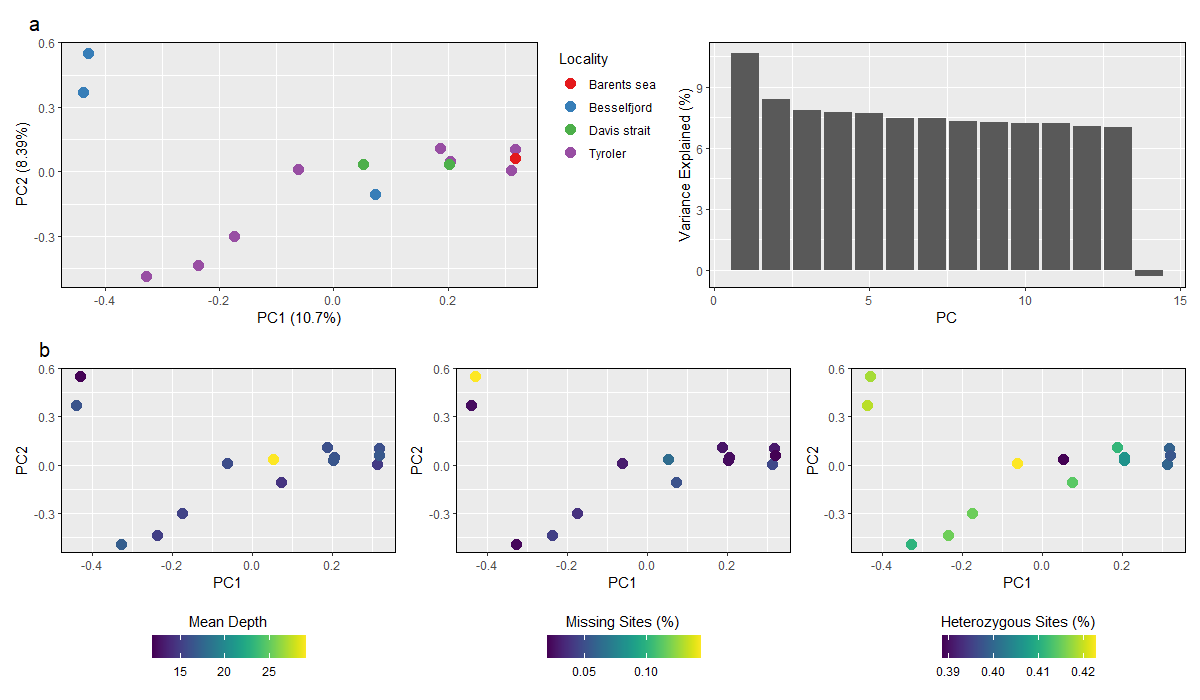


**Figure S3.** PCA (PC1 – PC2) versus sample statistics of Arctic cod samples when using NEAC as reference. a) PCA and corresponding eigenvalues of Arctic cod samples colored by locality. b) PCA against mean depth (left), proportion of missing sites (middle) and proportion of heterozygous sites (right).

### **References**

[63. Danecek P, Auton A, Abecasis G, Albers CA, Banks E, DePristo MA, et al. The variant call format and VCFtools. Bioinformatics. 2011;27:2156–8.](https://www.zotero.org/google-docs/?TiPyPW)

[64. Purcell S, Neale B, Todd-Brown K, Thomas L, Ferreira MAR, Bender D, et al.](https://www.zotero.org/google-docs/?TiPyPW) PLINK: A tool set for whole-genome association and population-based linkage analyses. Am. J. Hum. Genet. 2007;81:559–75.

[81. Yi X, Latch EK. Nonrandom missing data can bias Principal Component Analysis inference of population genetic structure. Mol. Ecol. Res. 2022;22:602–11.](https://www.zotero.org/google-docs/?TiPyPW)
