# Supplementary material for: Reference genome bias in light of species-specific chromosomal reorganization and translocations
Source: Genome Biol. 2025 Oct 15;26:355. doi: 10.1186/s13059-025-03761-w (PMC12523119; doi:10.1186/s13059-025-03761-w)
Supplement: Supplementary file 2 — Additional file 2: Supplementary Tables [28, 82, 83]. [file 13059_2025_3761_MOESM2_ESM.docx]

### **Additional file 2**

**Supplementary Tables**

**Table S1.** Samples of Arctic cod and polar cod [[28]](https://www.zotero.org/google-docs/?SISMZG) used in this study, including sample ID, length (millimeters), weight (grams), sex, maturity (Mat), trawling locality, DNA extraction protocol, and sample concentration after DNA extraction.

| Sample ID | Length (mm) | Weight (g) | Sex | Mat | Trawling locality | Protocol | Sample concentration (ng/µl) |
| --- | --- | --- | --- | --- | --- | --- | --- |
| 01-Ag13010 | 206 | 72 | M | 1 | Greenland, Tyroler | Qiagen | 85.05 |
| 02-Ag13014 | 189 | 49.9 | F | 1 | Greenland, Tyroler | Qiagen | 77.13 |
| 03-Ag13019 | 266 | 133 | F | 2 | Greenland, Tyroler | Qiagen | 153.00 |
| 04-Ag13020 | 203 | 56.6 | F | 1 | Greenland, Tyroler | Qiagen | 80.41 |
| 05-Ag13021 | 231 | 82.9 | F | 1 | Greenland, Tyroler | Qiagen | 86.16 |
| 06-Ag13024 | 210 | 67.4 | F | 1 | Greenland, Tyroler | Qiagen | 72.53 |
| 07-Ag13026 | 182 | 45 | F | 0 | Greenland, Tyroler | Qiagen | 69.21 |
| 08-Ag13027 | 204 | 70.2 | M | 1 | Greenland, Tyroler | Qiagen | 64.76 |
| 09-Agl17001 | 203 | 59.55 | F | 1 | Greenland, Besselfjord | Qiagen | 68.43 |
| 10-Agl17002 | 181 | 39.6 | M | 1 | Greenland, Besselfjord | Qiagen | 46.72 |
| 11-Agl17003 | 155 | 21.5 | * | * | Greenland, Besselfjord | Qiagen | 112.46 |
| 12-Ag-ds-0A08045-2 | * | * | * | * | Davis Strait | Qiagen | 7.45 |
| 13-Ag1-hyb-aen-2018 | 131 | 16 | F | 1 | Barents Sea | Qiagen | 239.74 |
| SAMEA4028798+ | * | * | * | * | Davis Strait | ENA: ERR1473882, ERR1473883 | * |
| 04-polcod59 | 15,5 | 24 | F | 2 | Barents Sea | Omega | 14.83 |
| 171-polcod1 | 15 | 22.75 | M | 2 | Barents Sea | Omega | 209.20 |
| 172-polcod2 | 14,1 | 19.35 | M | 2 | Barents Sea | Omega | 138.52 |
| 174-polcod4 | 13,6 | 18.75 | F | 2 | Barents Sea | Omega | 24.15 |
| 178-polcod8 | 15,7 | 24.45 | M | 4 | Barents Sea | Omega | 62.59 |
| 185-polcod20 | 17 | 34 | M | 2 | Barents Sea | Omega | 19.55 |
| 187-polcod37 | 14,3 | 22 | M | 2 | Barents Sea | Omega | 35.96 |
| 188-polcod45 | 17,5 | 35 | F | 3 | Barents Sea | Omega | 16.34 |
| 189-polcod49 | 13,3 | 18 | F | 2 | Barents Sea | Omega | 11.56 |
| 190-polcod50 | 16 | 35 | F | 2 | Barents Sea | Omega | 36.29 |
| 192-polcod52 | 19,5 | 55 | F | 3 | Barents Sea | Omega | 62.70 |
| 228-polcod62 | 18,4 | 49 | M | 4 | Barents Sea | Omega | 131.39 |
| 235-polcod74 | 18,8 | 48 | M | 3 | Barents Sea | Omega | 15.49 |
| 287-polcod31 | 18 | 39 | F | 3 | Barents Sea | Omega | 64.50 |

*****No information available. +Accessed from ENA: ERR1473882, ERR1473883.Protocols; Omega: Omega Mag-Bind M6399, Qiagen: QIAGEN Dneasy Blood & Tissue kit.

**Table S2.** Filter parameters applied using GATK VariantFiltration. Values indicate at which threshold SNPs were removed.

| VCF | QD | FS | SOR | MQ | MQRankSum | ReadPosRankSum |
| --- | --- | --- | --- | --- | --- | --- |
| Intraspecific Arctic cod | < 2 | > 60 | > 3 | < 50 | < -4 | < -5 |
| Cross-species | < 8 | > 60 | > 3 | < 50 | < -10 | < -5 |

**Table S3.** Filtering parameters applied using VCFtools. The parameter max missing is on a scale between 0 and 1 where 1 means no missing data is allowed.

| VCF | maf | mac | minQ | max-missing | meanDP (min / max) | DP (min / max) |
| --- | --- | --- | --- | --- | --- | --- |
| Intraspecific Arctic cod | 0.075 | 2 | 30 | 0.9 | 8 / 50 | 8 / 50 |
| Cross-species | No filtering | 2 | 30 | 0.9 | 8 / 50 | 8 / 50 |

**Table S4.** SNP counts for the different VCFs created in this study.

| VCFs | Number of SNPs | | |
| --- | --- | --- | --- |
|  | Arctic cod reference | Polar cod reference | NEAC reference |
| Intraspecific Arctic cod | 899623 | 661357 | 721148 |
| Cross-species | 298668 | 275094 | 275248 |

**Table S5.** Accession numbers and the related studies for the Arctic cod samples used in the mitochondrial demographic history analysis.

| Accession | Study |
| --- | --- |
| MH035564.1-MH035582.1 | Wilson et al. [82] |
| NC_010122.1 | Breines et al. [83] |

### **References**

[28. Hoff SNK, Maurstad MF, Le Moan A, Ravinet M, Pampoulie C, Vieweg I, et al. Population divergence manifested by genomic rearrangements in a keystone Arctic species with high gene flow. bioRxiv. 2024;2024.06.28.597535.](https://www.zotero.org/google-docs/?TiPyPW)

82. Wilson RE, Sage GK, Sonsthagen SA, Gravley MC, Menning DM, Talbot SL. Genomics of Arctic cod. OCS Study. Bureau of Ocean Energy Management. Report No.: BOEM 2017-066. 2017. https://pubs.er.usgs.gov/publication/70197204. Accessed 01.06.2022.

83. Breines R, Ursvik A, Nymark M, Johansen SD, Coucheron DH. Complete mitochondrial genome sequences of the Arctic Ocean codfishes *Arctogadus glacialis* and *Boreogadus saida* reveal oriL and tRNA gene duplications. Polar Biol. 2008;31:1245–52.
