# Supplementary material for: Reference genome bias in light of species-specific chromosomal reorganization and translocations
Source: Genome Biol. 2025 Oct 15;26:355. doi: 10.1186/s13059-025-03761-w (PMC12523119; doi:10.1186/s13059-025-03761-w)
Supplement: Supplementary file 3 — Additional file 3: Mitochondrial demographic inference [27, 60, 66, 67, 82–91]. [file 13059_2025_3761_MOESM3_ESM.docx]

### **Additional file 3**

### **Mitochondrial demographic inference**

Mitochondrial reads mapped in proper pairs against the Arctic cod [[27]](https://www.zotero.org/google-docs/?T4VZ4s) mitogenome for each sample were extracted and sorted by read name using SAMtools v1.9 [[60]](https://www.zotero.org/google-docs/?sNErwq). Bam files were converted back to FASTQ format using BEDTools v2.27.1 [[84]](https://www.zotero.org/google-docs/?7vsT9Y) bamtofastq tool. Assembly of mitogenomes was performed using MitoFinder v1.4.1 [[85]](https://www.zotero.org/google-docs/?VEuh8a) with default settings and the assembler MEGAHIT v1.0 [[86,87]](https://www.zotero.org/google-docs/?Hkpksg). MiTFi v0.1 [[88]](https://www.zotero.org/google-docs/?ca8F70) was used to annotate the mitochondrial tRNAs. Mitogenome annotation was performed using the available mitogenome reference for Arctic cod (NCBI RefSeq: NC_010122.1) as MitoFinder requires annotated mitogenomes in GenBank reference format. Annotated mitochondrial protein-coding genes (PCGs) from each individually MitoFinder assembled intraspecific level mitogenome of Arctic cod were extracted and aligned using MAFFT v7.453 [[89]](https://www.zotero.org/google-docs/?eq6EqW). To allow for all samples to be included, Cytochrome c oxidase subunit II (*COX2*) had to be removed as this gene was missing for one sample. Furthermore, the PCGs were manually corrected for reading frame before they were concatenated with PhyKIT v1.11.7 [[90]](https://www.zotero.org/google-docs/?Qik1Nw) create_concat to produce a supermatrix.

The female effective population size (N_e_) was estimated in BEAST v2.6.7 [[66]](https://www.zotero.org/google-docs/?nHjUYH) under the Bayesian skyline model [[67]](https://www.zotero.org/google-docs/?OYSK7a). The substitution model was inferred using bModelTest with the namedExtended list of models. The coalescent Bayesian skyline prior was applied under a strict clock with a rate of 1.14x10^-8^ substitution/site/year used, as reported for Atlantic cod [[91]](https://www.zotero.org/google-docs/?ROByEM). The analysis was performed using a chain length of 800,000,000, and sampling was done every 1,000 iterations with bPopSizes and bGroupSizes set to 5 dimensions. Tracer v1.7.2 was used to check convergence and to reconstruct the Bayesian skyline with default settings. (skyline variant = stepwise (constant), maximum time to root height = lower 95% HPD, root height = Treeheight, number of bins = 100).

Due to the low sample size of Arctic cod in the present study, the Bayesian skyline analysis was repeated a second round. Here, we included the available Arctic cod data from NCBI in addition to the samples from this study (N=14). The data sourced from NCBI included 19 Arctic cod samples from Wilson et al. [[82]](https://www.zotero.org/google-docs/?Ytt3Oa) and one Arctic cod sample from Breines et al. [[83]](https://www.zotero.org/google-docs/?xC7avl) (Additional file 2, Table S5). These mitogenomes were in partial condition. Therefore, to maximize sample count and PCG count, the individual with missing *COX2* from our study was removed, resulting in a total of N=33 samples and 10 PCGs.

Demographic inference based on the N=14 Arctic cod individuals (Figure S4a) indicated an increase in female effective population size (N_e_) around the last glacial maximum (LGM), as well as a slight diagonal increase around 40 Kya. When Arctic cod samples from NCBI were included (N=33, Figure S4b), the N_e_ increase was steepest around 40 Kya, with a slight diagonal increase after the LGM.

**
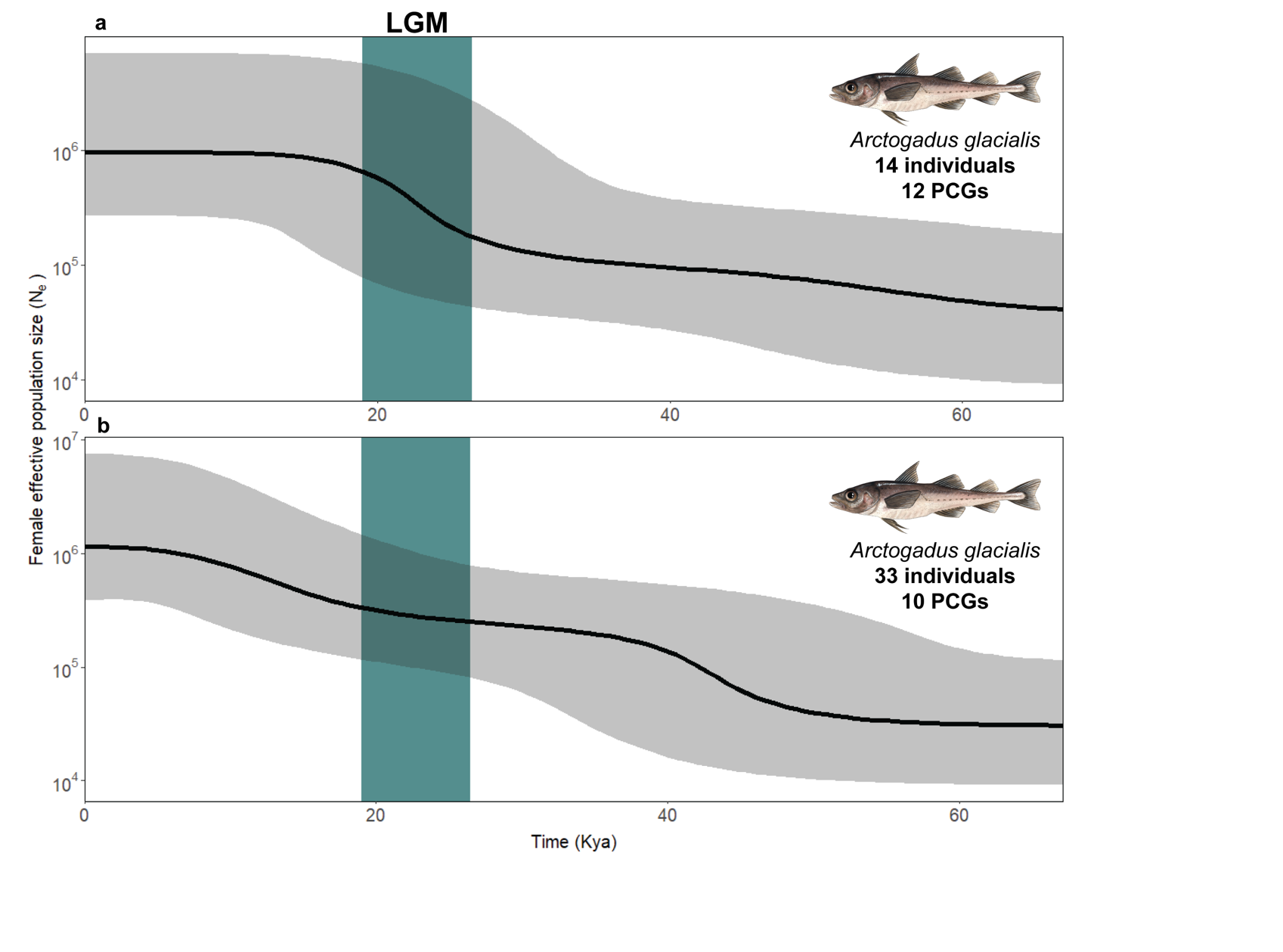
**

**Figure S4.** Inferred female effective population size (N_e_) for Arctic cod and mitochondrial demographic inference. a) Demographic history of Arctic cod using the 14 individuals from this study and 12 PCGs (*COX2* removed due to it being incomplete within one individual), b) Demographic history of Arctic cod including N=20 samples sourced from NCBI in addition to the (N=14) Arctic cod samples included in the present study, while excluding the individual with incomplete *COX2*. The blue bar indicates the last glacial maximum.

### **References**

27. Hoff SNK, Maurstad MF, Tørresen OK, Berg PR, Præbel K, Jakobsen KS, et al. Chromosomal fusions and large-scale inversions are key features for adaptation in Arctic codfish species. bioRxiv. 2024;2024.06.28.599280.

60[. Danecek P, Bonfield JK, Liddle J, Marshall J, Ohan V, Pollard MO, et al. Twelve years of SAMtools and BCFtools. GigaSci. 2021;10:giab008.](https://www.zotero.org/google-docs/?TiPyPW)

66. Bouckaert R, Vaughan TG, Barido-Sottani J, Duchêne S, Fourment M, Gavryushkina A, et al. BEAST 2.5: An advanced software platform for Bayesian evolutionary analysis. PLoS Comput. Biol. 2019;15:e1006650.

67. Drummond AJ, Rambaut A, Shapiro B, Pybus OG. Bayesian coalescent inference of past population dynamics from molecular sequences. Mol. Biol. Evol. 2005;22:1185–92.

82. Wilson RE, Sage GK, Sonsthagen SA, Gravley MC, Menning DM, Talbot SL. Genomics of Arctic cod. OCS Study. Bureau of Ocean Energy Management. Report No.: BOEM 2017-066. 2017. https://pubs.er.usgs.gov/publication/70197204. Accessed 01.06.2022.

83. Breines R, Ursvik A, Nymark M, Johansen SD, Coucheron DH. Complete mitochondrial genome sequences of the Arctic Ocean codfishes *Arctogadus glacialis* and *Boreogadus saida* reveal oriL and tRNA gene duplications. Polar Biol. 2008;31:1245–52.

[84. Quinlan AR, Hall IM. BEDTools: a flexible suite of utilities for comparing genomic features. Bioinformatics. 2010;26:841–2.](https://www.zotero.org/google-docs/?TiPyPW)

85. Allio R, Schomaker-Bastos A, Romiguier J, Prosdocimi F, Nabholz B, Delsuc F. MitoFinder: Efficient automated large-scale extraction of mitogenomic data in target enrichment phylogenomics. Mol. Ecol. Res. 2020;20:892–905.

[86. Li D, Luo R, Liu C-M, Leung C-M, Ting H-F, Sadakane K, et al. MEGAHIT v1.0: A fast and scalable metagenome assembler driven by advanced methodologies and community practices. Methods. 2016;102:3–11.](https://www.zotero.org/google-docs/?TiPyPW)

[87. Li D, Liu C-M, Luo R, Sadakane K, Lam T-W. MEGAHIT: an ultra-fast single-node solution for large and complex metagenomics assembly via succinct de Bruijn graph. Bioinformatics. 2015;31:1674–6.](https://www.zotero.org/google-docs/?TiPyPW)

88. Jühling F, Pütz J, Bernt M, Donath A, Middendorf M, Florentz C, et al. Improved systematic tRNA gene annotation allows new insights into the evolution of mitochondrial tRNA structures and into the mechanisms of mitochondrial genome rearrangements. Nucl. Aci. Res. 2012;40:2833–45.

89. Katoh K, Standley DM. MAFFT Multiple sequence alignment software version 7: Improvements in performance and usability. Mol. Biol. Evol. 2013;30:772–80.

[90. Steenwyk JL, Buida TJ III, Labella AL, Li Y, Shen X-X, Rokas A. PhyKIT: a broadly applicable UNIX shell toolkit for processing and analyzing phylogenomic data. Bioinformatics. 2021;37:2325–31.](https://www.zotero.org/google-docs/?TiPyPW)

91. Lait LA. A mitogenomic study of four at-risk marine fish species: Atlantic wolffish, spotted wolffish, northern wolffish, and Atlantic cod, with special emphasis on the waters off Newfoundland and Labrador. Memorial University of Newfoundland; 2016. https://research.library.mun.ca/12490/. Accessed 25.08.2022.
