# Supplementary material for: Reference genome bias in light of species-specific chromosomal reorganization and translocations
Source: Genome Biol. 2025 Oct 15;26:355. doi: 10.1186/s13059-025-03761-w (PMC12523119; doi:10.1186/s13059-025-03761-w)
Supplement: Supplementary file 4 — Additional file 4: Mappability [27, 43–46, 75, 76, 84, 92]. [file 13059_2025_3761_MOESM4_ESM.docx]

### **Additional file 4**

### **Mappability**

To address how assembly quality affected the mapping outcomes, we included two additional reference genomes, i.e., an Oxford Nanopore draft genome assembly of polar cod (v2; fBorSai1.1.draft.fa.gz) as part of the EBP-Nor initiative (https://www.ebpnor.org/english/)), as well as the newly released genome assembly of the coastal cod ecotype assembled using a combination of PacBio (CLR), Hi-C and 10x sequencing data (Hoff et al. [[27]](https://www.zotero.org/google-docs/?R093ad)).

We first assessed sequence similarity between all reference genomes using Mash distance estimation [[43]](https://www.zotero.org/google-docs/?jTBgPY) (Figure S5a). The two polar cod assemblies showed high degree of sequence similarity as well as the two Atlantic cod genomes, i.e., the migratory ecotype (NEAC) and the more stationary Norwegian coastal ecotype (NCC). The two latter were in fact, found to be most similar to each other. In contrast, the Arctic cod was found to be equally distant from both the Atlantic cod and polar cod. To evaluate assembly completeness, we calculated BUSCO scores using COMPLEASM v0.2.7 [[75]](https://www.zotero.org/google-docs/?RNE5zG) with the Actinopterygii odb12 [[76]](https://www.zotero.org/google-docs/?BNcaaT) dataset (Figure S5b). The newly sequenced polar cod genome achieved the highest completeness (98.52%), followed closely by the NCC, i.e. the coastal cod, (98.07%), indicating that these genomes were more complete compared to the other references.

We then analyzed mapping quality (MAPQ) in 10 kb windows across the primary chromosomes for each sample (Figure S5c) using bedtools bamtobed [[84]](https://www.zotero.org/google-docs/?qw50XL) and then bedtools map to calculate mean MAPQ scores within each genomic window and then averaged for each sample. SAMtools v1.14 flagstat was used on the bam files to generate the properly paired and primary mapped statistics (Figure S5d-e).

Given that codfish species are known for their relatively high repeat content [[44–46]](https://www.zotero.org/google-docs/?SkKUx2), we further investigated whether repetitive regions could drive mappability differences between references. For this, we calculated repeat density using RED [[92]](https://www.zotero.org/google-docs/?zxQpoi) in 10 kb windows and binned coverage by repeat content categories (0-20%, 20-40%, 40-60%, 60-80%, and 80-100%) and used bedtools coverage to calculate the fraction of bases covered by sequencing reads within each window (Figure S6).


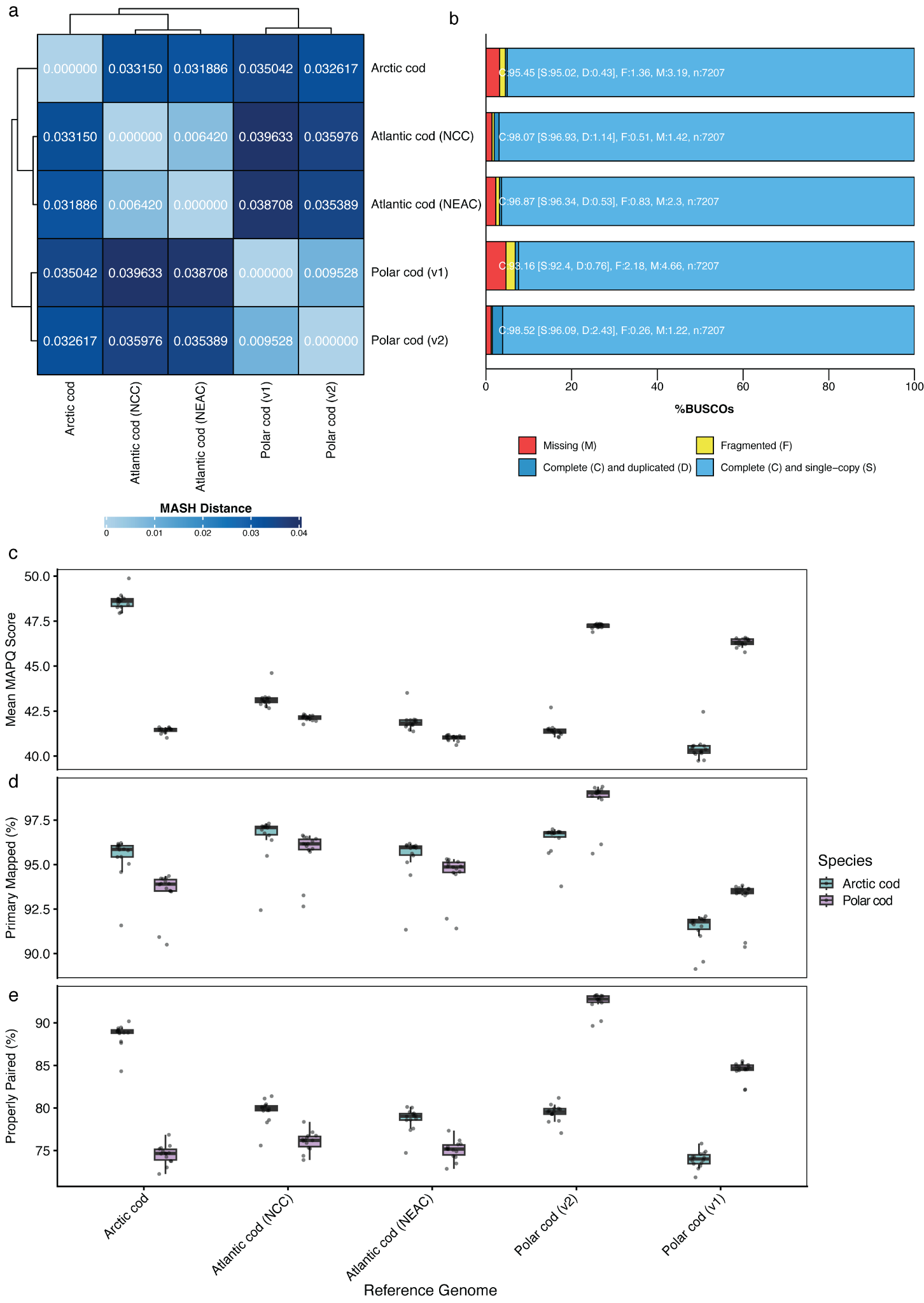


**Figure S5.** Genome distance, genome completeness, and mappability of population samples. a) Hierarchical clustering heatmap showing pairwise Mash distances between reference genomes: Arctic cod, Polar cod, Atlantic cod, coastal ecotype of Atlantic cod, and a newly sequenced Polar cod genome. Mash distances range from 0 (identical) to higher values (more divergent), with darker blue indicating greater genomic similarity. b) BUSCO completeness scores for each reference genome were calculated using compleasm with the Actinopterygii odb10 dataset, indicating assembly quality and gene content completeness. Mapping quality metrics for polar cod and Arctic cod samples based on the the reference genome used, including c) mean MAPQ scores (average mapping quality across genomic windows for primary chromosomes), d) primary mapped percentage (proportion of reads mapping as primary alignments), and e) properly paired percentage (fraction of paired-end reads mapping in proper orientation and insert-size). MAPQ scores were computed using bedtools to calculate mean mapping quality within genomic windows, then averaged per sample across primary chromosomes for each sample mapped against each reference.

**
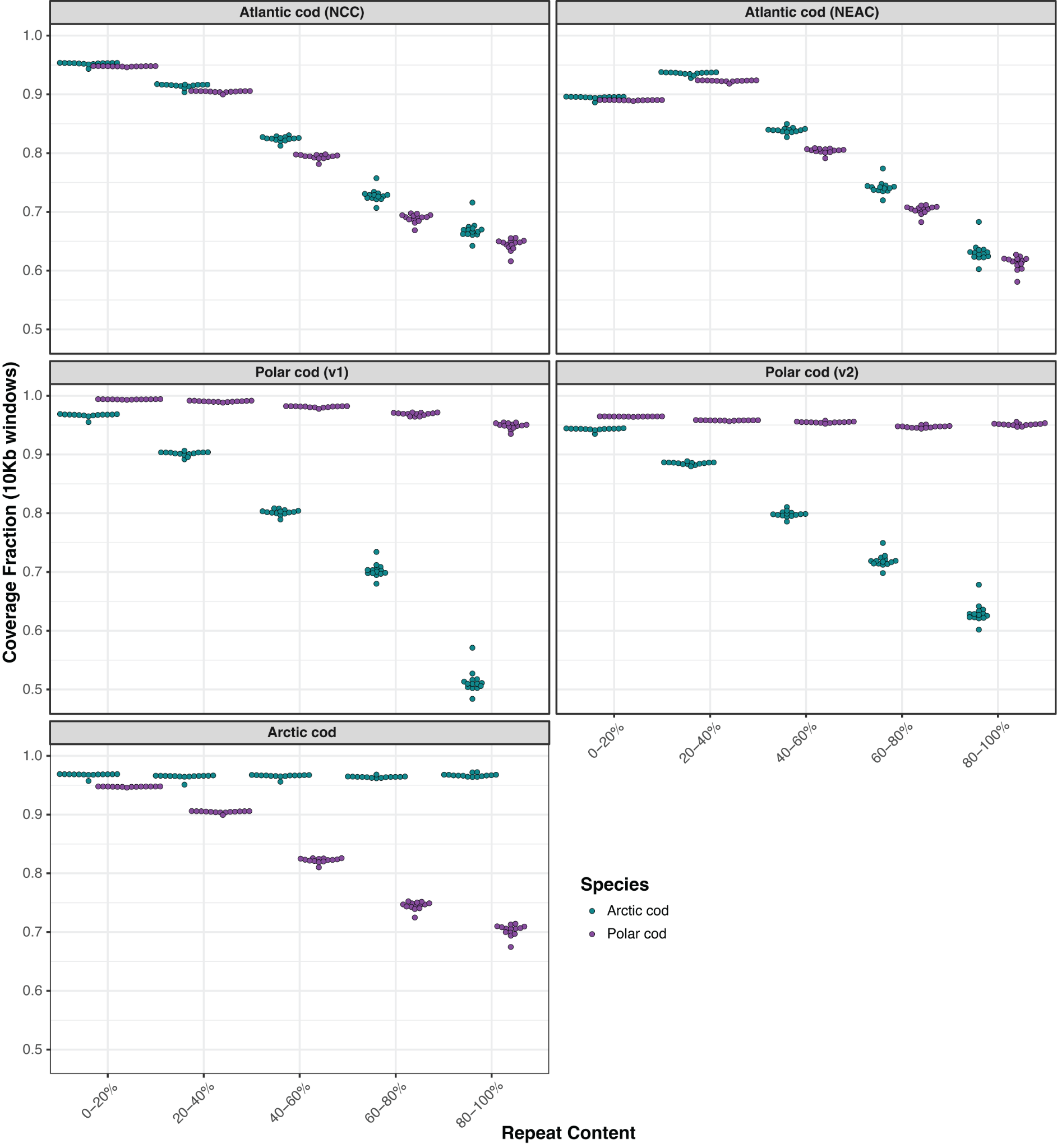
**

**Figure S6.** Coverage distribution across genomic regions with varying repeat content. Beeswarm plot showing the relationship between repeat density calculated using RED and sequencing coverage across reference genomes using bedtools coverage on 10 kb non-overlapping genomic windows. Analysis was restricted to primary chromosomes only. Genomic windows were categorized by repeat content percentage (0-20%, 20-40%, 40-60%, 60-80%, 80-100%), and coverage fraction was calculated as the proportion of bases covered by sequencing reads within each window. Each point represents the average coverage fraction for one sample within a specific repeat density category.


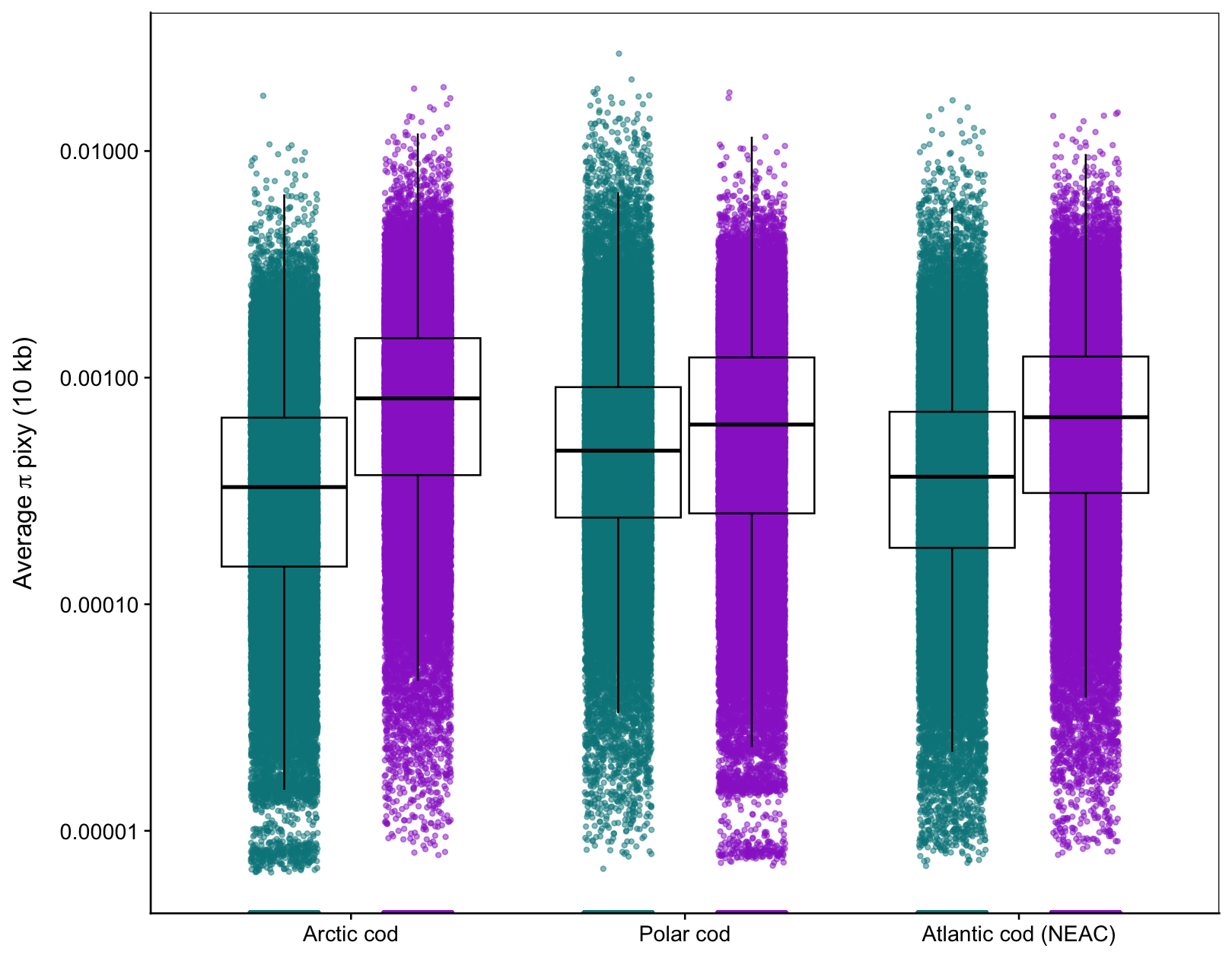


**Figure S7.** Average π values across genomic windows. Average nucleotide diversity (π) values were calculated in windows across three different reference genomes (Arctic cod, polar cod, and Atlantic cod) for Arctic cod and polar cod samples using Pixy. The panel is the same as Figure 3c but with the y-axis displayed on a logarithmic scale (log10).

### **References**

27. Hoff SNK, Maurstad MF, Tørresen OK, Berg PR, Præbel K, Jakobsen KS, et al. Chromosomal fusions and large-scale inversions are key features for adaptation in Arctic codfish species. bioRxiv. 2024;2024.06.28.599280.

[43. Ondov BD, Treangen TJ, Melsted P, Mallonee AB, Bergman NH, Koren S, et al. Mash: fast genome and metagenome distance estimation using MinHash. Genom. Biol. 2016;17:132.](https://www.zotero.org/google-docs/?TiPyPW)

44. Tørresen OK, Star B, Jentoft S, Reinar WB, Grove H, Miller JR, et al. An improved genome assembly uncovers prolific tandem repeats in Atlantic cod. BMC Genom. 2017;18:95.

45. Tørresen OK, Brieuc MSO, Solbakken MH, Sørhus E, Nederbragt AJ, Jakobsen KS, et al. Genomic architecture of haddock (*Melanogrammus aeglefinus*) shows expansions of innate immune genes and short tandem repeats. BMC Genom. 2018;19:240.

[46. Reinar WB, Tørresen OK, Nederbragt AJ, Matschiner M, Jentoft S, Jakobsen KS. Teleost genomic repeat landscapes in light of diversification rates and ecology. Mobile DNA. 2023;14:14.](https://www.zotero.org/google-docs/?TiPyPW)

[75. Huang N, Li H. compleasm: a faster and more accurate reimplementation of BUSCO. Bioinformatics. 2023;39:btad595.](https://www.zotero.org/google-docs/?TiPyPW)

[76. Tegenfeldt F, Kuznetsov D, Manni M, Berkeley M, Zdobnov EM, Kriventseva EV. OrthoDB and BUSCO update: annotation of orthologs with wider sampling of genomes. Nucl. Aci. Res. 2025;53:D516–22.](https://www.zotero.org/google-docs/?TiPyPW)

[84. Quinlan AR, Hall IM. BEDTools: a flexible suite of utilities for comparing genomic features. Bioinformatics. 2010;26:841–2.](https://www.zotero.org/google-docs/?TiPyPW)

92[. Girgis HZ. Red: an intelligent, rapid, accurate tool for detecting repeats de-novo on the genomic scale. BMC Bioinf. 2015;16:227.](https://www.zotero.org/google-docs/?TiPyPW)
