# Supplementary material for: Reference genome bias in light of species-specific chromosomal reorganization and translocations
Source: Genome Biol. 2025 Oct 15;26:355. doi: 10.1186/s13059-025-03761-w (PMC12523119; doi:10.1186/s13059-025-03761-w)
Supplement: Supplementary file 5 — Additional file 5: Workflow for detection of chromosomal inversions and inversion detection using the different reference genomes. [file 13059_2025_3761_MOESM5_ESM.docx]

### **Additional file 5**

### **Workflow for detection of chromosomal inversions**


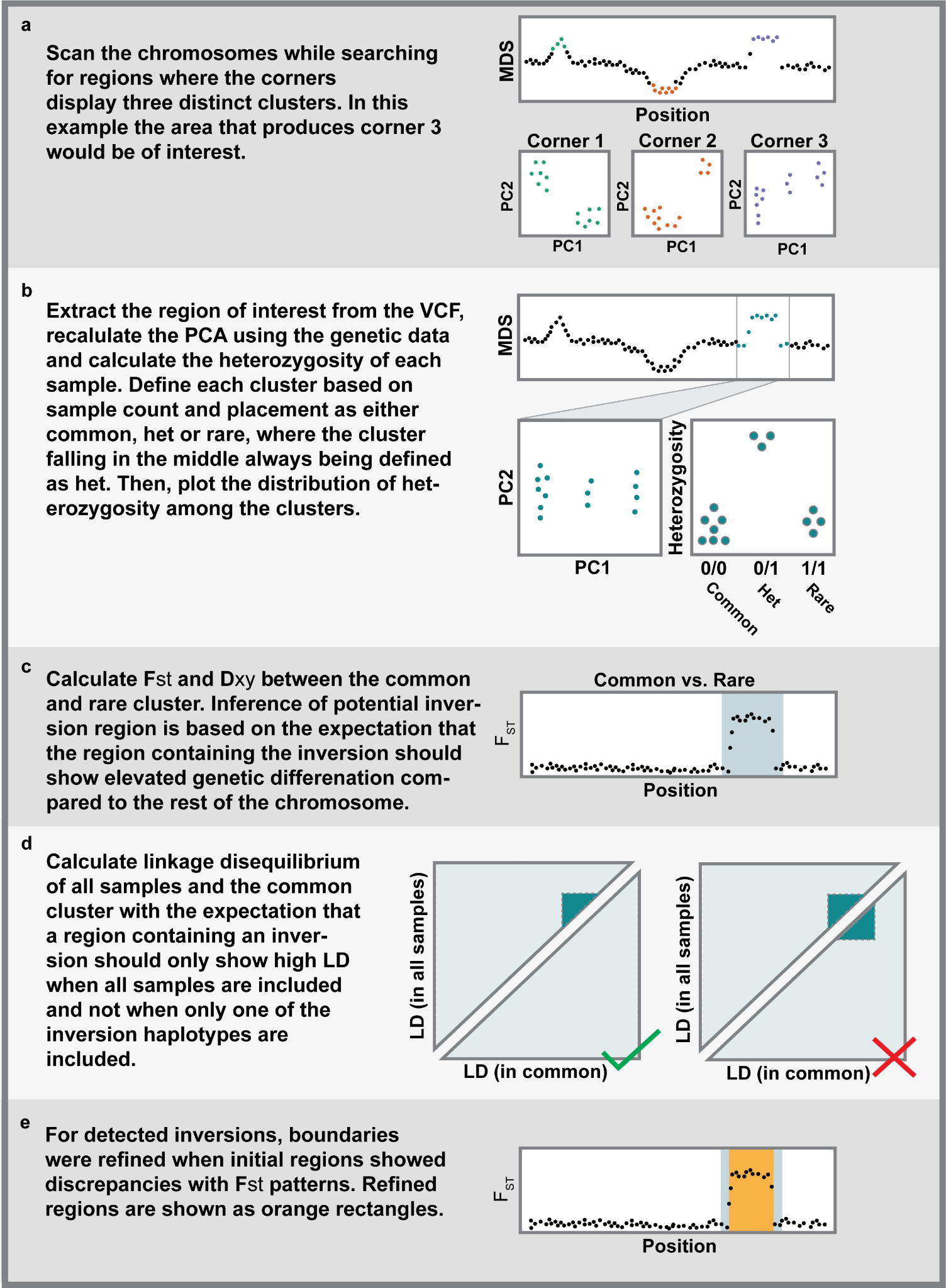


**Figure S8.** Illustration of the protocol used to detect chromosomal inversion Arctic cod. The procedure is followed stepwise from a-d. The inversion region used for the first identification steps (PCA and MDS plots) are marked in blue/green, while the validated size is given in orange. The size/boundaries of the inversions are rounded to the nearest 100 Kb.

### **Inversion detection plots using Arctic cod as reference genome**


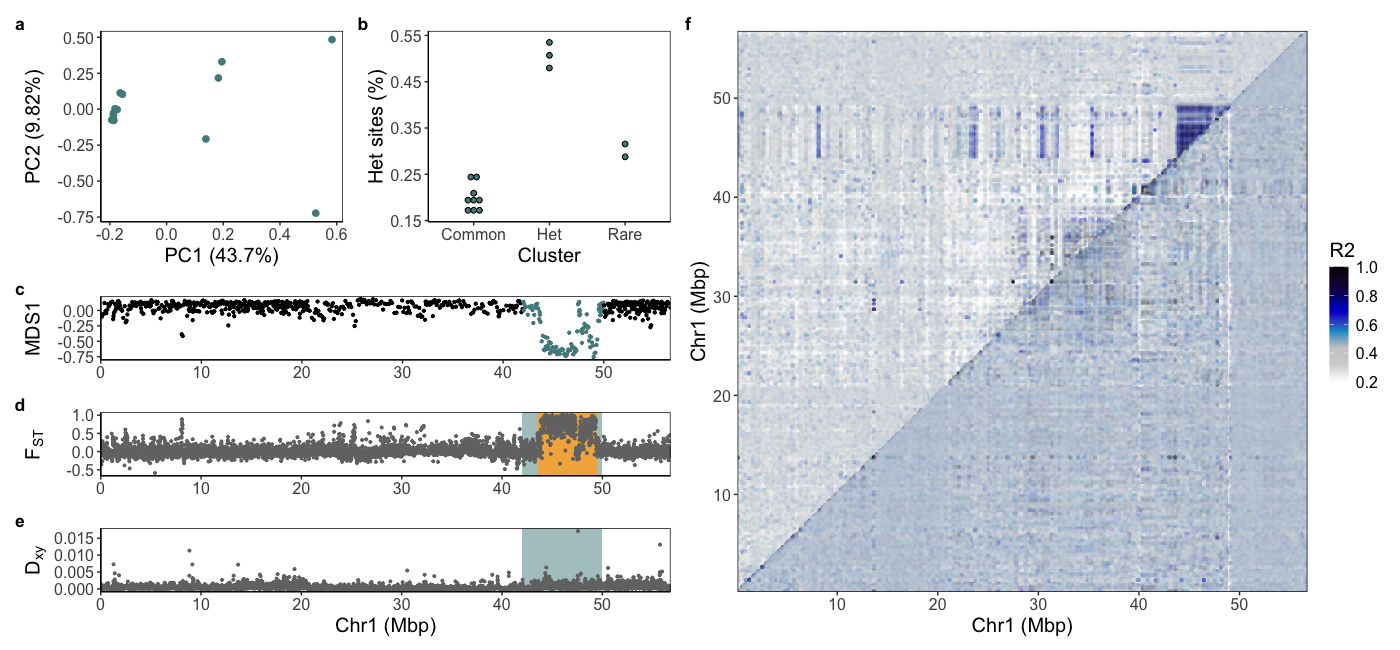


**Figure S9.** Inversion detected on chromosome 1 of Arctic cod using Arctic cod as reference genome. a) PCA for the inversion region identified using lostruct. b) Manually assigned cluster groups and % heterozygous sites given in bins for the clusters. c) MDS analysis produced by lostruct where the inversion region is highlighted. d) F_ST_ and e) D_XY_ calculated with pixy showing elevated values within the highlighted inversion region. f) pairwise linkage disequilibrium plot calculated using pixy where the top triangle includes all samples, and the lower triangle includes only the individuals within the common type.


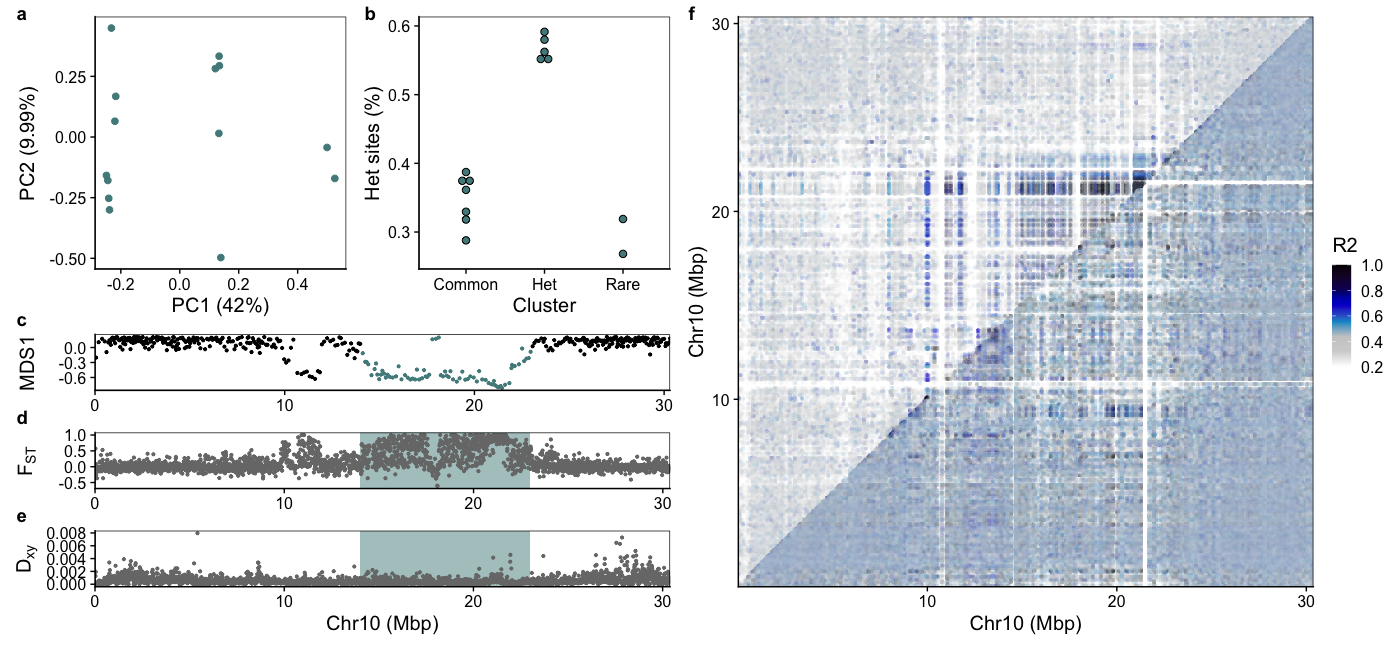


**Figure S10.** Inversion detected on chromosome 10 of Arctic cod using Arctic cod as reference genome. a) PCA for the inversion region identified using lostruct. b) Manually assigned cluster groups and % heterozygous sites given in bins for the clusters. c) MDS analysis produced by lostruct where the inversion region is highlighted. d) F_ST_ and e) D_XY_ calculated with pixy showing elevated values within the highlighted inversion region. f) pairwise linkage disequilibrium plot calculated using pixy where the top triangle includes all samples, and the lower triangle includes only the individuals within the common type.


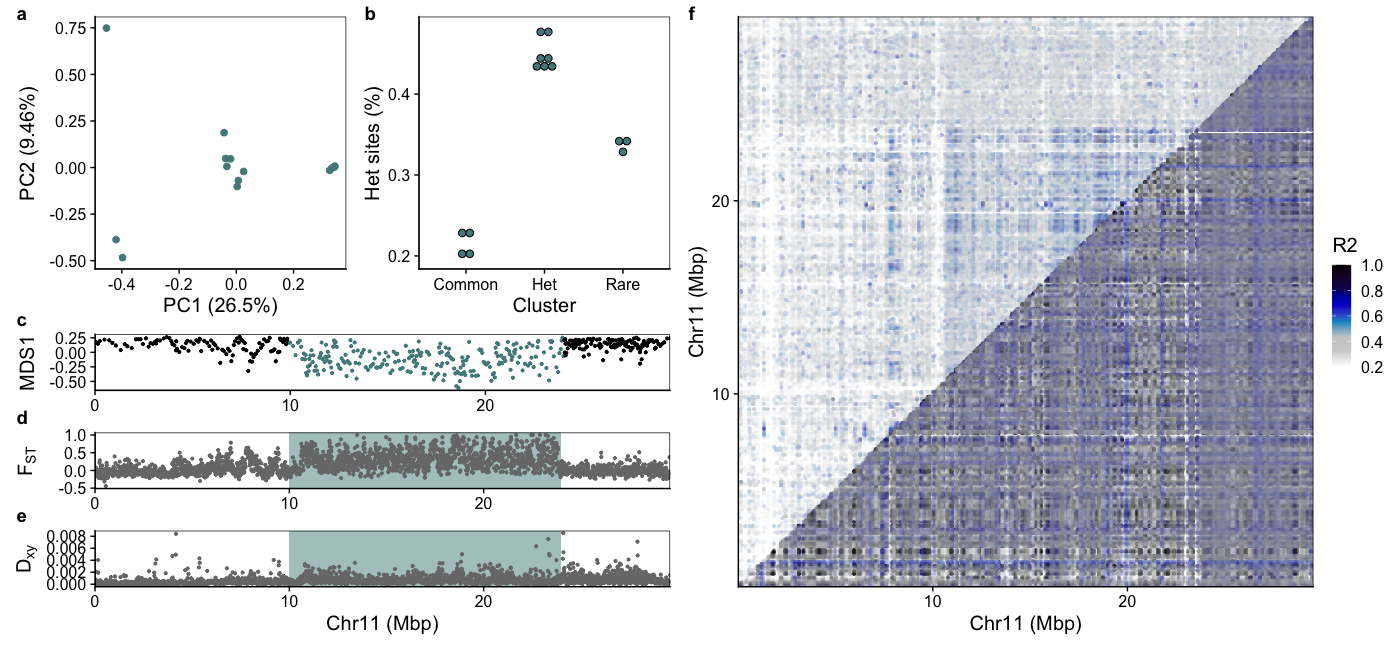


**Figure S11.** Inversion detected on chromosome 11 of Arctic cod using Arctic cod as reference genome. a) PCA for the inversion region identified using lostruct. b) Manually assigned cluster groups and % heterozygous sites given in bins for the clusters. c) MDS analysis produced by lostruct where the inversion region is highlighted. d) F_ST_ and e) D_XY_ calculated with pixy showing elevated values within the highlighted inversion region. f) pairwise linkage disequilibrium plot calculated using pixy where the top triangle includes all samples, and the lower triangle includes only the individuals within the common type.


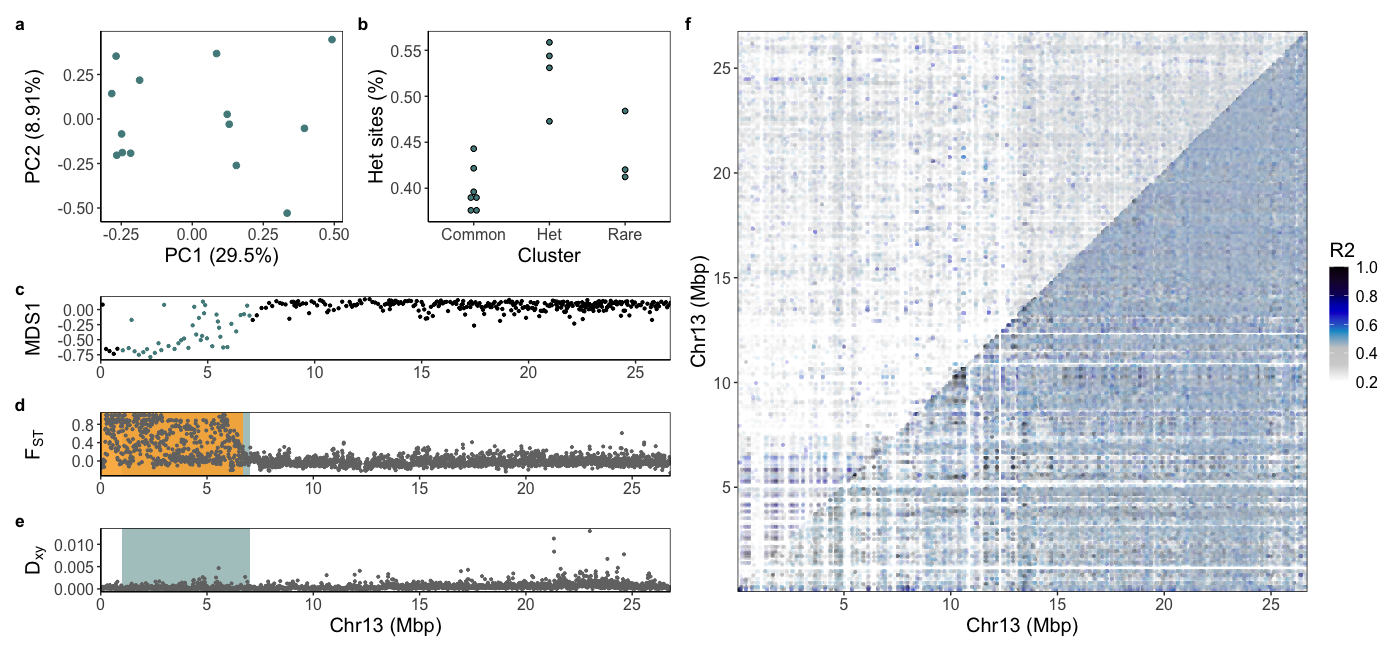


**Figure S12.** Inversion detected on chromosome 13 of Arctic cod using Arctic cod as reference genome. a) PCA for the inversion region identified using lostruct. b) Manually assigned cluster groups and % heterozygous sites given in bins for the clusters. c) MDS analysis produced by lostruct where the inversion region is highlighted. d) F_ST_ and e) D_XY_ calculated with pixy showing elevated values within the highlighted inversion region. f) pairwise linkage disequilibrium plot calculated using pixy where the top triangle includes all samples, and the lower triangle includes only the individuals within the common type.


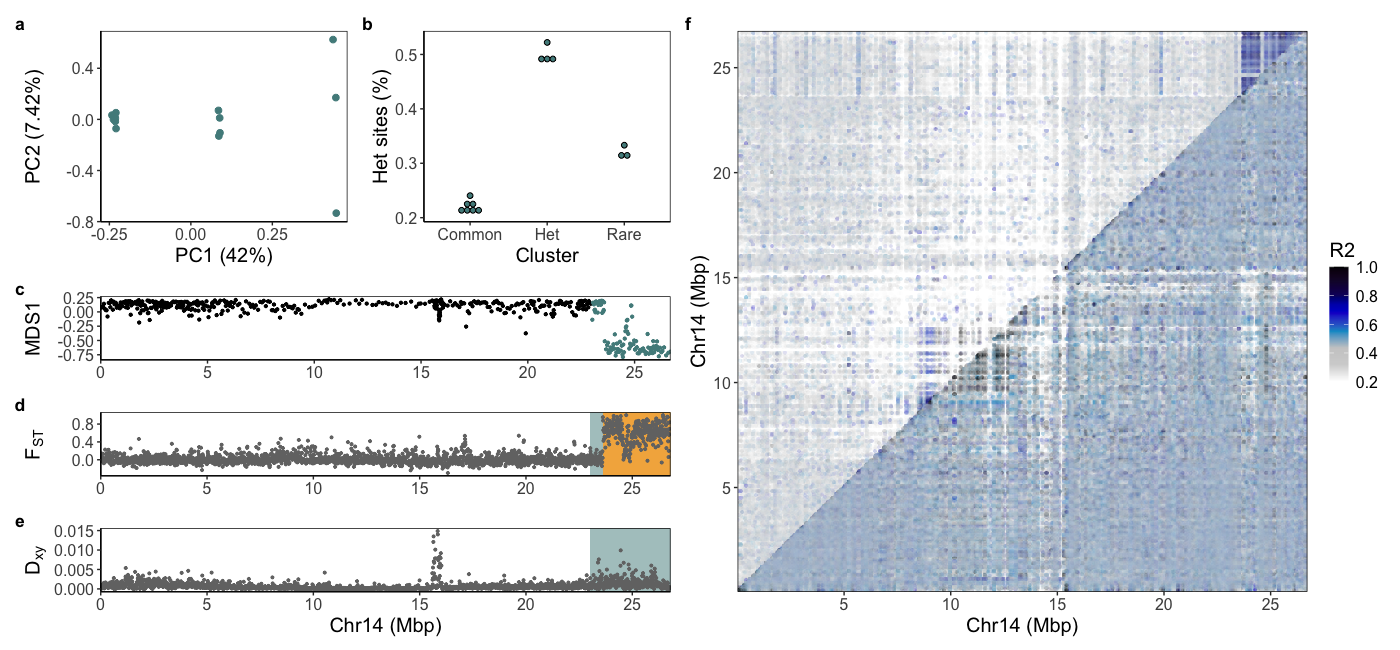


**Figure S13.** Inversion detected on chromosome 14 of Arctic cod using Arctic cod as reference genome. See Figure 5 in the main text for descriptions of the different panels. a) PCA for the inversion region identified using lostruct. b) Manually assigned cluster groups and % heterozygous sites given in bins for the clusters. c) MDS analysis produced by lostruct where the inversion region is highlighted. d) F_ST_ and e) D_XY_ calculated with pixy showing elevated values within the highlighted inversion region. f) pairwise linkage disequilibrium plot calculated using pixy where the top triangle includes all samples, and the lower triangle includes only the individuals within the common type.


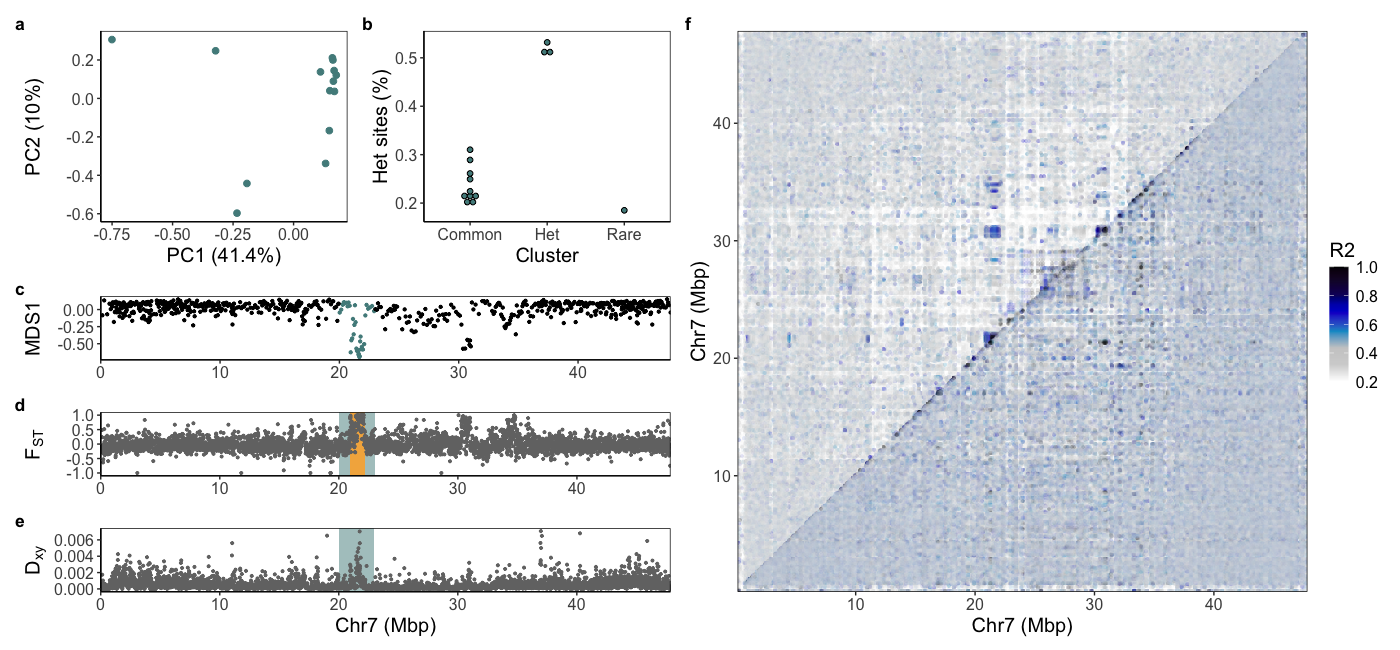
 **Figure S14.** Putative inversion detected on chromosome 7 (1) of Arctic cod using Arctic cod as reference genome. a) PCA for the inversion region identified using lostruct. b) Manually assigned cluster groups and % heterozygous sites given in bins for the clusters. c) MDS analysis produced by lostruct where the inversion region is highlighted. d) F_ST_ and e) D_XY_ calculated with pixy showing elevated values within the highlighted inversion region. f) pairwise linkage disequilibrium plot calculated using pixy where the top triangle includes all samples, and the lower triangle includes only the individuals within the common type.


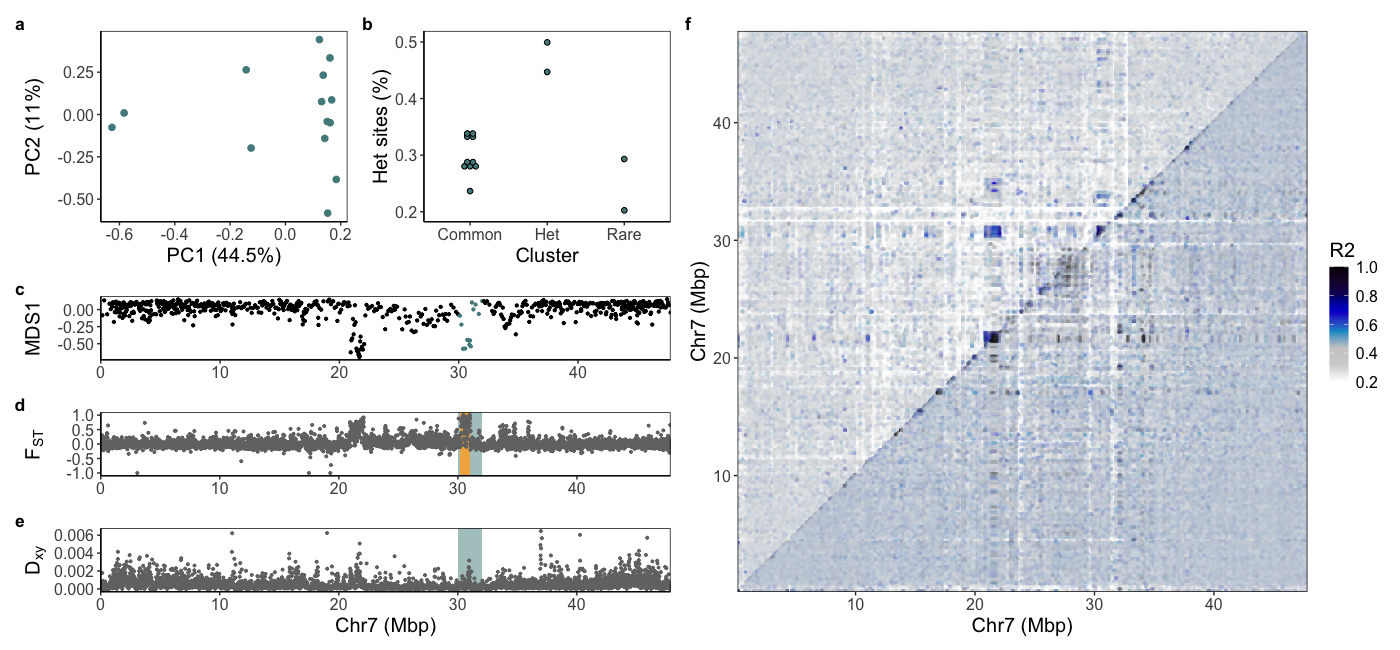


**Figure S15.** Putative inversion detected on chromosome 7 (2) of Arctic cod using Arctic cod as reference genome. a) PCA for the inversion region identified using lostruct. b) Manually assigned cluster groups and % heterozygous sites given in bins for the clusters. c) MDS analysis produced by lostruct where the inversion region is highlighted. d) F_ST_ and e) D_XY_ calculated with pixy showing elevated values within the highlighted inversion region. f) pairwise linkage disequilibrium plot calculated using pixy where the top triangle includes all samples, and the lower triangle includes only the individuals within the common type.


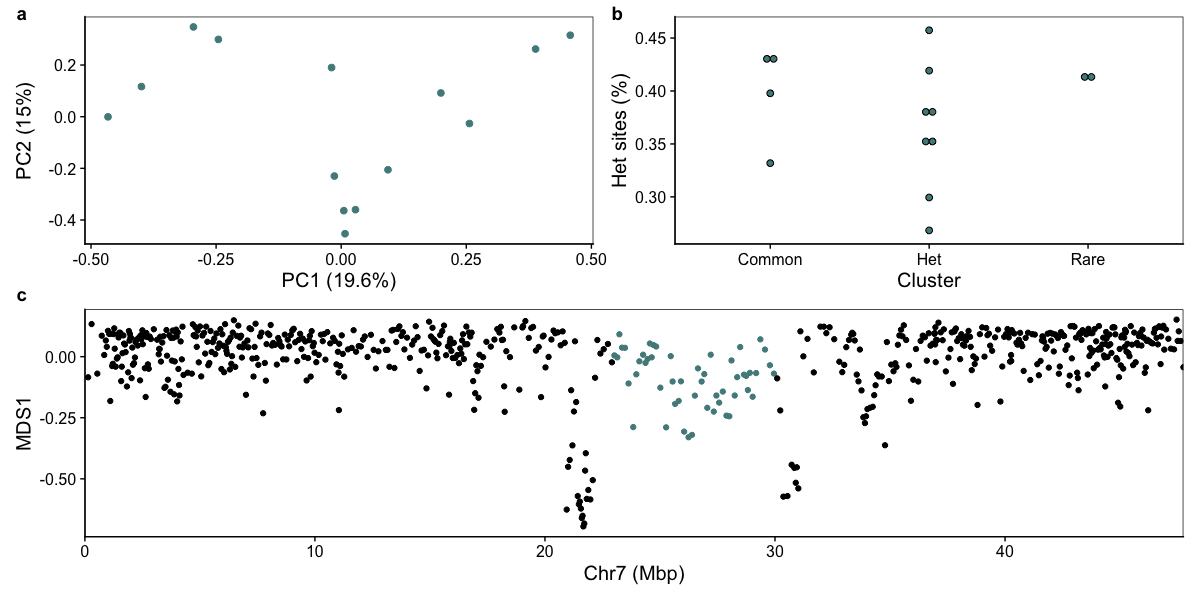


**Figure S16.** Testing for inversion signal between the two putative inversions on chromosome 7 of Arctic cod using Arctic cod as reference genome. a) PCA of the region does not show an inversion signal, b) clusters do not follow the typical inversion heterozygosity pattern, and c) MDS1 from lostruct where the region used for calculating the PCA is highlighted.


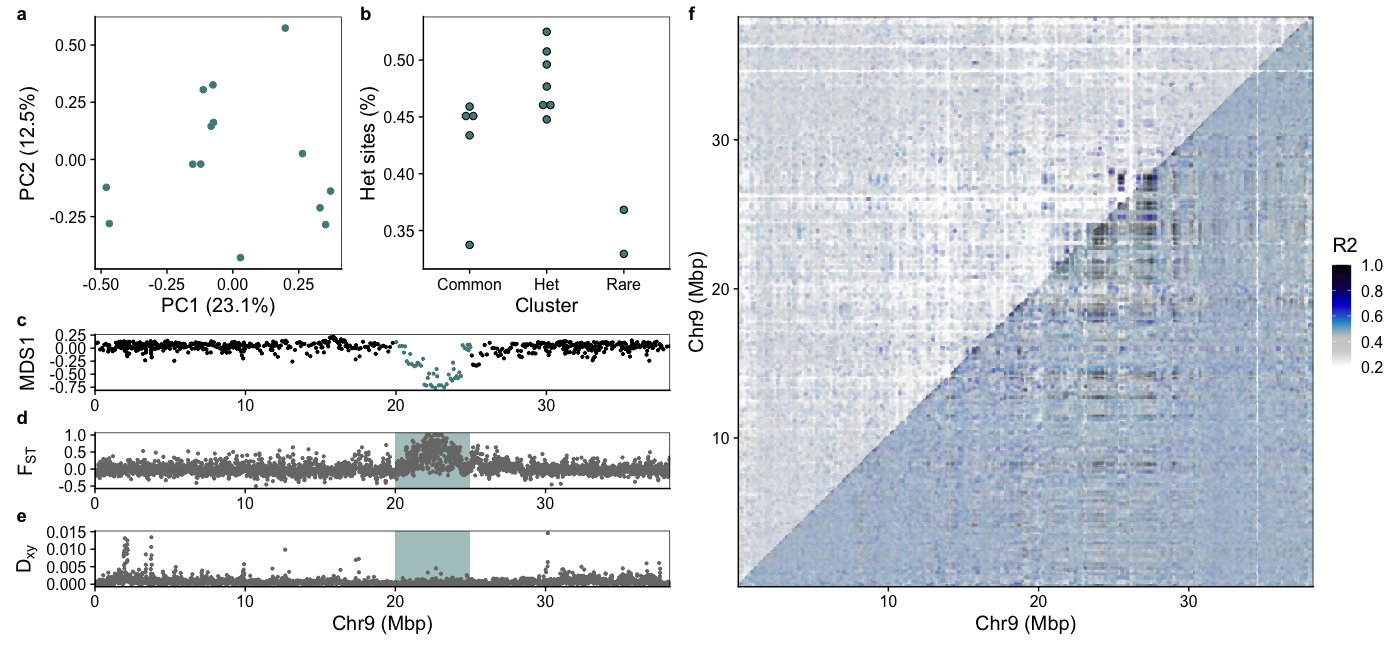


**Figure S17.** Putative inversion detected on chromosome 9 of Arctic cod using Arctic cod as reference genome. a) PCA for the inversion region identified using lostruct. b) Manually assigned cluster groups and % heterozygous sites given in bins for the clusters. c) MDS analysis produced by lostruct where the inversion region is highlighted. d) F_ST_ and e) D_XY_ calculated with pixy showing elevated values within the highlighted inversion region. f) pairwise linkage disequilibrium plot calculated using pixy where the top triangle includes all samples, and the lower triangle includes only the individuals within the common type.


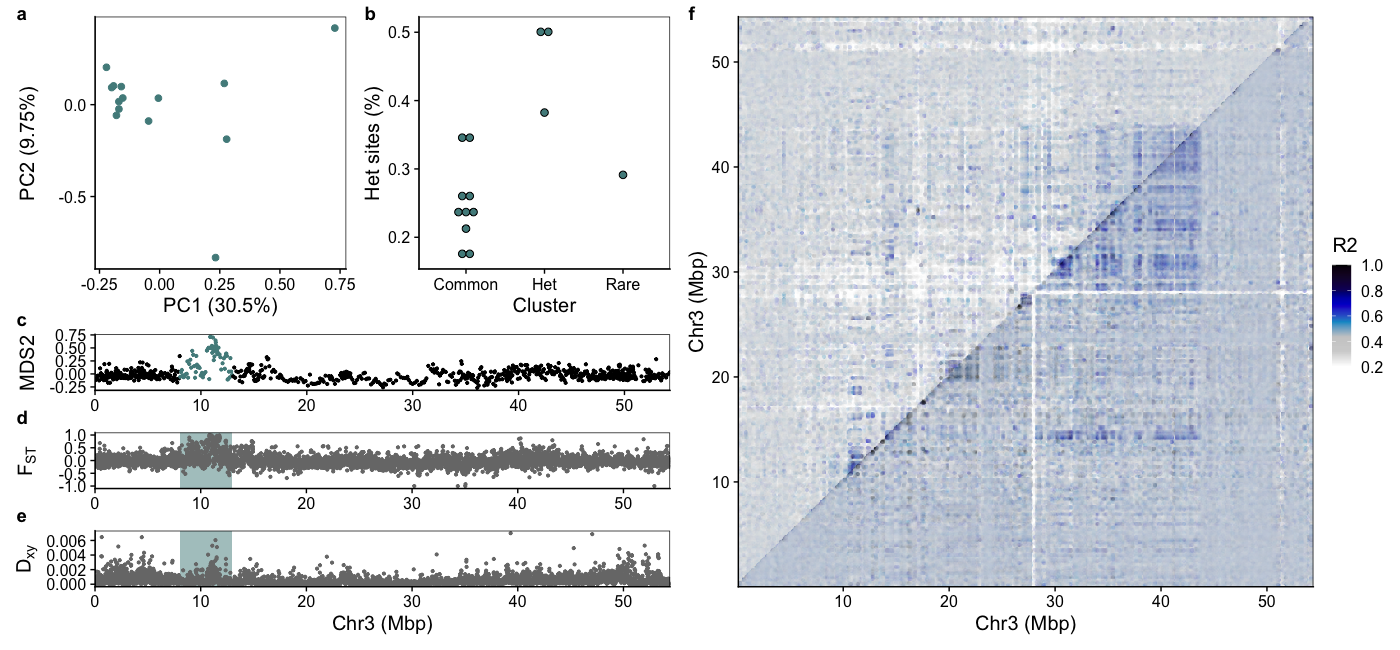


**Figure S18:** Putative inversion detected on chromosome 3 of Arctic cod using Arctic cod as reference genome. a) PCA for the inversion region identified using lostruct. b) Manually assigned cluster groups and % sites given in bins for the clusters. c) MDS analysis produced by lostruct where the inversion region is highlighted. d) F_ST_ and e) D_XY_ calculated with pixy showing elevated values within the highlighted inversion region. f) pairwise linkage disequilibrium plot calculated using pixy where the top triangle includes all samples, and the lower triangle includes only the individuals within the common type.


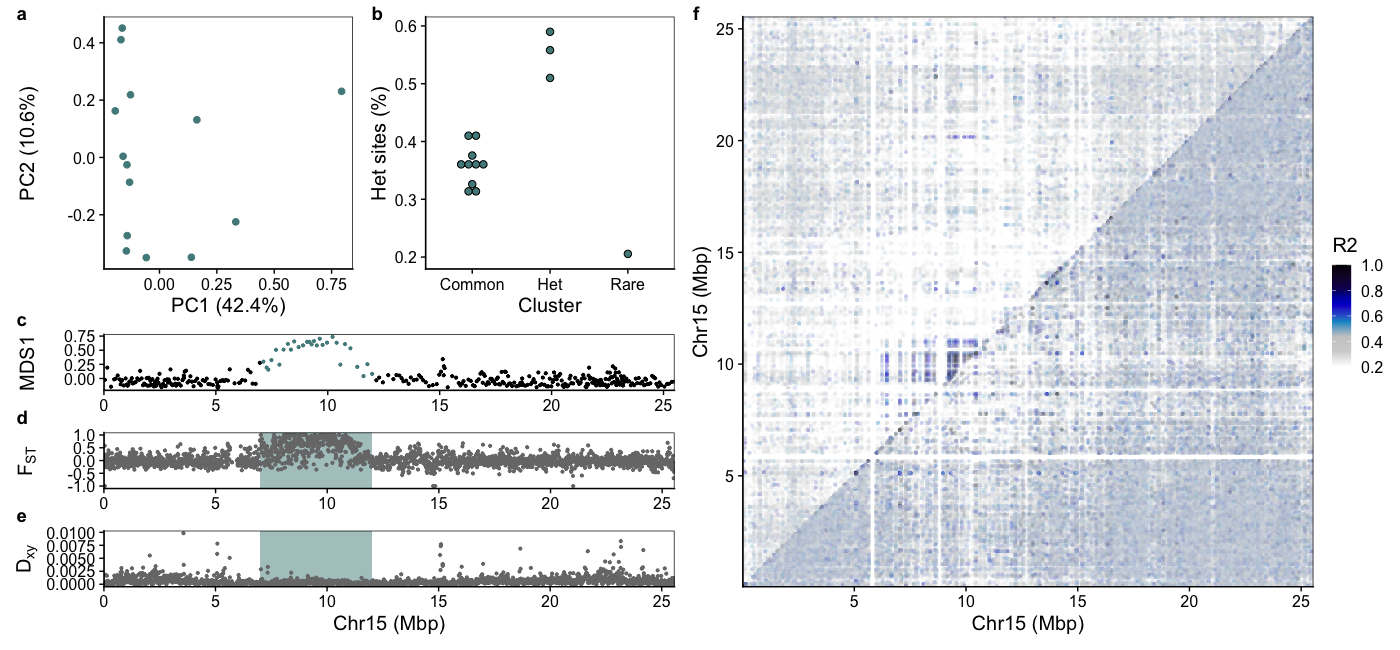


**Figure S19:** Putative inversion detected on chromosome 15 of Arctic cod using Arctic cod as reference genome. a) PCA for the inversion region identified using lostruct. b) Manually assigned cluster groups and % heterozygous sites given in bins for the clusters. c) MDS analysis produced by lostruct where the inversion region is highlighted. d) F_ST_ and e) D_XY_ calculated with pixy showing elevated values within the highlighted inversion region. f) pairwise linkage disequilibrium plot calculated using pixy where the top triangle includes all samples, and the lower triangle includes only the individuals within the common type.


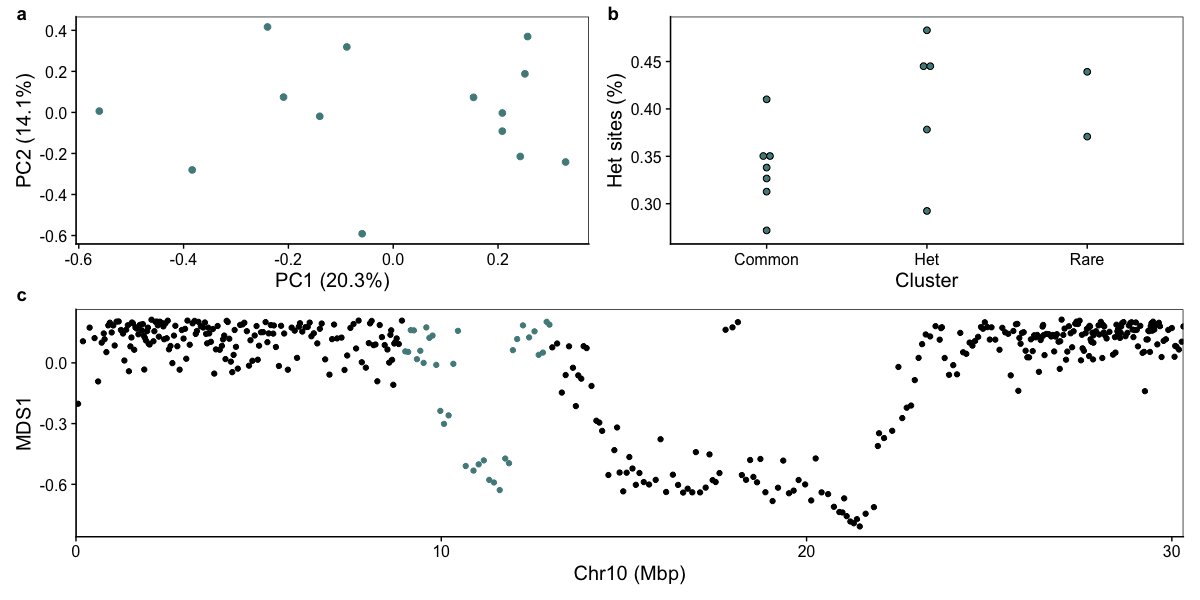


**Figure S20.** Testing for inversion signal for a region of differentiation before the inversion of chromosome 10 of Arctic cod using Arctic cod as reference genome. a) PCA of the region does not show a clear inversion signal, b) clusters have a weak heterozygosity pattern, and c) MDS1 from lostruct where the region used for calculating the PCA is highlighted.

### **Inversion detection plots using NEAC as reference genome**

**
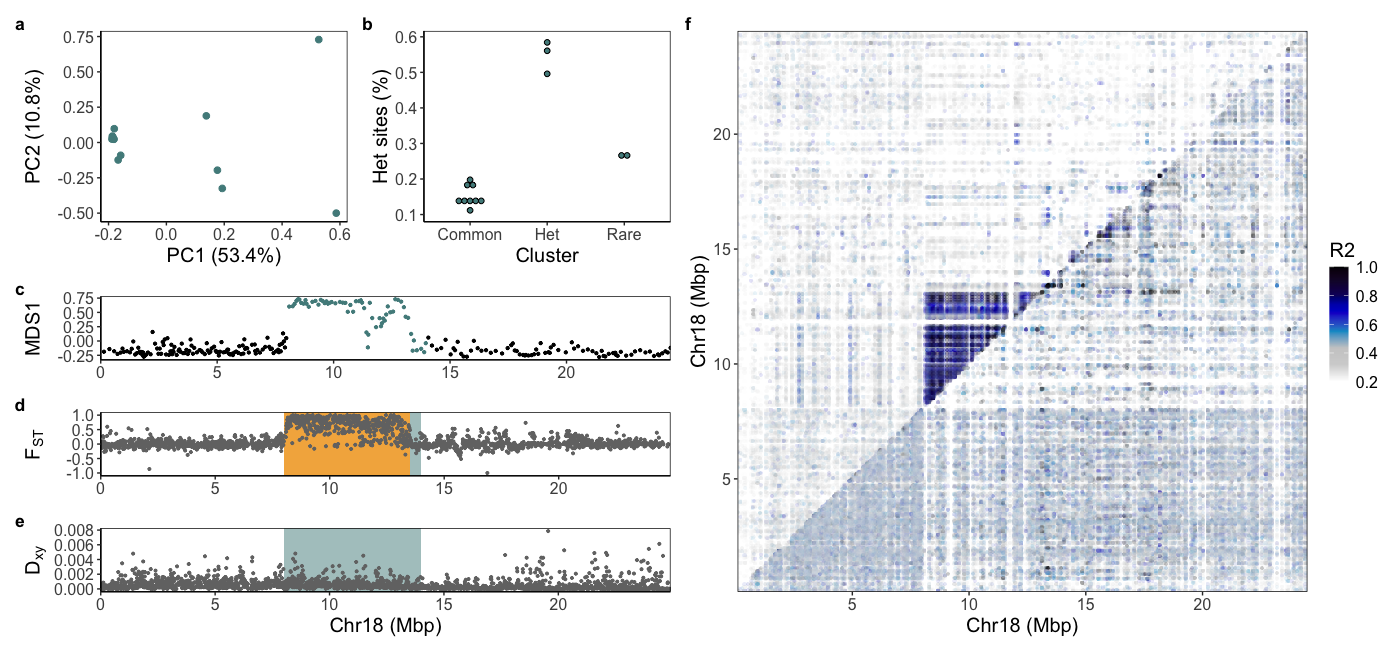
**

**Figure S21.** Inversion detected on chromosome 18 (Arctic cod Chr1) using NEAC as reference genome. a) PCA for the inversion region identified using lostruct. b) Manually assigned cluster groups and % heterozygous sites given in bins for the clusters. c) MDS analysis produced by lostruct where the inversion region is highlighted. d) F_ST_ and e) D_XY_ calculated with pixy showing elevated values within the highlighted inversion region. f) pairwise linkage disequilibrium plot calculated using pixy where the top triangle includes all samples, and the lower triangle includes only the individuals within the common type.

**
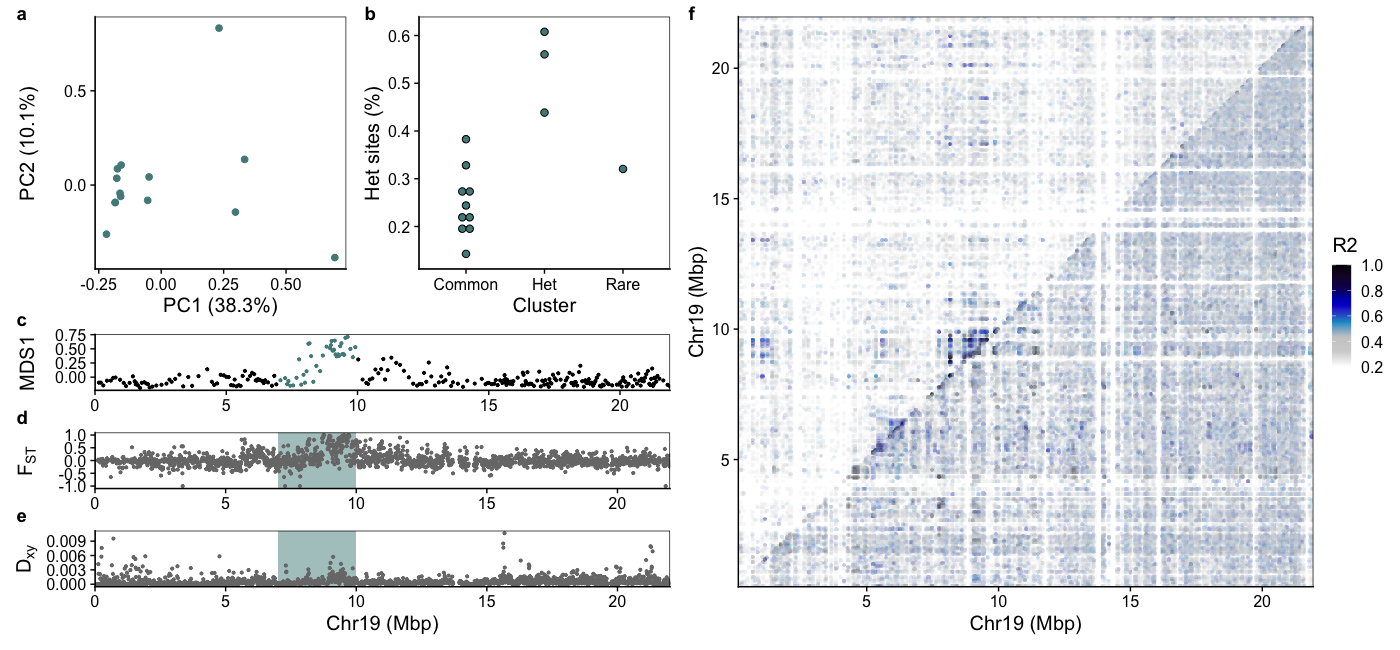
**

**Figure S22.** Putative inversion detected on chromosome 19 (Arctic cod Chr3) using NEAC as reference genome. a) PCA for the inversion region identified using lostruct. b) Manually assigned cluster groups and % heterozygous sites given in bins for the clusters. c) MDS analysis produced by lostruct where the inversion region is highlighted. d) F_ST_ and e) D_XY_ calculated with pixy showing elevated values within the highlighted inversion region. f) pairwise linkage disequilibrium plot calculated using pixy where the top triangle includes all samples, and the lower triangle includes only the individuals within the common type.


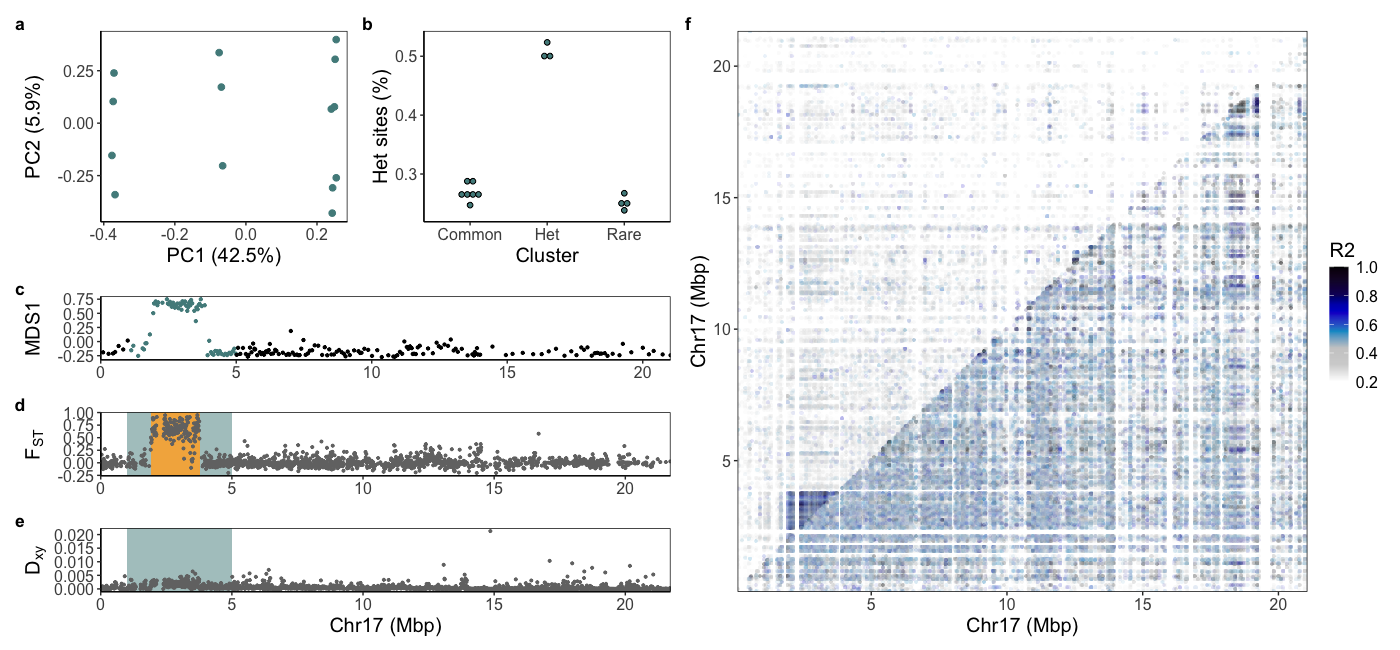


**Figure S23.** Inversion detected on chromosome 17 (Arctic cod Chr6) using NEAC as reference genome. a) PCA for the inversion region identified using lostruct. b) Manually assigned cluster groups and % heterozygous sites given in bins for the clusters. c) MDS analysis produced by lostruct where the inversion region is highlighted. d) F_ST_ and e) D_XY_ calculated with pixy showing elevated values within the highlighted inversion region. f) pairwise linkage disequilibrium plot calculated using pixy where the top triangle includes all samples, and the lower triangle includes only the individuals within the common type.


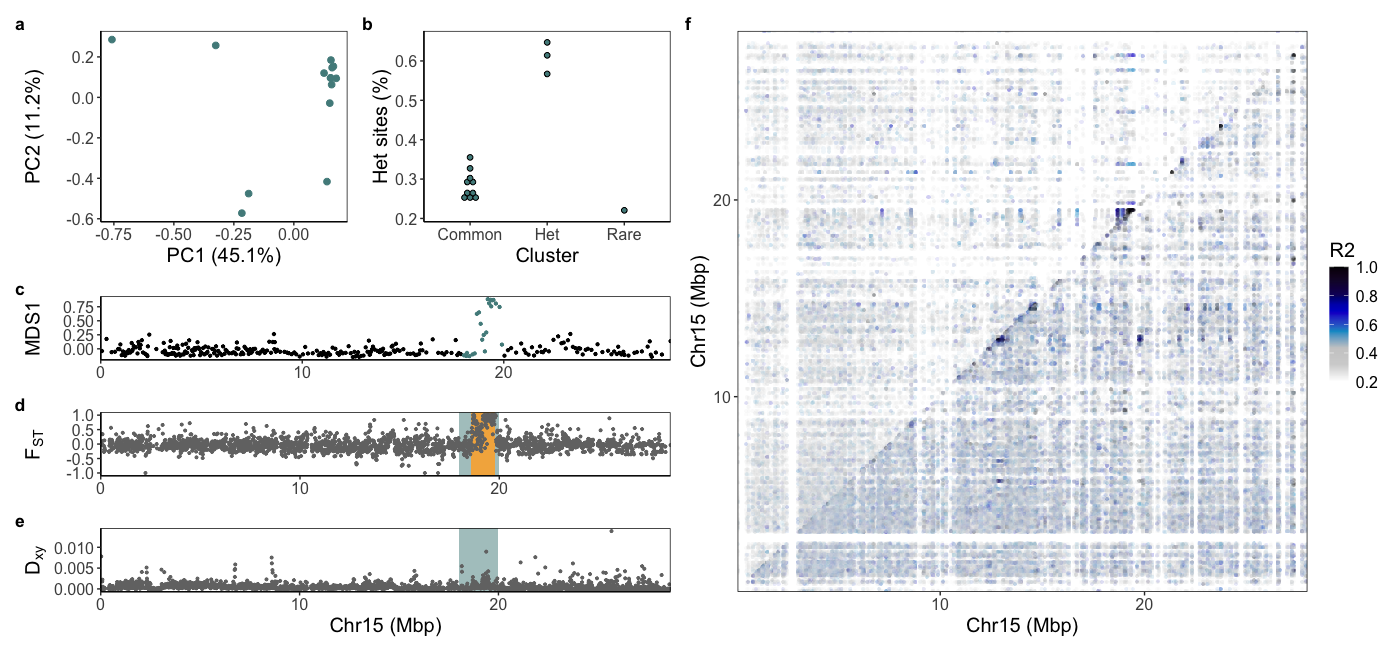


**Figure S24.** Putative inversion detected on chromosome 15 (Arctic cod Chr7 (1)) using NEAC as reference genome. a) PCA for the inversion region identified using lostruct. b) Manually assigned cluster groups and % heterozygous sites given in bins for the clusters. c) MDS analysis produced by lostruct where the inversion region is highlighted. d) F_ST_ and e) D_XY_ calculated with pixy showing elevated values within the highlighted inversion region. f) pairwise linkage disequilibrium plot calculated using pixy where the top triangle includes all samples, and the lower triangle includes only the individuals within the common type.


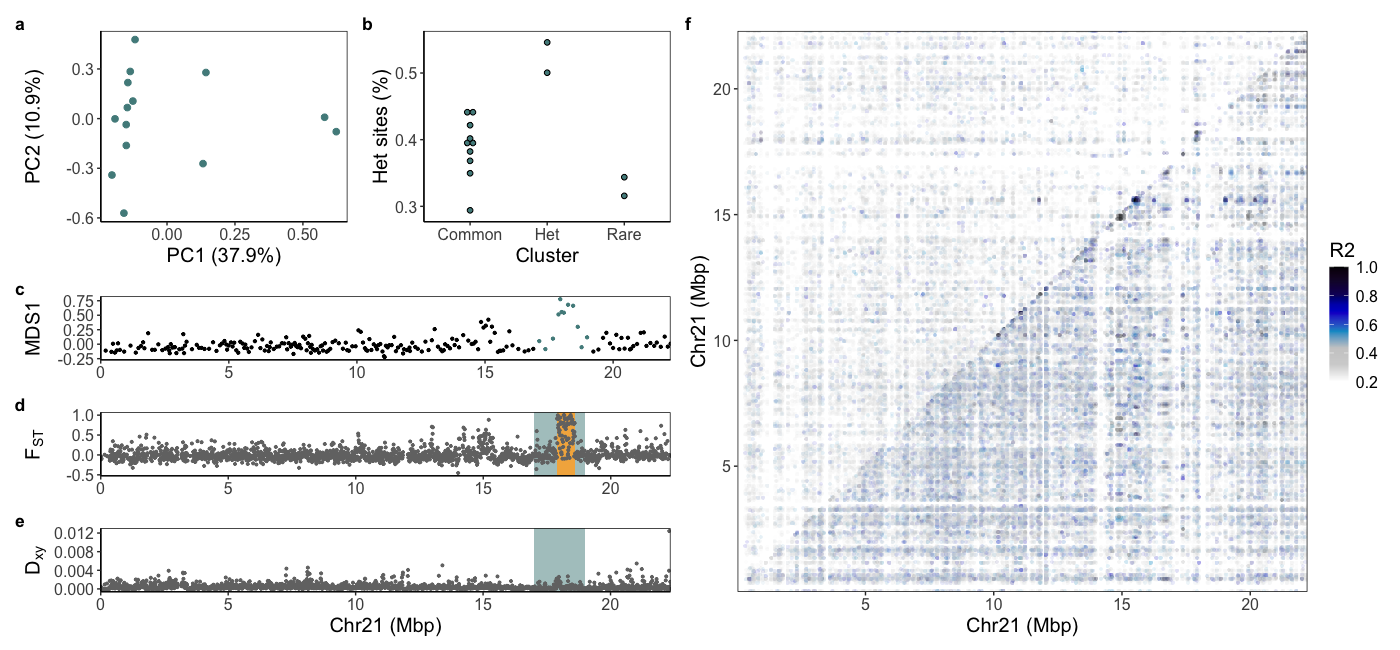


**Figure S25.** Putative inversion detected on chromosome 21 (Arctic cod Chr7 (2)) using NEAC as reference genome. a) PCA for the inversion region identified using lostruct. b) Manually assigned cluster groups and % heterozygous sites given in bins for the clusters. c) MDS analysis produced by lostruct where the inversion region is highlighted. d) F_ST_ and e) D_XY_ calculated with pixy showing elevated values within the highlighted inversion region. f) pairwise linkage disequilibrium plot calculated using pixy where the top triangle includes all samples, and the lower triangle includes only the individuals within the common type.


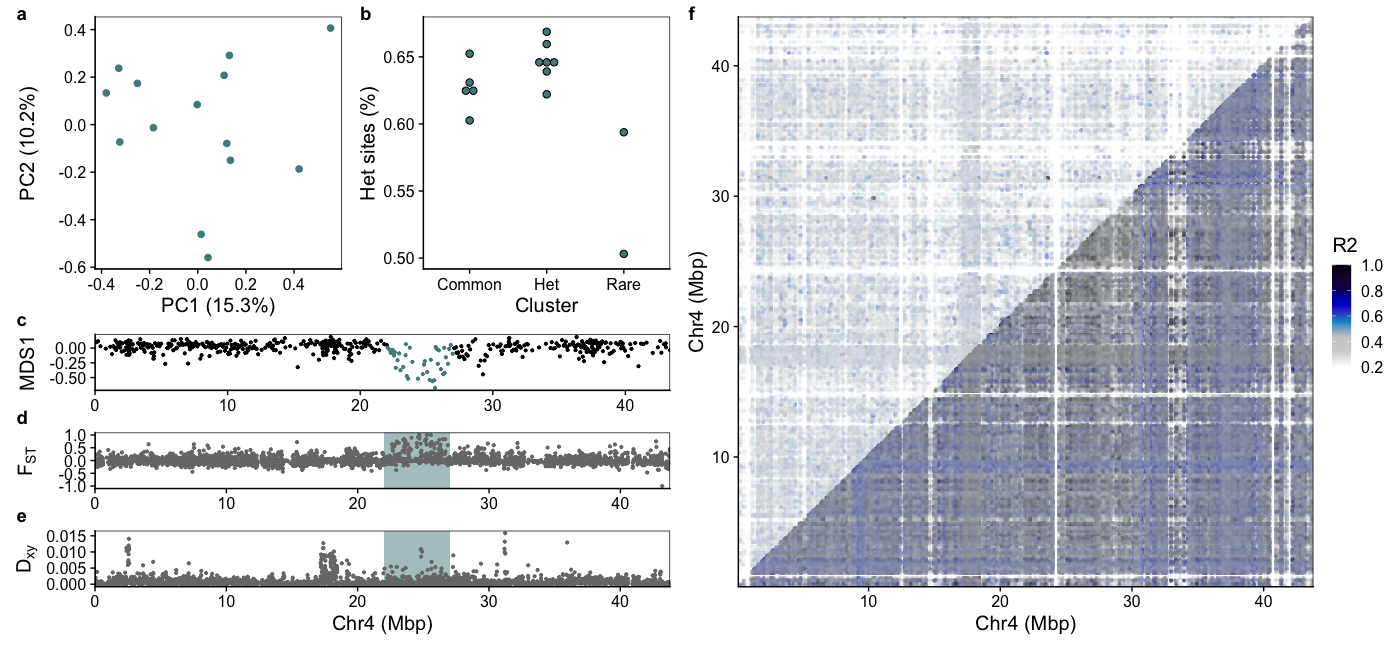


**Figure S26.** Putative inversion detected on chromosome 4 (Arctic cod Chr9) using NEAC as reference genome. a) PCA for the inversion region identified using lostruct. b) Manually assigned cluster groups and % heterozygous sites given in bins for the clusters. c) MDS analysis produced by lostruct where the inversion region is highlighted. d) F_ST_ and e) D_XY_ calculated with pixy showing elevated values within the highlighted inversion region. f) pairwise linkage disequilibrium plot calculated using pixy where the top triangle includes all samples, and the lower triangle includes only the individuals within the common type.


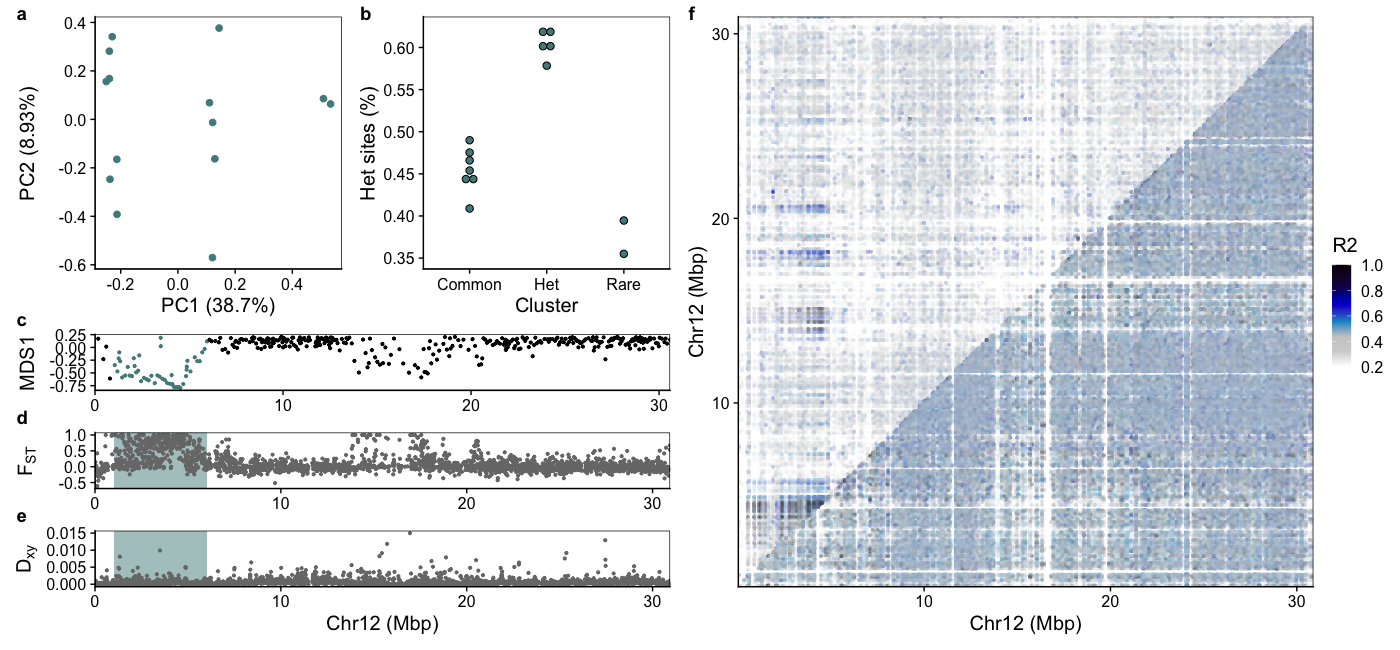


**Figure S27.** Inversion detected on chromosome 12 (Arctic cod Chr10) using NEAC as reference genome. a) PCA for the inversion region identified using lostruct. b) Manually assigned cluster groups and % heterozygous sites given in bins for the clusters. c) MDS analysis produced by lostruct where the inversion region is highlighted. d) F_ST_ and e) D_XY_ calculated with pixy showing elevated values within the highlighted inversion region. f) pairwise linkage disequilibrium plot calculated using pixy where the top triangle includes all samples, and the lower triangle includes only the individuals within the common type.


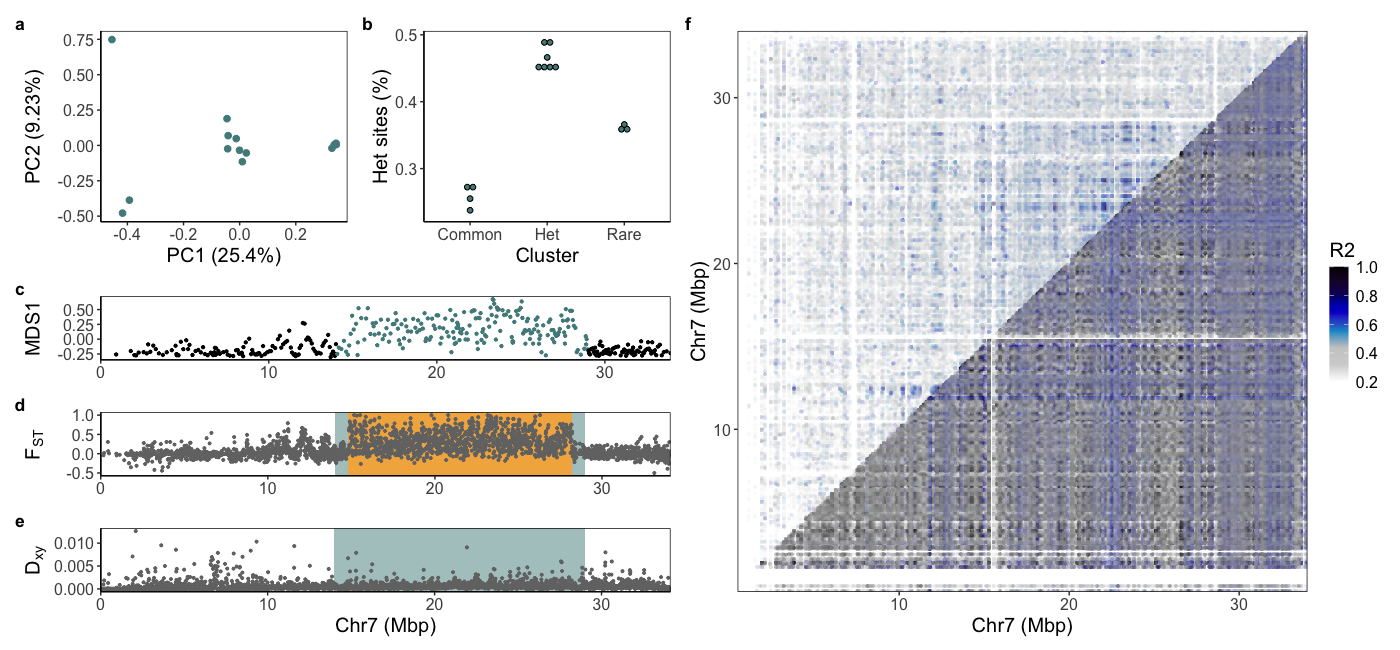


**Figure S28.** Inversion detected on chromosome 7 (Arctic cod Chr11) using NEAC as reference genome. a) PCA for the inversion region identified using lostruct. b) Manually assigned cluster groups and % heterozygous sites given in bins for the clusters. c) MDS analysis produced by lostruct where the inversion region is highlighted. d) F_ST_ and e) D_XY_ calculated with pixy showing elevated values within the highlighted inversion region. f) pairwise linkage disequilibrium plot calculated using pixy where the top triangle includes all samples, and the lower triangle includes only the individuals within the common type.


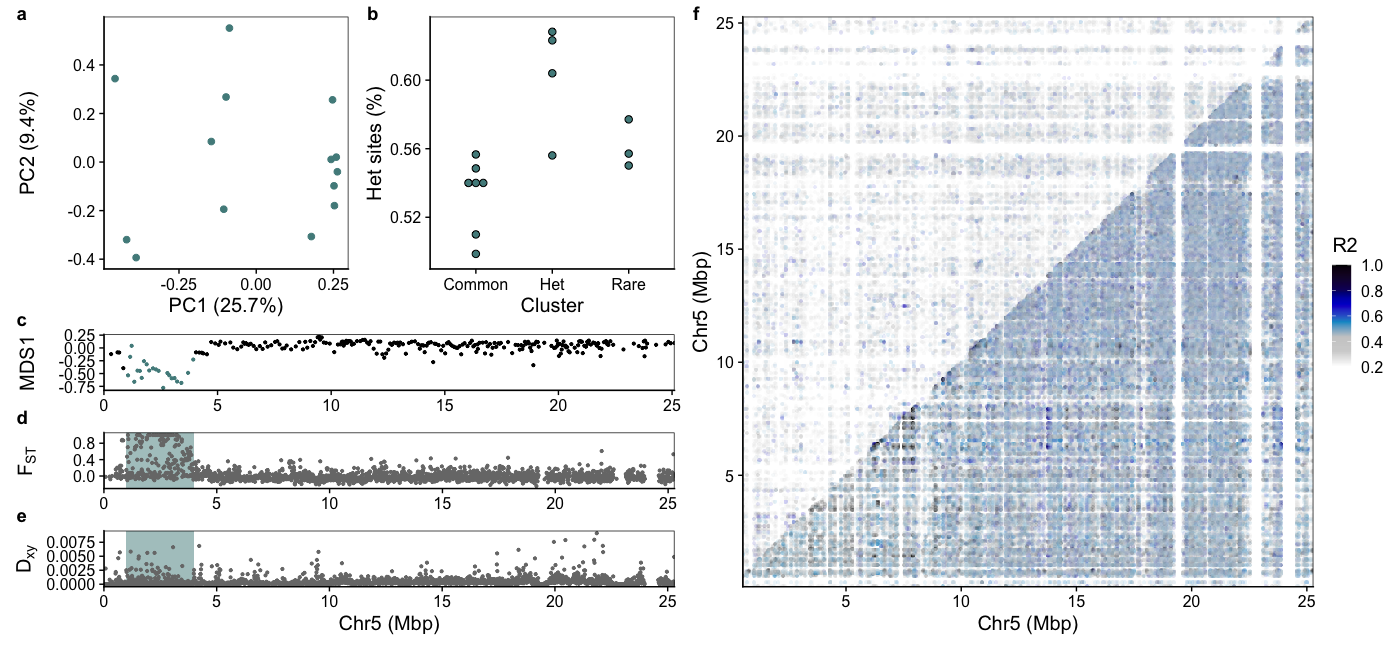


**Figure S29.** Inversion detected on chromosome 5 (Arctic cod Chr13) using NEAC as reference genome. a) PCA for the inversion region identified using lostruct. b) Manually assigned cluster groups and % heterozygous sites given in bins for the clusters. c) MDS analysis produced by lostruct where the inversion region is highlighted. d) F_ST_ and e) D_XY_ calculated with pixy showing elevated values within the highlighted inversion region. f) pairwise linkage disequilibrium plot calculated using pixy where the top triangle includes all samples, and the lower triangle includes only the individuals within the common type.


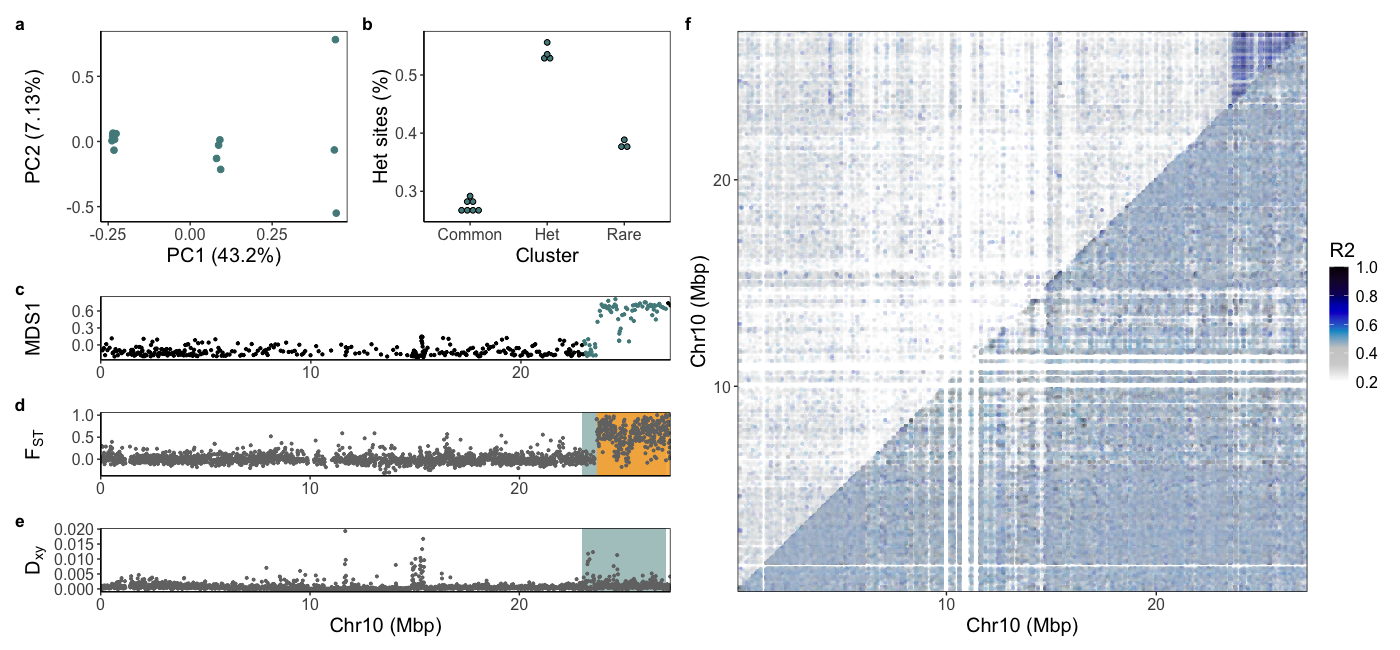


**Figure S30.** Inversion detected on chromosome 10 (Arctic cod Chr14) using NEAC as reference genome. a) PCA for the inversion region identified using lostruct. b) Manually assigned cluster groups and % heterozygous sites given in bins for the clusters. c) MDS analysis produced by lostruct where the inversion region is highlighted. d) F_ST_ and e) D_XY_ calculated with pixy showing elevated values within the highlighted inversion region. f) pairwise linkage disequilibrium plot calculated using pixy where the top triangle includes all samples, and the lower triangle includes only the individuals within the common type.


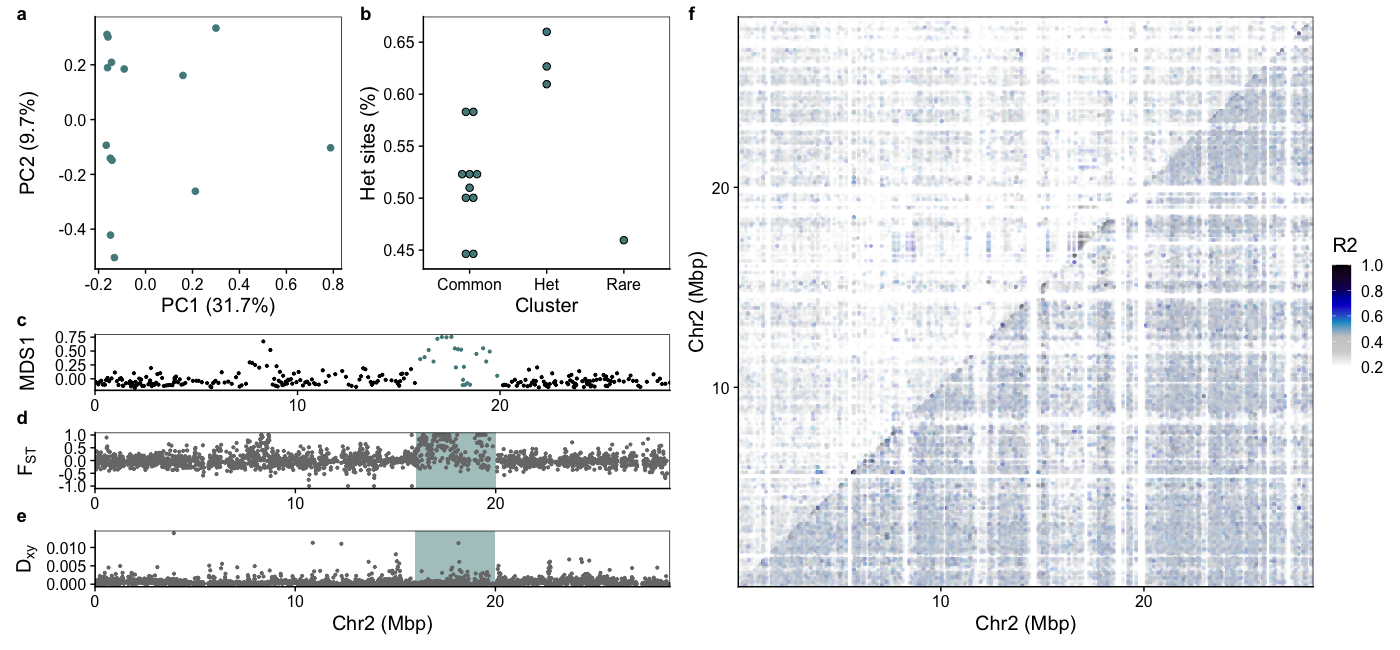


**Figure S31.** Putative inversion detected on chromosome 2 (Arctic cod Chr15) using NEAC as reference genome. a) PCA for the inversion region identified using lostruct. b) Manually assigned cluster groups and % heterozygous sites given in bins for the clusters. c) MDS analysis produced by lostruct where the inversion region is highlighted. d) F_ST_ and e) D_XY_ calculated with pixy showing elevated values within the highlighted inversion region. f) pairwise linkage disequilibrium plot calculated using pixy where the top triangle includes all samples, and the lower triangle includes only the individuals within the common type.

### **Inversion detection plots using polar cod as reference genome**


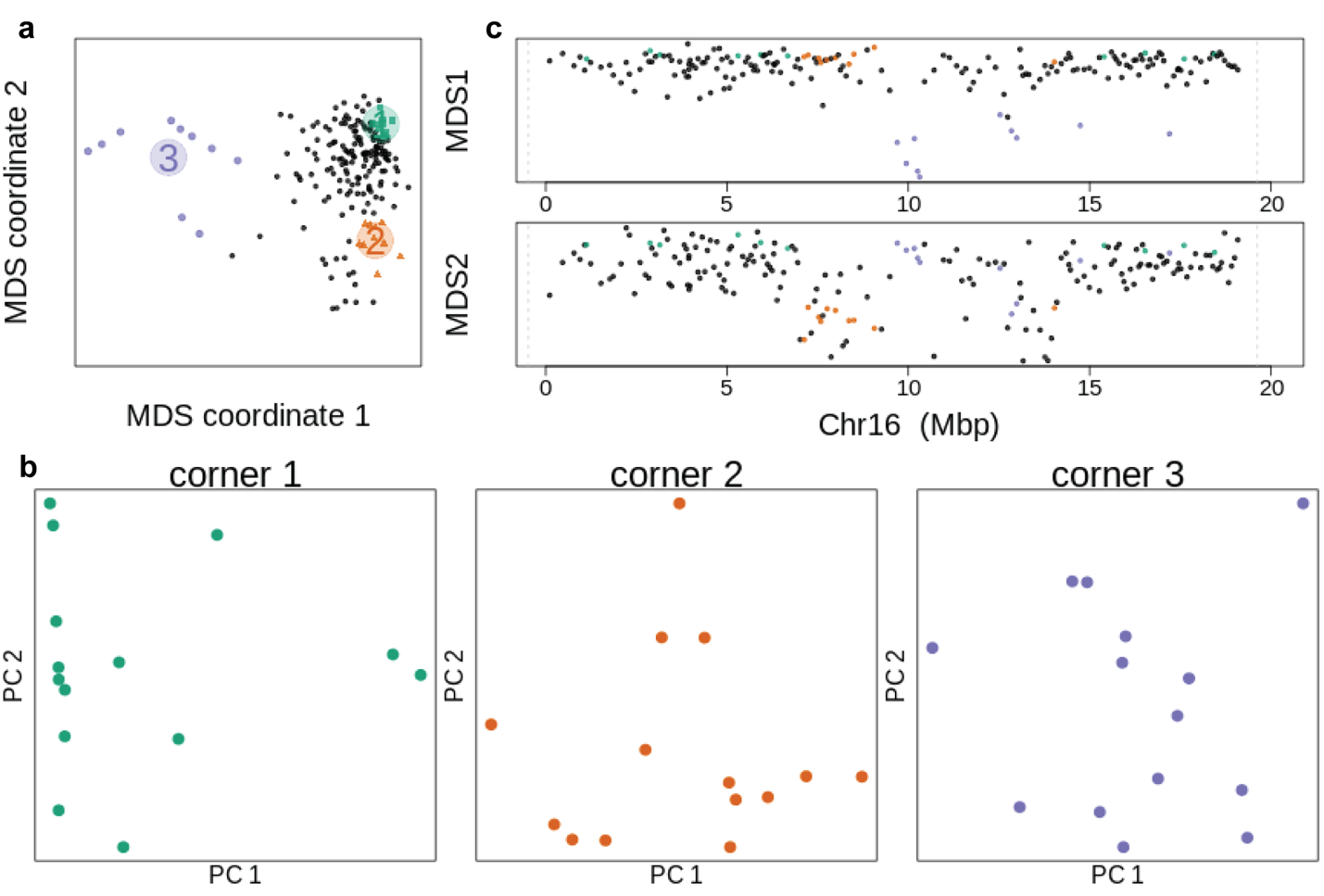


**Figure S32.** Lostruct plot showing no putative inversion signal on chromosome 16 (Arctic cod Chr7 (2)) using polar cod as reference genome. a) MDS plots, b) PCA for each MDS corner, and c) PCA windows along the chromosome.

**
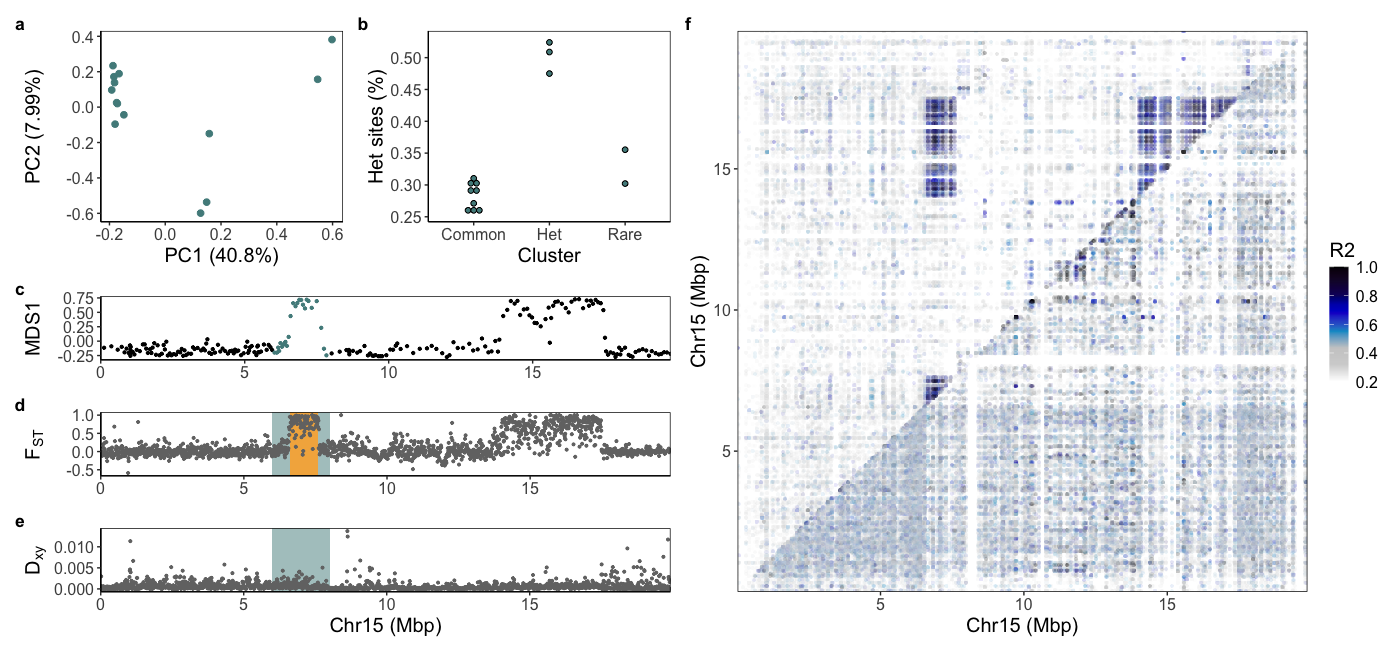
**

**Figure S33.** Split inversion detected on chromosome 15 (Arctic cod Chr1) using polar cod as reference genome. a) PCA for the inversion region identified using lostruct. b) Manually assigned cluster groups and % heterozygous sites given in bins for the clusters. c) MDS analysis produced by lostruct where the inversion region is highlighted. d) F_ST_ and e) D_XY_ calculated with pixy showing elevated values within the highlighted inversion region. f) pairwise linkage disequilibrium plot calculated using pixy where the top triangle includes all samples, and the lower triangle includes only the individuals within the common type.


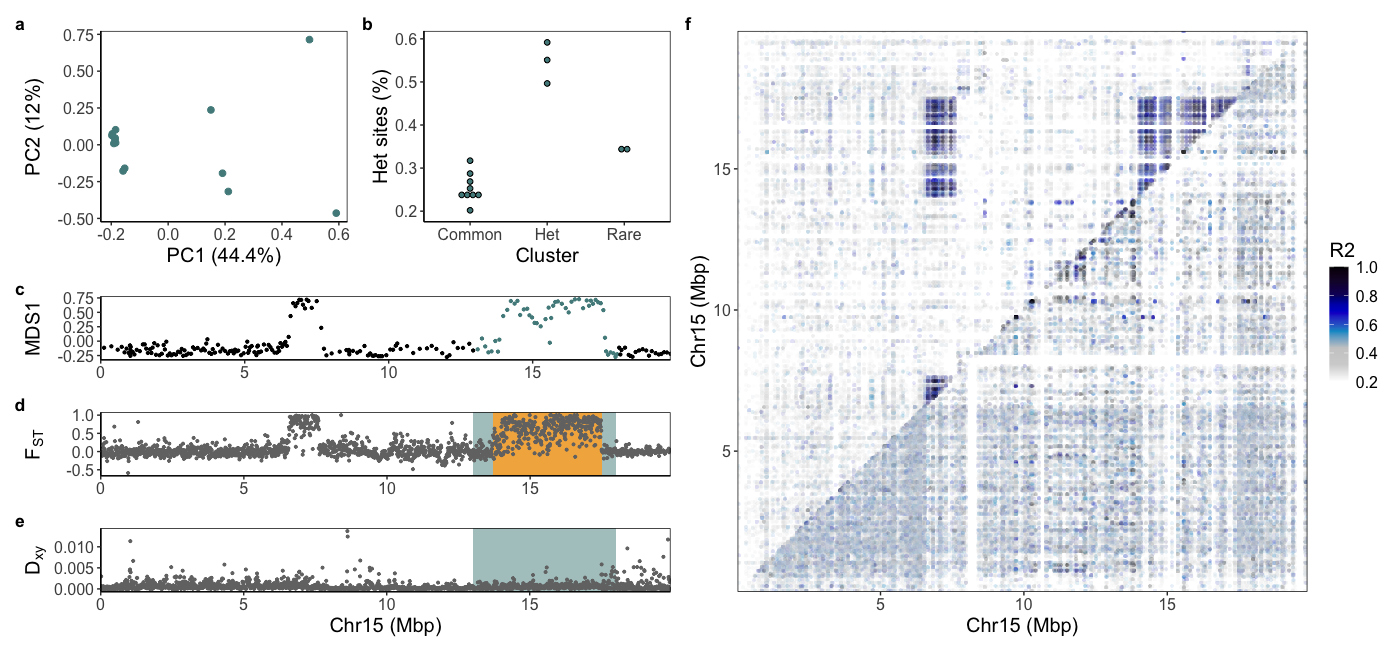


**Figure S34.** Split inversion detected on chromosome 15 (Arctic cod Chr1) using polar cod as reference genome. a) PCA for the inversion region identified using lostruct. b) Manually assigned cluster groups and % heterozygous sites given in bins for the clusters. c) MDS analysis produced by lostruct where the inversion region is highlighted. d) F_ST_ and e) D_XY_ calculated with pixy showing elevated values within the highlighted inversion region. f) pairwise linkage disequilibrium plot calculated using pixy where the top triangle includes all samples, and the lower triangle includes only the individuals within the common type.


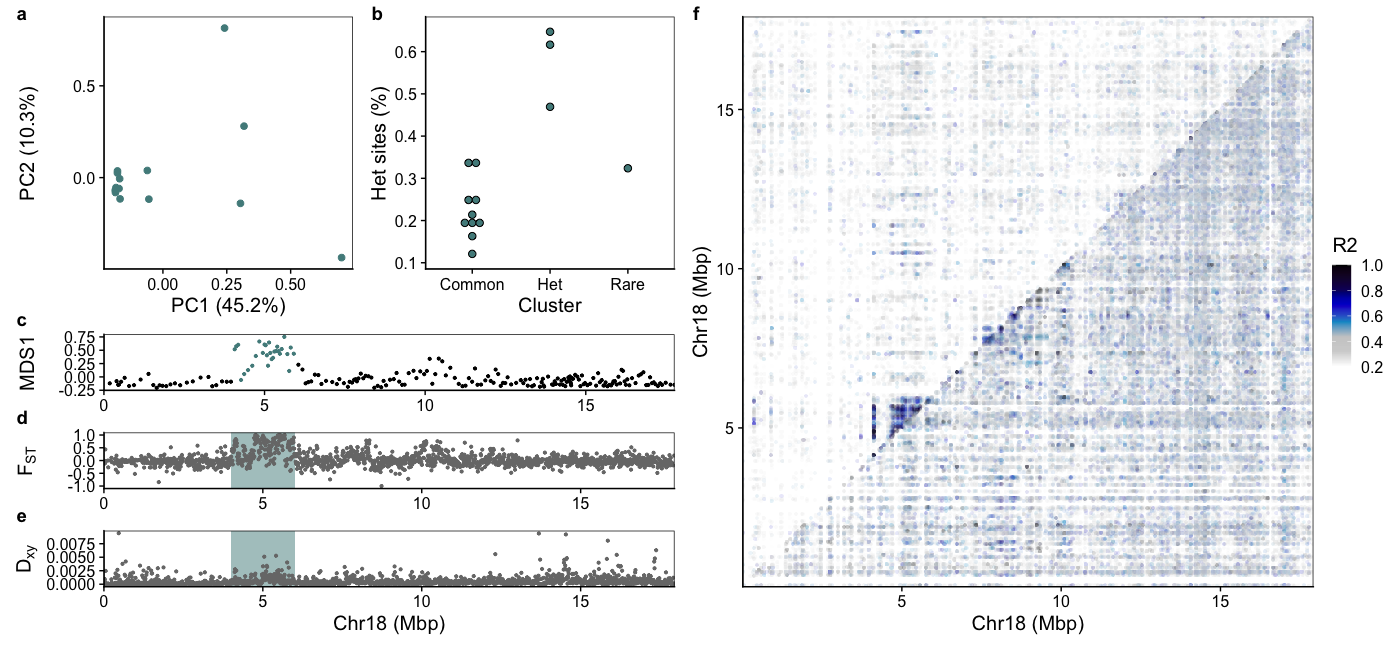


**Figure S35.** Putative inversion detected on chromosome 18 (Arctic cod Chr3) using polar cod as reference genome. a) PCA for the inversion region identified using lostruct. b) Manually assigned cluster groups and % heterozygous sites given in bins for the clusters. c) MDS analysis produced by lostruct where the inversion region is highlighted. d) F_ST_ and e) D_XY_ calculated with pixy showing elevated values within the highlighted inversion region. f) pairwise linkage disequilibrium plot calculated using pixy where the top triangle includes all samples, and the lower triangle includes only the individuals within the common type.

**
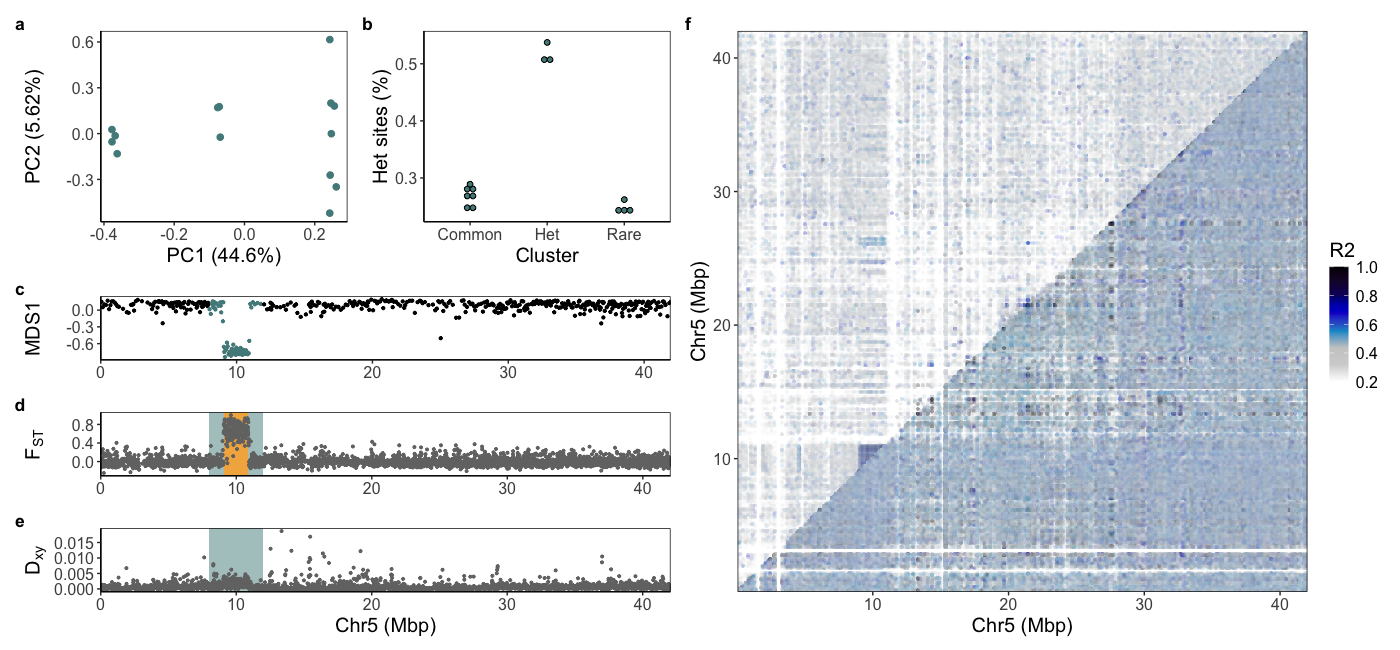
**

**Figure S36.** Inversion detected on chromosome 5 (Arctic cod Chr6) using polar cod as reference genome. a) PCA for the inversion region identified using lostruct. b) Manually assigned cluster groups and % heterozygous sites given in bins for the clusters. c) MDS analysis produced by lostruct where the inversion region is highlighted. d) F_ST_ and e) D_XY_ calculated with pixy showing elevated values within the highlighted inversion region. f) pairwise linkage disequilibrium plot calculated using pixy where the top triangle includes all samples, and the lower triangle includes only the individuals within the common type.

**
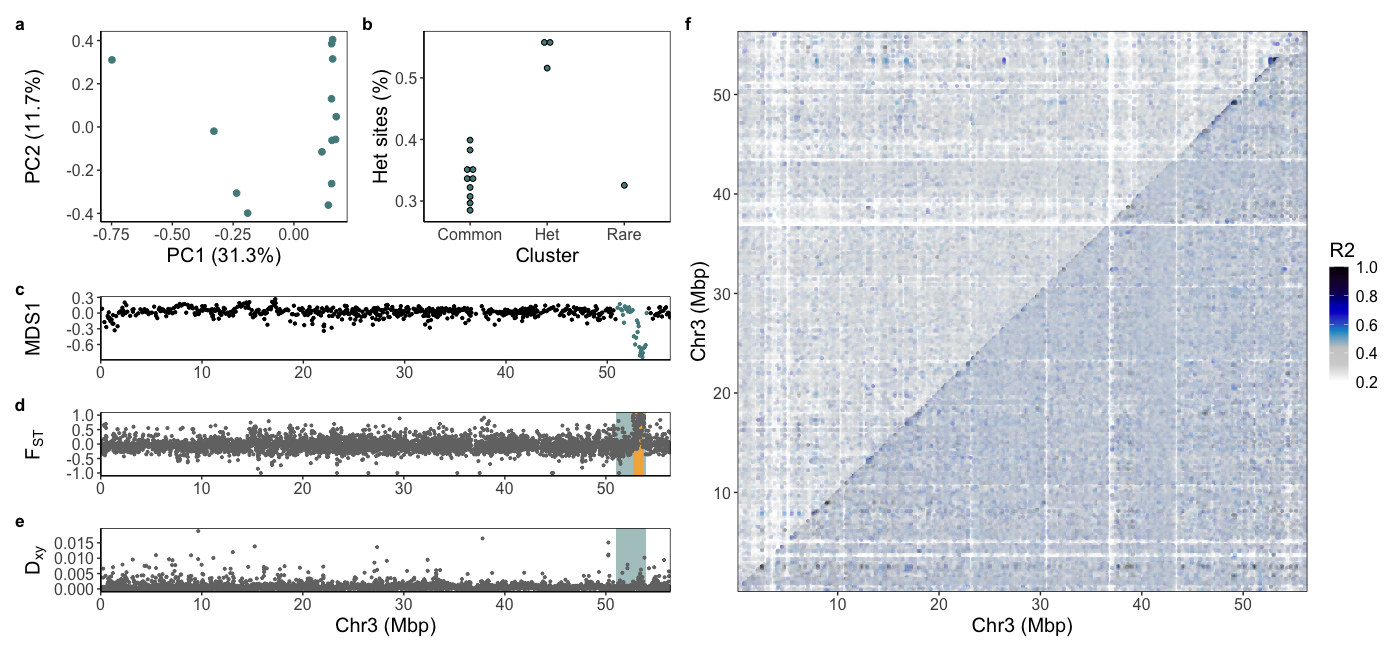
**

**Figure S37.** Putative inversion detected on chromosome 3 (Arctic cod Chr7 (1)) using polar cod as reference genome. a) PCA for the inversion region identified using lostruct. b) Manually assigned cluster groups and % heterozygous sites given in bins for the clusters. c) MDS analysis produced by lostruct where the inversion region is highlighted. d) F_ST_ and e) D_XY_ calculated with pixy showing elevated values within the highlighted inversion region. f) pairwise linkage disequilibrium plot calculated using pixy where the top triangle includes all samples, and the lower triangle includes only the individuals within the common type.

**
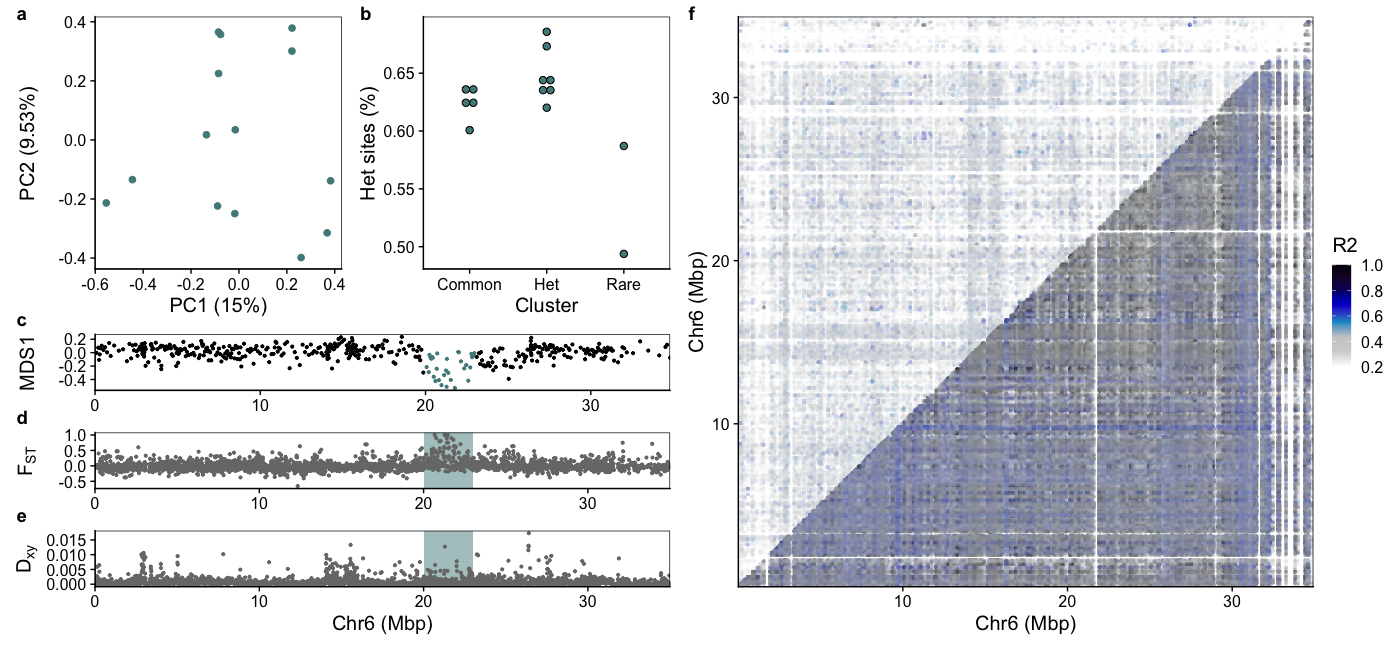
**

**Figure S38.** Putative inversion detected on chromosome 6 (Arctic cod Chr9) using polar cod as reference genome. a) PCA for the inversion region identified using lostruct. b) Manually assigned cluster groups and % heterozygous sites given in bins for the clusters. c) MDS analysis produced by lostruct where the inversion region is highlighted. d) F_ST_ and e) D_XY_ calculated with pixy showing elevated values within the highlighted inversion region. f) pairwise linkage disequilibrium plot calculated using pixy where the top triangle includes all samples, and the lower triangle includes only the individuals within the common type.


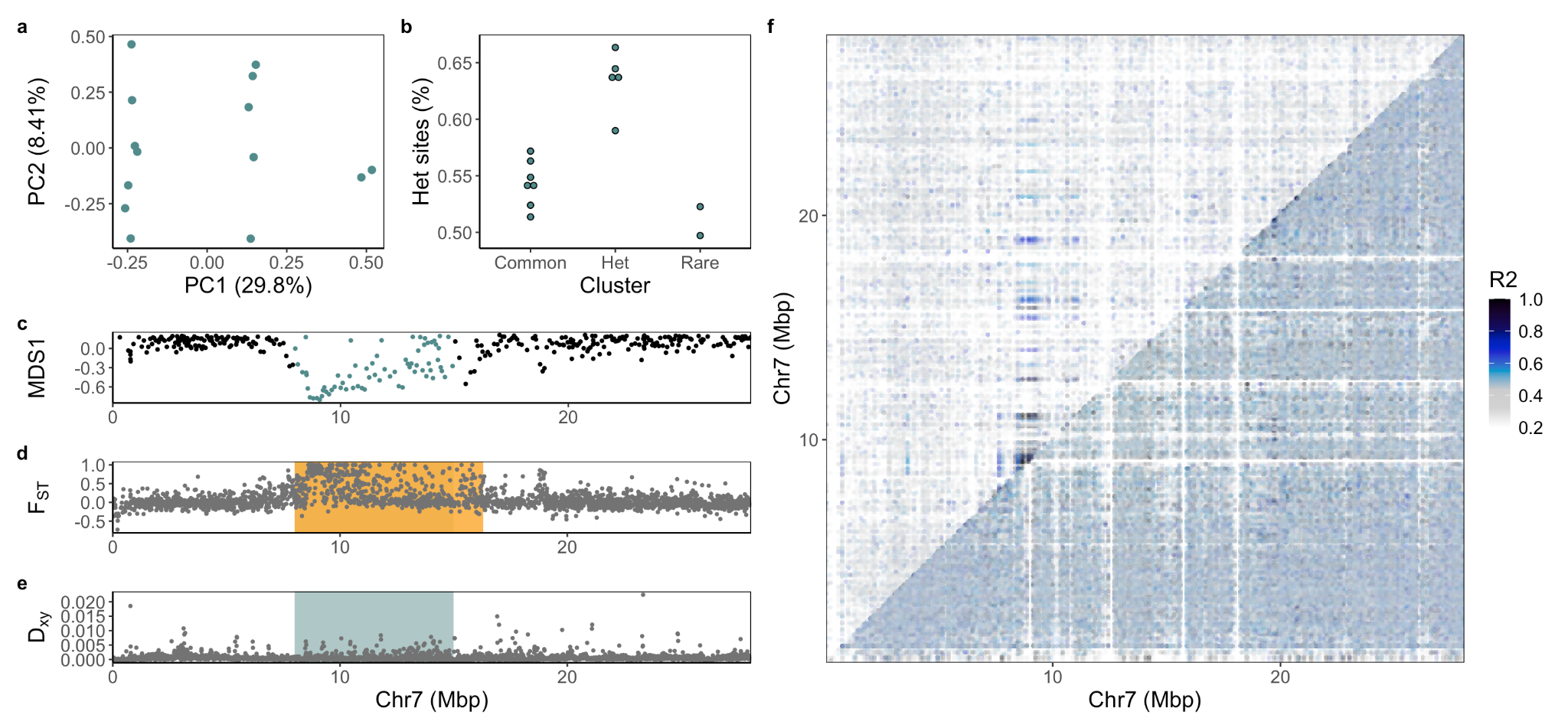


**Figure S39.** Inversion detected on chromosome 7 (Arctic cod Chr10) using polar cod as reference genome. a) PCA for the inversion region identified using lostruct. b) Manually assigned cluster groups and % heterozygous sites given in bins for the clusters. c) MDS analysis produced by lostruct where the inversion region is highlighted. d) F_ST_ and e) D_XY_ calculated with pixy showing elevated values within the highlighted inversion region. f) pairwise linkage disequilibrium plot calculated using pixy where the top triangle includes all samples, and the lower triangle includes only the individuals within the common type.


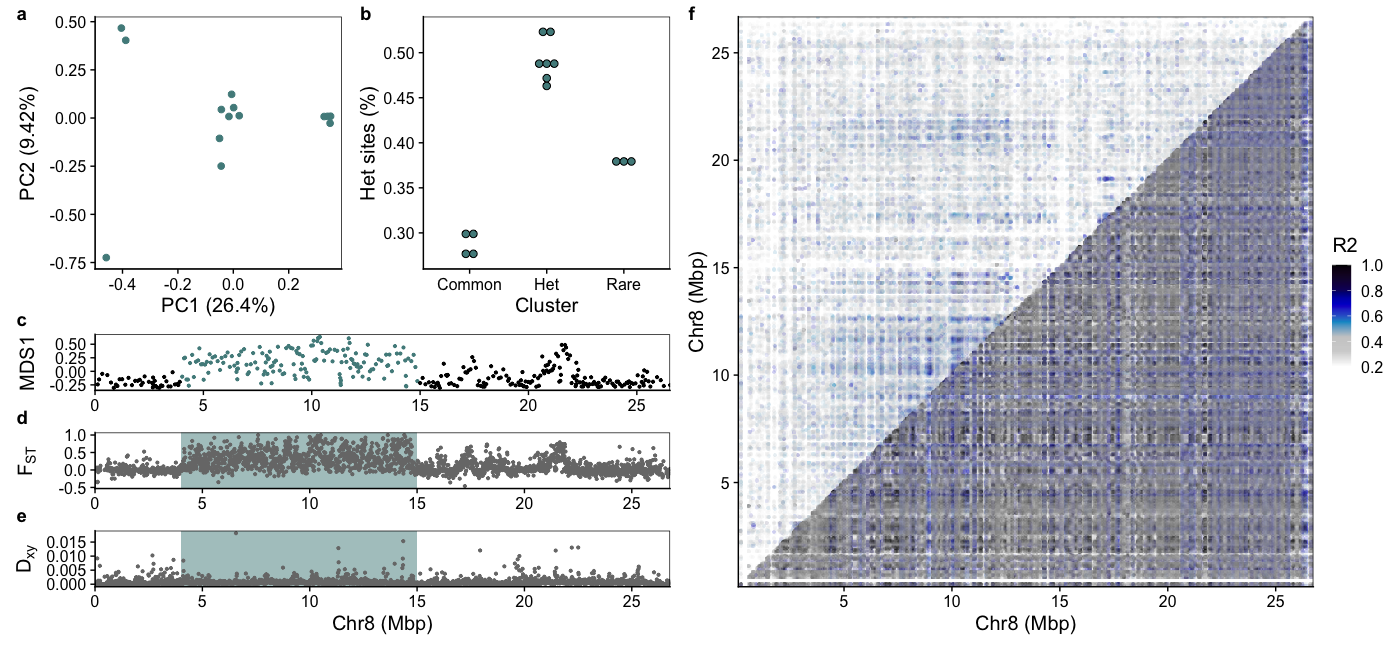


**Figure S40.** Inversion detected on chromosome 8 (Arctic cod Chr11) using polar cod as reference genome. a) PCA for the inversion region identified using lostruct. b) Manually assigned cluster groups and % heterozygous sites given in bins for the clusters. c) MDS analysis produced by lostruct where the inversion region is highlighted. d) F_ST_ and e) D_XY_ calculated with pixy showing elevated values within the highlighted inversion region. f) pairwise linkage disequilibrium plot calculated using pixy where the top triangle includes all samples, and the lower triangle includes only the individuals within the common type.


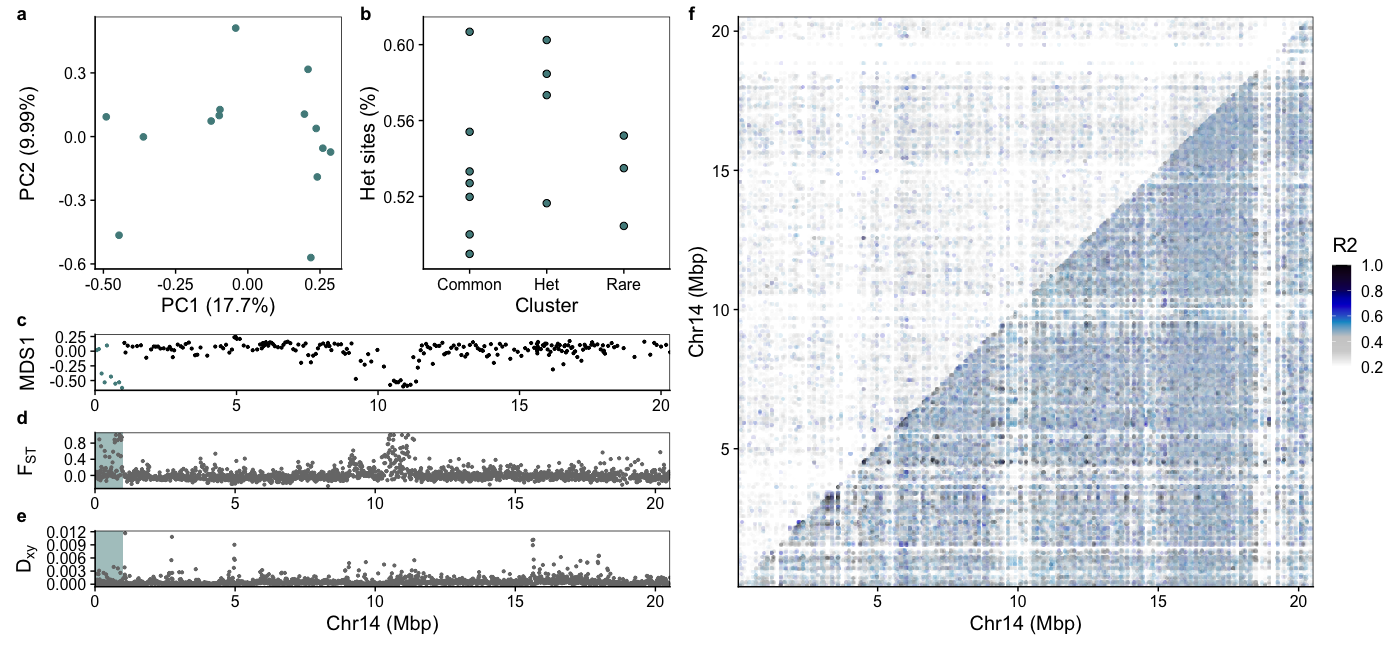


**Figure S41.** Split inversion detected on chromosome 14 (Arctic cod Chr13) using polar cod as reference genome. a) PCA for the inversion region identified using lostruct. b) Manually assigned cluster groups and % heterozygous sites given in bins for the clusters. c) MDS analysis produced by lostruct where the inversion region is highlighted. d) F_ST_ and e) D_XY_ calculated with pixy showing elevated values within the highlighted inversion region. f) pairwise linkage disequilibrium plot calculated using pixy where the top triangle includes all samples, and the lower triangle includes only the individuals within the common type.


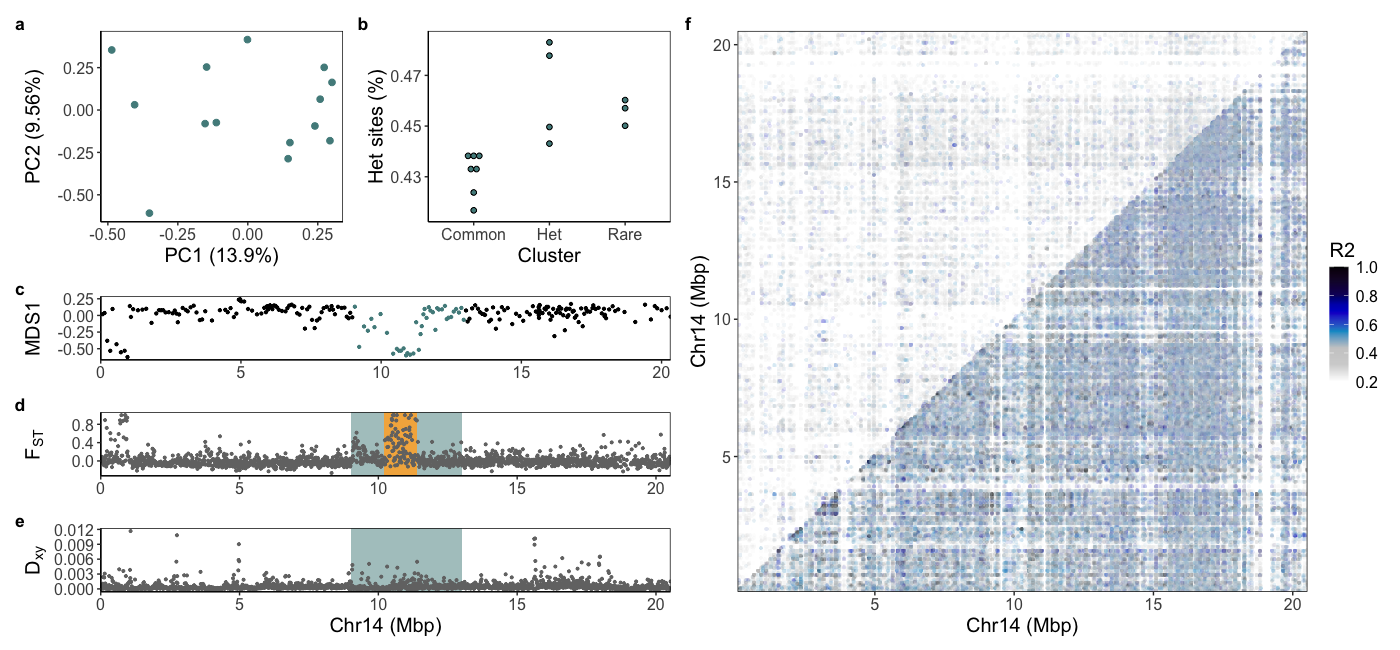


**Figure S42.** Split inversion detected on chromosome 14 (Arctic cod Chr13) using polar cod as reference genome. a) PCA for the inversion region identified using lostruct. b) Manually assigned cluster groups and % heterozygous sites given in bins for the clusters. c) MDS analysis produced by lostruct where the inversion region is highlighted. d) F_ST_ and e) D_XY_ calculated with pixy showing elevated values within the highlighted inversion region. f) pairwise linkage disequilibrium plot calculated using pixy where the top triangle includes all samples, and the lower triangle includes only the individuals within the common type.


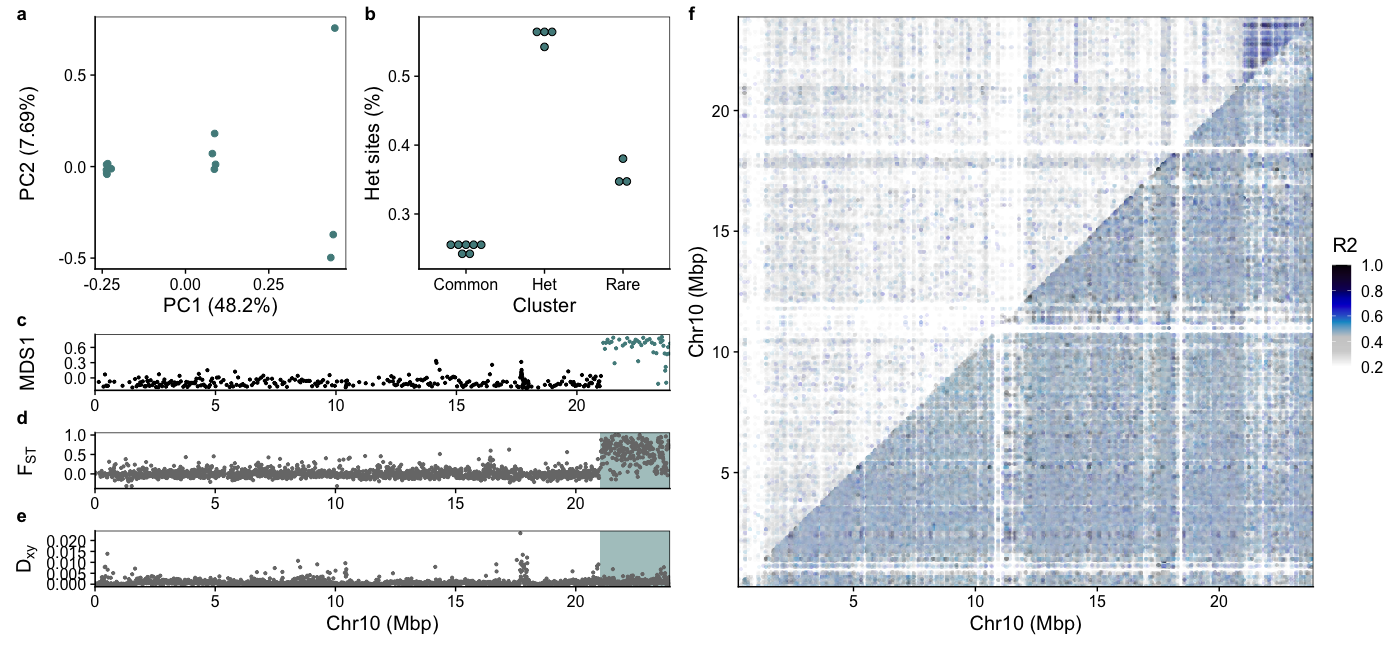


**Figure S43.** Inversion detected on chromosome 10 (Arctic cod Chr14) using polar cod as reference genome. a) PCA for the inversion region identified using lostruct. b) Manually assigned cluster groups and % heterozygous sites given in bins for the clusters. c) MDS analysis produced by lostruct where the inversion region is highlighted. d) F_ST_ and e) D_XY_ calculated with pixy showing elevated values within the highlighted inversion region. f) pairwise linkage disequilibrium plot calculated using pixy where the top triangle includes all samples, and the lower triangle includes only the individuals within the common type.


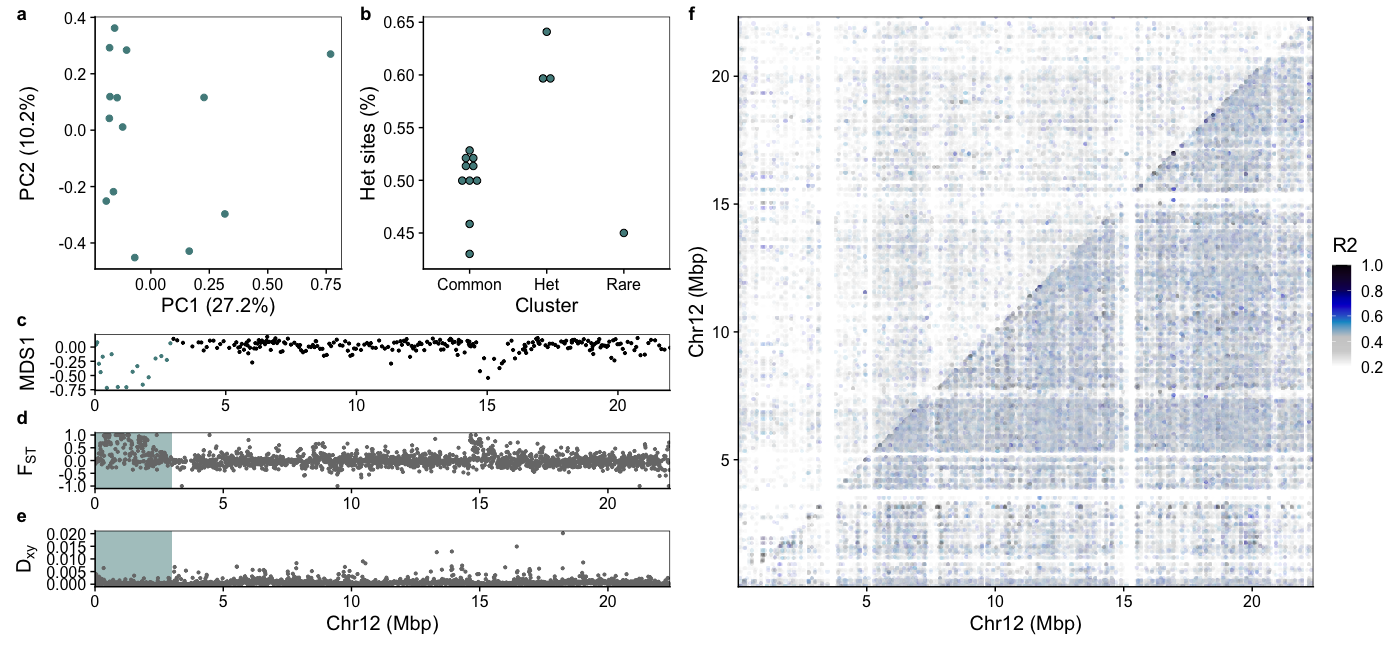


**Figure S44.** Putative inversion detected on chromosome 12 (Arctic cod Chr15) using polar cod as reference genome. a) PCA for the inversion region identified using lostruct. b) Manually assigned cluster groups and % heterozygous sites given in bins for the clusters. c) MDS analysis produced by lostruct where the inversion region is highlighted. d) F_ST_ and e) D_XY_ calculated with pixy showing elevated values within the highlighted inversion region. f) pairwise linkage disequilibrium plot calculated using pixy where the top triangle includes all samples, and the lower triangle includes only the individuals within the common type.
