# Supplementary material for: Reference genome bias in light of species-specific chromosomal reorganization and translocations
Source: Genome Biol. 2025 Oct 15;26:355. doi: 10.1186/s13059-025-03761-w (PMC12523119; doi:10.1186/s13059-025-03761-w)
Supplement: Supplementary file 6 — Additional file 6: Supplementary Sequencing Report. [file 13059_2025_3761_MOESM6_ESM.docx]

### **Additional file 6**

### **Supplementary Sequencing Report**

DNA samples were processed and sequenced by the Norwegian Sequencing Centre (https://www.sequencing.uio.no). The DNA samples were quantified using the FLUOStar Optima (BMG Labtech) with the Qubit dsDNA HS Assay Kit chemistry (ThermoFisher Scientific). Normalization of all DNA samples to 20ng/ul with Elution Buffer (Qiagen) was performed using the Sciclone G3 NGS Workstation (Perkin Elmer). Normalized DNA samples were sheared using the E220 focused-ultrasonicator (Covaris) with the appropriate manufacturer ́s settings for a target fragment mean size of 350bp. After shearing all samples were purified and size selected using KAPA Pure beads (Roche) in a ratio 0.8x (beads:sample) in order to remove fragments shorter than 200bp prior to library preparation.

Library preparation was performed using the KAPA Hyper kit (Roche) on Mosquito LV (Low Volume) pipetting robot (sptlabtech). The library preparation reactions (End repair, A-tailing and adapter Ligation) were performed using 5x reduced volume compared to the kit reaction volumes. The IDT for Illumina TruSeq DNA UD 96 Indexes (Illumina) were used for barcoding each 96-plate of samples. After the ligation of adapters, the samples volume was increased to 25ul with the addtion of EB buffer (Qiagen), and one round of bead cleanup with ratio 0.8x was performed. The libraries were subsequently amplified with 5 cycles of PCR. The PCR reactions were done in 2x reduced volume compared to the kit PCR reactions. All incubations were executed according to the manufacturer's instructions. The final libraries were purified, and size selected using KAPA Pure beads (Roche) in a ratio 0.8x.

After library preparation and cleanup, all libraries were run on a 5200 Fragment Analyzer System (Agilent) using the NGS Fragment Kit: DNF-473-0500 (Agilent) for determination of the average size of each library. Subsequently, absolute quantification of each library was done using the KAPA Library Quanitification Kits (Roche), on a LightCycler 480 qPCR instrument (Roche) in 10ul reaction volume. Finally, after determining the absolute concentration of each library (in nM) using the Fragment analyzer and qPCR results, all libraries were normalized to the same molarity using the Sciclone G3 NGS Workstation (Perkin Elmer) and equal volumes of each sample were pooled, creating 96plex pools. Each of the pools was sequenced on several lanes of a HiSeq4000 System (Illumina) in 2x150bp mode (150bp Paired End), using a HiSeq 3000/4000 SBS Kit (300 cycles) (Illumina).
